# Transport Matters: The Critical Role of the Hydrogen Evolution Reaction (HER) in Accelerating Electrochemical Nitrate to Ammonia Conversion

**DOI:** 10.1002/advs.202506733

**Published:** 2025-09-23

**Authors:** Nandu Ashtaman‐Pillai Syamaladevi, Abhijit Dutta, Alain Rieder, Xin Yu, Hridya Nedumkulam, Jakub Drnec, Zsolt Szakály, Soma Vesztergom, Rebecca Katharina Pittkowski, Peter Broekmann

**Affiliations:** ^1^ Department of Chemistry Biochemistry and Pharmaceutical Sciences University of Bern Freiestrasse 3 Bern 3012 Switzerland; ^2^ NCCR Catalysis University of Bern Freiestrasse 3 Bern 3012 Switzerland; ^3^ European Synchrotron Radiation Facility (ESRF) 71 Avenue des Martyrs Grenoble 38000 France; ^4^ MTA–ELTE Momentum Interfacial Electrochemistry Research Group Eötvös Loránd University Pázmány Péter sétány1/A Budapest 1117 Hungary; ^5^ Center for High Entropy Alloy Catalysis Department of Chemistry University of Copenhagen Universitetsparken 5 Copenhagen 2100 Denmark

**Keywords:** cobalt composite foam, green ammonia synthesis, HER‐mediated self‐convection, mass transport, nitrate reduction, operando spectroscopy

## Abstract

A porous Co‐based metal‐oxide foam catalyst is fabricated via the dynamic hydrogen bubble template electrodeposition method followed by calcination (6 h at 300 °C thermal treatment). Electrolysis results demonstrate excellent performance of this catalyst in the electrochemical nitrate reduction reaction (${\mathrm{NO}}_{3}^{-}\mathrm{RR}$), attaining near‐unity Faradaic efficiency (97.8% ± 3.6% at j_NH3,lim_ = –59.5 ± 2.3 mA cm^−2^) at a low (over)potential of –0.2 V vs RHE, which represents maximum achievable performance in 0.1 mol L^−1^ nitrate solutions (*p*H 13.7) under transport‐limiting conditions in the absence of extra convection. Digital simulations show that, without forced convection, the catalyst's electrochemically active surface area changes dynamically due to rapid nitrate depletion inside the 3D foam. Electrolyte replenishment, triggered by vigorous hydrogen evolution, is shown to restore the active surface in the foam interior. This ***self‐convection*** enables high ammonia partial current densities exceeding hundreds of mA cm^−2^ (e.g., j_NH3_ = –220 ± 18 mA cm^−2^ at –0.6 V vs RHE, with FE_NH3_ = 80.2% ± 2.2%). *Operando* XAS, XRD, Raman spectroscopy, and electrochemical analysis reveal the in situ evolution of a “***tandem***” composite catalyst during electrolysis, where β‐Co(OH)_2_ and metallic Co function both as the active phases for ${\mathrm{NO}}_{3}^{-}\mathrm{RR}$, with β‐Co(OH)_2_ remaining kinetically stabilized under the cathodic operating conditions.

## Graphical abstract

A porous cobalt‐based metal‐oxide foam catalyst is synthesized using the DHBT technique, followed by calcination. It demonstrates exceptional activity for *e*‐NO3RR, reaching near‐unity ammonia selectivity at low overpotentials. Dynamic surface area changes due to NO3‐ depletion are mitigated by “***self‐convection***” during hydrogen evolution. *Operando analyses* reveal the formation of highly active “***tandem catalyst***”— β‐Co(OH)_2_ and metallic Co serving as a stable active phase under reaction conditions.

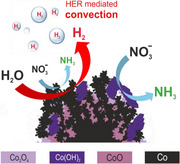

## 1 Introduction

In recent years, research in the field of electrocatalysis has increasingly addressed the disruption of the global nitrogen cycle caused by anthropogenic influences.^[^ ^1^ ^]^ Excessive use of nitrogenous fertilizers, such as ammonium nitrate, ammonium sulfate, and urea causes a persistent accumulation of oxidized nitrogen species, such as nitrates and nitrites in surface water, groundwater, and soil.^[^ ^1^, ^2^ ^]^ High nitrate concentrations of up to 1500 mg L^−1^ have been reported in heavily polluted ground and surface water bodies.^[^ ^3^ ^]^ As evidenced by the extant literature, nitrate contaminations have been associated with a number of deleterious effects on human health, including methemoglobinemia in children and gastrointestinal cancer in adults.^[^ ^4^ ^]^

Electrochemical reduction of nitrate (hereafter referred to as ${\mathrm{NO}}_{3}^{-}\mathrm{RR}$) is considered a viable wastewater treatment concept with a high potential to contribute to a future circular nitrogen economy.^[^ ^5^ ^]^ This electrolysis process can convert environmentally harmful nitrate back into ammonia, with nitrite (${\mathrm{NO}}_{2}^{-}$) often occurring as an intermediate product, according to the following equations:^[^ ^6^ ^]^

(1) $${\mathrm{NO}}_{3}^{-}+H_{2}O+2e^{-}\to {\mathrm{NO}}_{2}^{-}+2OH^{-}$$

and

(2) $${\mathrm{NO}}_{2}^{-}+5H_{2}O+6e^{-}\to NH_{3}+7OH^{-}$$

However, it is important to note that large‐scale electrochemical ammonia synthesis may not be economically feasible if the nitrate reactants originate from contaminated ground and surface waters due to the low nitrate concentrations in these water bodies. Van Langevelde et al.^[^ ^5a^ ^]^ emphasized that contaminated groundwater bodies typically contain nitrate only in the lower or even sub‐millimolar concentration range. This requires costly pre‐treatment to capture and concentrate the nitrate from these diluted bodies of water prior to the electrolysis process. Point sources with highly concentrated nitrate effluents (*c*_nitrate_ > 0.1 M) are therefore more attractive from an economic and technical point of view, as can be found worldwide, for example, in the nuclear industry^[^ ^7^ ^]^ with nitrate wastewaters even exceeding nitrate concentrations of 1 mol L^−1^.^[^ ^5a^ ^]^

A variety of different ${\mathrm{NO}}_{3}^{-}\mathrm{RR}$ catalysts, including monometallic,^[^ ^8^ ^]^ alloyed systems,^[^ ^8^, ^9^ ^]^ composites,^[^ ^10^ ^]^ and oxide materials,^[^ ^8^, ^10^ ^]^ is today available that can, at least under idealized laboratory conditions, convert nitrate into ammonia with near‐unity Faradaic efficiencies (*FE*_NH3_).^[^ ^11^ ^]^ Industrially relevant partial current densities for ammonia conversion (*j*_NH3_) exceeding 1 A cm^−2^ were also reported,^[^ ^11^, ^12^ ^]^ although it has to be noted that the achievement of such high currents, somewhat contrary to what is often claimed in literature, should not be ascribed to the (albeit high) performance of the employed catalyst but rather to enhanced modes of transport that can be reached, e.g., by stirring (as in classical batch‐type reactors^[^ ^9^, ^13^ ^]^), or by employing flow cell designs which can be fluidic^[^ ^14^ ^]^ or MEA^[^ ^9d,e^ ^]^ based.

To illustrate this issue, **Table** **1** lists diffusion layer thickness values (*δ*) estimated based on the work by Ibl et al.^[^ ^15a^ ^]^ for planar electrodes subjected to different hydrodynamic conditions. Limiting current densities are approximated using the equation.

(3) $$j_{\lim}=n_{e^{-}}\;D\;F\;\frac{c_{\mathrm{bulk}}}{\delta }$$

The formation of three different products (nitrite, dinitrogen, and ammonia) are also listed in Table 1 and are plotted vs δ in Figure S1 (Supporting Information). For the calculations, a diffusion coefficient of *D* = 1.6 · 10^−5^ cm^2^ s^−1^ and a bulk concentration of 0.1 mol L^−1^ was assumed for nitrate ions. As illustrated in Table 1, in the absence of externally applied hydrodynamic forces, the limiting current densities of ${\mathrm{NO}}_{3}^{-}\mathrm{RR}$ to ammonia remain within the range of milliamperes per square centimeter. This range represents a physical limit for achievable ${\mathrm{NO}}_{3}^{-}\mathrm{RR}$ rates in the absence of any kind of extra hydrodynamic force. The extreme ${\mathrm{NO}}_{3}^{-}\mathrm{RR}$ rates often observed but ambiguously described in literature must therefore be explained by assuming an internal stirring effect — such as that caused by cathodic hydrogen evolution reaction (HER), which results in vigorous effervescence near the electrode surface and increased limiting current densities (Table 1). Naturally, having HER superimposed on ${\mathrm{NO}}_{3}^{-}\mathrm{RR}$ comes at the cost of reducing the *FE* of ammonia production. However, in absolute terms, the yield (partial current density) of nitrate‐to‐ammonia reduction could still be considerably increased by triggering HER.

**Table 1:** Diffusion layer thicknesses (δ) and limiting current densities (*j_lim_*) of nitrate reduction yielding various products for a few typical hydrodynamic scenarios according to Ibl et al.^[^ ^15a^ ^]^ The numerical values were derived by assuming a nitrate concentration of 0.1 mol L^−1^, which has been used throughout this work.

System	*δ* / µm	*j*_lim_ / [A cm^−2^]		
		to ${\mathrm{NO}}_{2}^{-}$ (2 e^–^)	to N_2_ (5 e^–^)	to NH_3_ (8 e^–^)
**Convection‐free electrode,** after 2 h of electrolysis	4750	0.000650	0.00163	0.00260
**Natural convection,** vertical planar electrode upward‐facing horizontal electrode	200 80	0.0154 0.0386	0.0386 0.0965	0.0618 0.154
**Laminar flow‐by,** planar electrode, rate of 25 cm s^−1^	100	0.0309	0.0772	0.124
**Rotating disk,** 200 rpm 1200 rpm	29 19	0.108 0.1162	0.271 0.404	0.433 0.647
**Gas evolving electrode,** 1 cm min^−1^ 13 cm min^−1^	15 4	0.206 0.772	0.514 1.93	0.823 3.09

The self‐convective effect is particularly important for highly porous 3D catalysts, where nitrate reactant depletion inside narrow pores can occur even at moderate current densities, far below the ampere range.^[^ ^16^, ^17^ ^]^ Such reactant depletion, occurring in the absence of extra nitrate supply, can turn the assumed benefits of applying a porous, high surface area ${\mathrm{NO}}_{3}^{-}\mathrm{RR}$ catalyst into a conceptual disadvantage as the competing HER is not limited by mass transport when carried out under *p*H‐neutral or alkaline conditions. This has recently been demonstrated using (mono)metallic Cu (foam) catalysts,^[^ ^17^ ^]^ which are considered model systems where the cathodic transformation of their oxidic precursors (that is, catalyst activation) is fast and already completed at potentials prior to the ${\mathrm{NO}}_{3}^{-}\mathrm{RR}$ onset. In this specific case, nitrate reduction and HER take place on both the (mono)metallic Cu surface in the absence of corresponding surface oxides and hydroxides, in full agreement with DFT calculations^[^ ^16^ ^]^ and thermodynamic considerations.^[^ ^17^ ^]^

In this contribution, novel Co‐based metal‐oxide foam materials with excellent ${\mathrm{NO}}_{3}^{-}\mathrm{RR}$ performance characteristics are presented. These materials can be considered as counterexamples to the (mono)metallic Cu (foam) systems, since their multistage metal oxide transitions occur in alkaline electrolyte solutions at more cathodic potentials far beyond the expected thermodynamic reduction potentials. Due to the sluggish reduction kinetics, Co‐oxide/hydroxide reduction processes are superimposed on ${\mathrm{NO}}_{3}^{-}\mathrm{RR}$ and HER, thus leading to Co species of different oxidation number co‐existing on the active catalyst surface during ${\mathrm{NO}}_{3}^{-}\mathrm{RR}$. This also explains the highly controversial discussion in the literature on the chemical nature of Co‐based ${\mathrm{NO}}_{3}^{-}\mathrm{RR}$ catalysts. One group of authors assumes that the spinel Co_3_O_4_ with mixed Co(III) and Co(II) species acts as the catalytically active phase for ${\mathrm{NO}}_{3}^{-}\mathrm{RR}$,^[^ ^18^ ^]^ while others claim that the Co(II) containing β‐Co(OH)_2_ is the most essential catalyst component in ${\mathrm{NO}}_{3}^{-}\mathrm{RR}$.^[^ ^5^, ^12^, ^19^ ^]^ Also, cobalt in its fully reduced metallic form has been hypothesized as an active catalyst material for ${\mathrm{NO}}_{3}^{-}\mathrm{RR}$.^[^ ^18^, ^20^ ^]^ In addition, various Co‐based “tandem” catalysts have been proposed, in which an additional co‐catalyst is deposited with the main catalyst species and is assumed to facilitate certain sluggish reaction steps in the multistep reaction pathway (e.g., the hydrogenation steps).

In this study, it is demonstrated that Co‐based composite materials exhibit particularly high activity toward ${\mathrm{NO}}_{3}^{-}\mathrm{RR}$ when ammonia‐forming β‐Co(OH)_2_ is combined with metallic Co, on which not only ${\mathrm{NO}}_{3}^{-}\mathrm{RR}$ but also HER can take place. This combination facilitates (hydrogen) gas evolution, thereby promoting nitrate mass transport into the pores of the foam‐type catalyst. The co‐existence of metallic Co and β‐Co(OH)_2_ becomes possible only due to a substantial kinetic stabilization of the β‐Co(OH)_2_ under ${\mathrm{NO}}_{3}^{-}\mathrm{RR}$ conditions as demonstrated herein by highly complementary *operando* analytical techniques and electrochemical analysis.

## 2 Results and Discussion

### 2.1 The Catalyst Precursor

**Figure 1:**
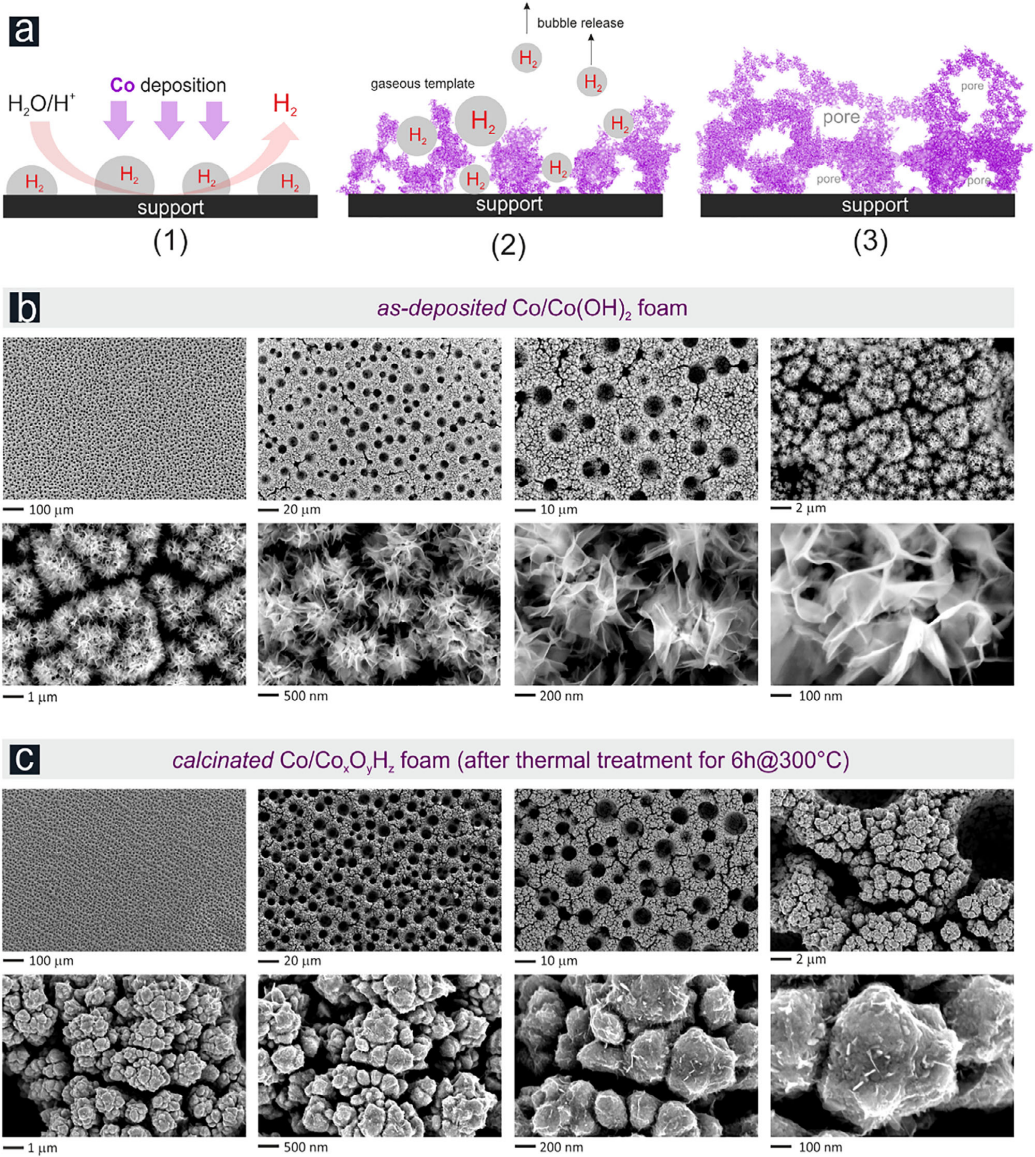
a) Scheme illustrating the principle of DHBT‐assisted Co foam deposition (DHBT: dynamic hydrogen bubble template). b) Top‐down SEM images of increasing magnification showing the as‐deposited Co/Co(OH)_2_@Ni foam (20 s deposition at –3 A cm^−2^) prior to calcination (thermal treatment of 6h@300 °C in air). c) Top‐down SEM images of the Co/Co*_x_* O*_y_* H*_z_* @Ni foam after calcination, used in this study as the catalyst precursor.

**Figure** **1**a depicts the basic principle of the dynamic hydrogen bubble template (DHBT) assisted metal foaming process,^[^ ^21a–c^ ^]^ which is based on the superposition of a primary metal electrodeposition with the hydrogen evolution reaction (HER) according to the following equations:

(4) $$Me^{n+}+n\;e^{-}\to \mathrm{Me}$$

(5) $$2\;H^{+}+2\;e^{-}\to \;H_{2}\;\left(\mathrm{proton}\;\mathrm{reduction}\right)$$

(6) $$2H_{2}O+2\;e^{-}\to \;H_{2}+2\;OH^{-}\;\left(\mathrm{reductive}\;\mathrm{water}\;\mathrm{splitting}\right)$$

Under harsh experimental conditions, e.g., at the applied geometric current densities of –3 A cm^−2^, the gas evolving HER occurs at mild acidic conditions of *p*H 4.5 through both proton reduction, Equation (5), and reductive water splitting, Equation (6), thereby creating the temporary geometric template on the electrode surface in the form of gas bubbles necessary for the metal foaming process. A rather uniform Co deposit forms on the Ni support on a macroscopic length, revealing a multi‐level architecture of open and interconnected pores on the nm and µm length‐scale (Figure 1b). The thickness of the Co deposit and the resulting pore size distribution crucially depend on both the specific deposition time and the applied current density (Figures S5–S7, Supporting Information). Co deposits following 20 s of electrodeposition, used as standard for further catalyst activation and subsequent testing, feature a thickness of ≈20 µm, surface pore diameters in the range of 4–10 µm, and a Co mass loading of ca. 4 mg cm^−2^ (Table S1, Supporting Information). The side‐walls of the 3D foam architecture are also porous and resemble a characteristic cauliflower morphology on a submicron length scale. On the nm scale, the Co deposit is covered with 2D layered structures, which can be assigned to an irregular, defect‐rich β‐Co(OH)_2_ phase^[^ ^22^ ^]^ that originates from surface oxidation after initial Co deposition and subsequent CoO hydrolysis during the subsequent cleaning and drying process. β‐Co(OH)_2_ displays a 2D sheet structure (triclinic P1 space group, brucite/Mg(OH)_2_‐type) in which individual Co^2^⁺ ions are coordinately bound to six equivalent O^2–^ anions, forming edge‐sharing octahedra.^[^ ^22b^ ^]^ The presence of surface Co(OH)_2_ is further supported by ex situ Raman spectroscopy (Figure S10a, Supporting Information) showing characteristic Raman features at 461 cm^−1^ (A_1g_), 522 cm^−1^ (A_2u_), and 690 cm^−1^ (E_g_) in full agreement with respective Co(OH)_2_ reference spectra from literature^[^ ^23^ ^]^ (Figure S10b, Supporting Information). Due to the observed Co(OH)_2_ surface termination, the as‐deposited Co foams are denoted Co/Co(OH)_2_@Ni and Co/Co(OH)_2_@C, respectively.

The catalyst precursor preparation was completed by a thermal treatment (calcination) step applying 300 °C in air for 6 h. Top‐down SEM inspection reveals that there is no significant change in the 3D foam morphology on the larger‐than‐µm length scale whereas structural features on the nm length scale, assigned to the β‐Co(OH)_2_ platelets, disappeared almost completely after thermal treatment (Figure 1c).

**Figure 2:**
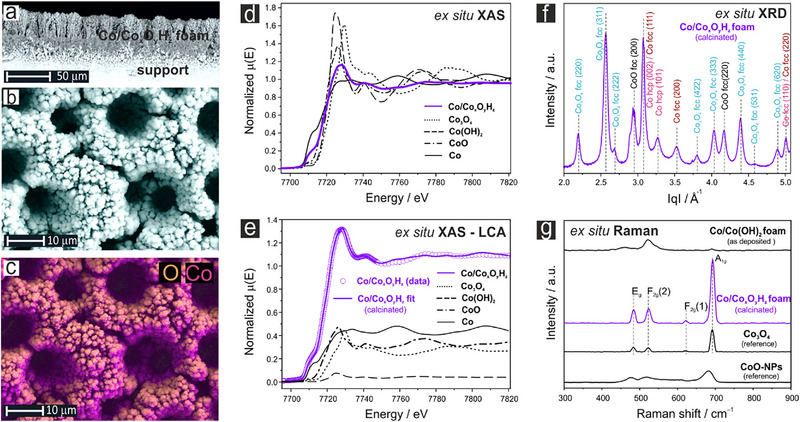
Chemical composition and morphology of the Co/Co*_x_* O*_y_* H*_z_* foam used as catalyst precursor. This state represents the calcinated Co/Co*_x_* O*_y_* H*_z_* foam *prior* to the electrolysis. a) Cross‐sectional SEM image. b) Top‐down SEM image. c) Corresponding SEM/EDX image highlighting the spatial distribution of cobalt and oxygen (see also Figure S16, Supporting Information). d) Ex situ XAS data of the Co/Co*_x_* O*_y_* H_z_ foam overlaid with reference spectra for metallic Co, Co_3_O_4_, Co(OH)_2_, and CoO. e) Linear combination analysis (LCA) of the measured ex situ XAS data revealing the chemical composition of the Co/Co*_x_* O*_y_* H*_z_* foam (catalyst precursor). Note that the indexed Co hcp(002) and Co fcc(111) peaks also overlap with the Co_3_O_4_ fcc(400) peak. f) Corresponding synchrotron‐based ex situ XRD characterization of the Co/Co*_x_* O*_y_* H*_z_* foam measured in transmission mode. g) Corresponding ex situ Raman spectroscopic characterization of the Co/Co*_x_* O*_y_* H*_z_* foam including reference spectra for Co_3_O_4_, Co(OH)_2_, and CoO.

A more comprehensive ex situ characterization of the calcinated foam, denoted Co/Co*_x_* O*_y_* H*_z_* @Ni or Co/Co*_x_* O*_y_* H*_z_* @C, is presented in **Figure** **2**. This state represents the chemical characteristics of the catalyst precursor *prior* to the actual electrolysis. Panels a–c in Figure 2 display cross‐sectional (a) and top‐down (b) SEM images of the catalyst precursor, along with a corresponding spatially resolved EDX mapping of the cobalt and oxygen (panel c). Note that the intensity distribution for Co and O displayed in the 2D EDX map (Figure 2c) is also affected by the porous nature of the foam material. Relevant EDX point spectra are presented in Figure S16 (Supporting Information).

Cross‐section SEM images are typically used to estimate the thickness of the foam, which is typically in the range of ca. 20 µm for the catalysts used in this work (initial 20 s Co deposition, Table S1, Supporting Information).

Ex situ XAS measurements (Co K‐edge) and corresponding linear combination analysis (LCA) reveal a complex metal‐oxide composite nature of the catalyst precursor composed of Co_3_O_4_, CoO, Co(OH)_2_, and metallic Co. Note that the annealing temperature and time of the calcination step were optimized such that metallic Co remained as the main component of the bulk Co/Co*_x_* O*_y_* H*_z_* composite material. This aspect was considered particularly important as lower metal and higher oxide contents typically result in significant conductivity losses, which are detrimental to further in situ electrochemical activation of the catalyst precursor, leading to insufficient current densities and Faradaic yields for ammonia in the electrolysis experiments. A four‐component LCA fit, based on XAS reference measurements for Co, CoO, Co(OH)_2_, and Co_3_O_4_ (Figure 2d; Figure S17, Supporting Information), revealed LCA weights of 43.1% for metallic Co, 3.6 % for Co(OH)_2_, 29.7 % for CoO and 24.0 % for Co_3_O_3_ (see also Figure S18, Supporting Information). A low abundance of Co(OH)_2_ is indeed expected as the hydroxide forms solely on the Co/Co*_x_* O*_y_* H*_z_* foam surface as hydrolysis product of CoO (Figure S11, Supporting Information). It needs to be emphasized that the quality of fit does not substantially change when the LCA is performed only with three components omitting the Co(OH)_2_ as the minor Co(II) component. This is mainly due to the fact that the XAS characteristics for Co(OH)_2_ and CoO are almost indistinguishable (see Figure S17, Supporting Information). Complementary synchrotron‐based XRD measurements of the Co/Co*_x_* O*_y_* H*_z_* foam, sensitive solely to the crystalline components, confirm the composite nature of the catalyst precursor and the presence of crystalline Co_3_O_4_ (*Fd3̅m* space group, spinel),^[^ ^24^ ^]^ CoO (*Fm3̅m* space group, rock salt),^[^ ^24^ ^]^ and metallic Co whereas crystalline β‐Co(OH)_2_ (*P3̅m1* space group, layered‐P2)^[^ ^24^ ^]^ was not detected in the calcinated Co/Co*_x_* O*_y_* H*_z_* @C foam prior to the electrolysis (Figure 2f). A more comprehensive analysis of the ex situ XRD data is provided in Table S2 (Supporting Information). A Rietveld analysis yields a relative abundance of crystalline fcc Co (24%), hcp Co (44%), fcc CoO (23.8%), and fcc Co_3_O_4_ (8.1%). Note that the relative abundance of the individual components determined by XRD may differ from the corresponding XAS quantification as the latter also covers the amorphous components of the metal‐oxide composite lacking long‐range translational order (Figure S18, Supporting Information). A comparison to the XAS results indicates that in particular the Co_3_O_4_ abundance is underestimated in the XRD suggesting a largely amorphous nature of the Co_3_O_4_.

Complementary ex situ Raman analysis –the most surface sensitive technique applied here– of the Co/Co*_x_* O*_y_* H*_z_* @C foam, shown in Figure 2g, provides further evidence for the presence of Co_3_O_4_ on its surface. The three clearly discernable Raman bands at 485 cm^−1^ (E_g_), 523 cm^−1^ (F_2_g), and at 692 cm^−1^ (A_1g_) match the corresponding spectrum of the Co_3_O_4_ reference.^[^ ^25^ ^]^

It is important to note that Raman scattering from the Co_3_O_4_ spinel phase is most intense and, when present, obscures the corresponding Raman signatures of the CoO and Co(OH)_2_ phases, making them difficult to study by Raman spectroscopy under operando conditions.

### 2.2 Catalyst Performance Evaluation

**Figure 3:**
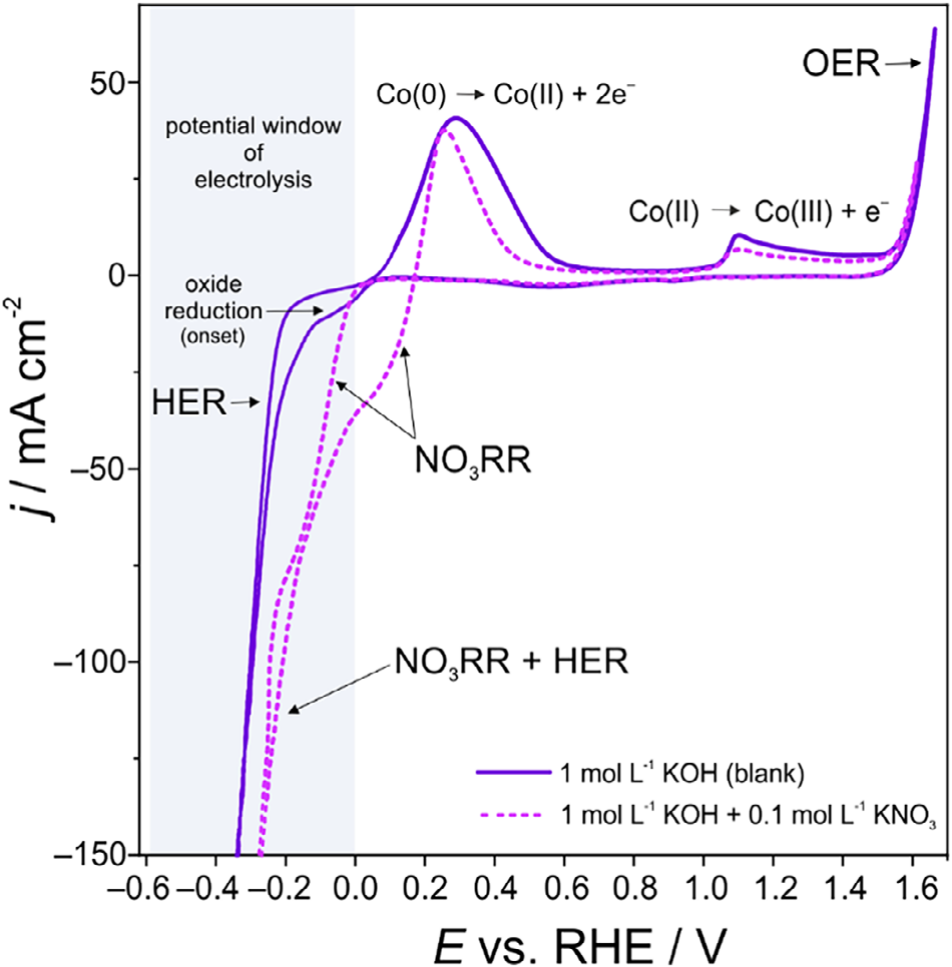
Representative steady‐state cyclic voltammograms (CVs) recorded at a sweep rate of dE/dt = 10 mV s^−1^ using the Co/Co*_x_* O*_y_* H*_z_* foam as the WE. The straight violet curve represents the blank CV (1 mol L^−1^ KOH solution, *p*H 13.7), whereas the dashed magenta curve depicts the CV recorded in the respective nitrate‐containing electrolyte solution (1 mol L^−1^ KOH + 0.1 mol L^−1^ KNO_3_, *p*H 13.7). The potential window used for the potentiostatic electrolysis experiments (see Figure 4) is highlighted in grey.

A comparison of stationary cyclic voltammograms (CVs) measured both in pure 1 mol L^−1^ KOH supporting electrolyte solution (*p*H of 13.7) and in the respective nitrate‐containing solution (after addition of 0.1 mol L^−1^ KNO_3_; *p*H of 13.7) hints to a ${\mathrm{NO}}_{3}^{-}\mathrm{RR}$ process starting well before the onset of the parasitic HER (**Figure** **3**). In general, this observation is consistent with thermodynamic considerations based on the Pourbaix diagrams of the aqueous nitrate/ammonia system (Figure S19b, Supporting Information). The voltammetric analysis also reveals that the ${\mathrm{NO}}_{3}^{-}\mathrm{RR}$ is superimposed on the reduction of Co oxides. Co oxide reduction involves an initial single electron Co(III)/Co(II) transition transforming the Co_3_O_4_ spinel phase into CoO/Co(OH)_2_ followed by its subsequent reduction through a two‐electron transfer into metallic Co(0).

A more systematic variation of the cathodic vertex potential in the voltammetric experiment demonstrates reductive processes assigned to ${\mathrm{NO}}_{3}^{-}\mathrm{RR}$ at *E* ≤ 0.0 V vs RHE and the appearance of an anodic peak, centered at ca. +0.24 V vs RHE, in the reverse potential scan (Figure S20, Supporting Information). According to the literature,^[^ ^26^ ^]^ this peak has to be attributed to the Co(0)/Co(II) re‐oxidation forming a CoO/Co(OH)_2_ passive (precipitation) layer. There was no indication for a massive dissolution of Co(II) in the form of soluble ${\mathrm{HCoO}}_{2}^{2-}$ as suggested by thermodynamic considerations for this highly alkaline *p*H (Figure S19a, Supporting Information). The minor oxidation peak, observed prior to the onset of the oxygen evolution reaction (OER), can be attributed to Co(II) to Co(III) transition.^[^ ^26b,c^ ^]^ Note that only a fraction of the Co(II) is oxidized to Co(III) as constituent of Co_3_O_4_ or surface CoOOH.^[^ ^27^ ^]^ The absence of a corresponding pronounced reduction feature in the reverse potential scan suggests a substantial kinetic stabilization of Co(III) species which is, as will be demonstrated below, in agreement with the obtained *operando* measurements.

Voltammetric forward and backward scans demonstrate a ca. 250 mV wide hysteresis in the potential range between –0.1 V and +0.15 V vs RHE (Figure 3; Figure S20, Supporting Information), thus pointing to an in situ activation of the Co/Co*_x_* O*_y_* H*_z_* catalyst precursor toward ${\mathrm{NO}}_{3}^{-}\mathrm{RR}$ through its partial reduction.

**Figure 4:**
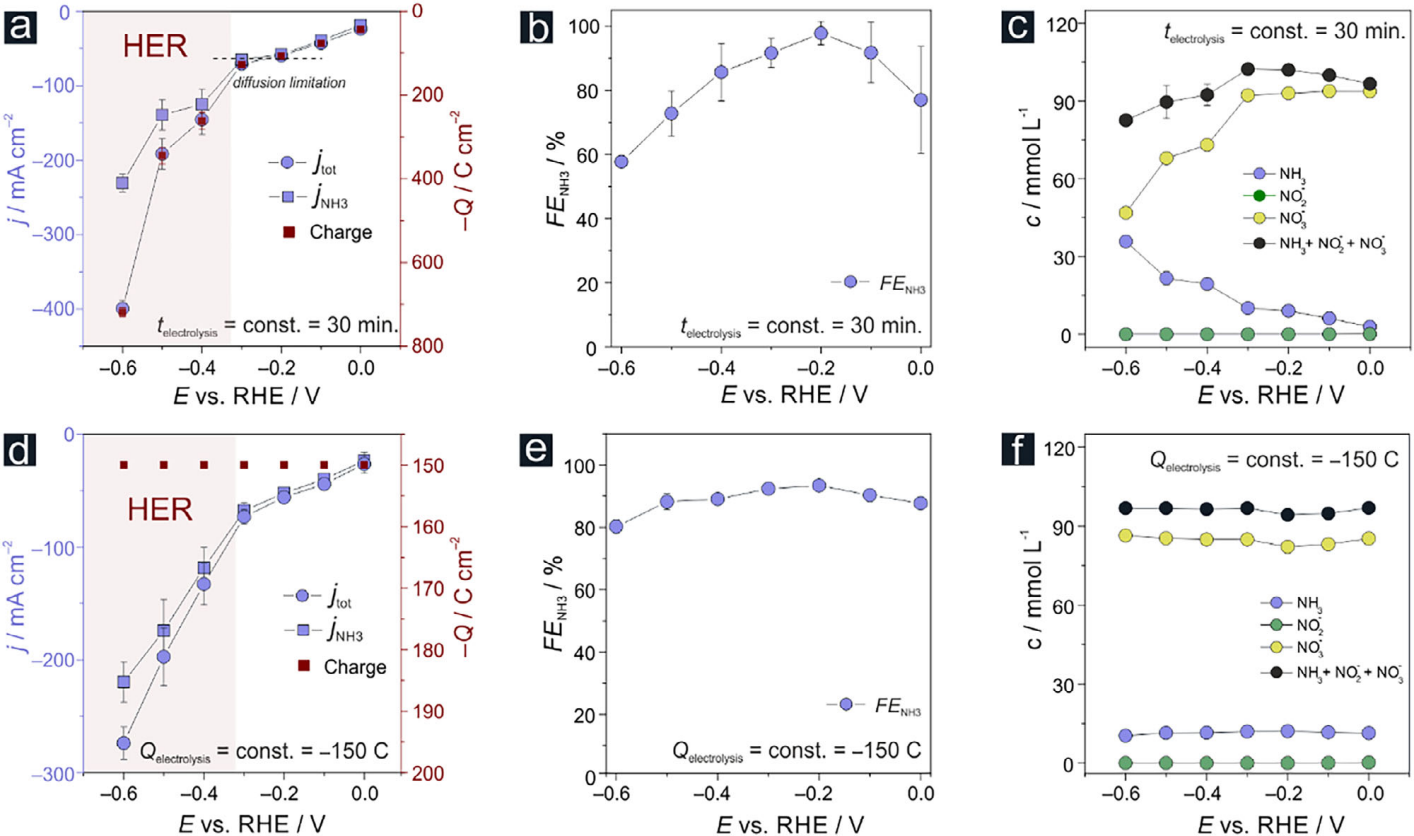
Electrocatalytic performance of the Co/Co*_x_* O*_y_* H*_z_* foam toward ${\mathrm{NO}}_{3}^{-}\mathrm{RR}$. a) (*j* vs *E* and *charge* vs *E*) plot of potentiostatic electrolyses performed in 1 mol L^−1^ KOH + 0.1 mol L^−1^ KNO_3_ solution (*p*H 13.7); the electrolyses were performed for constant time (*t*_electrolysis_) of 30 min. b) Corresponding (*FE*_NH3_ vs *E*) plot. c) Corresponding (*c* vs *E*) plot representing the electrolyte composition after the respective constant‐time electrolysis. d) (*j* vs *E and charge* vs *E*) plot of potentiostatic electrolyses performed in 1 mol L^−1^ KOH + 0.1 mol L^−1^ KNO_3_ solution (*p*H 13.7); electrolyses were performed by passing the same charge (*Q*_electrolysis_) of –150 C through the cell. e) Corresponding (*FE*_NH3_ vs *E*) plot. f) Corresponding (*c* vs *E*) plot representing the electrolyte composition after the respective constant‐charge electrolysis. Note that in panels a and d, the potential range corresponding to massive hydrogen gas evolution and associated convective effects is highlighted (see also Figure S23, Supporting Information).

After initial catalyst prescreening (Figure S21 and Table S3, Supporting Information), a systematic potential‐dependent performance testing of the Co/Co*_x_* O*_y_* H*_z_* @Ni catalyst precursor was carried out using two different approaches of potentiostatic electrolysis (**Figure** **4**; Figure S22, Supporting Information), based either on normalization to a constant electrolysis time (*t*_electrolysis_ = 30 min; panel a‐c) or to a constant charge passed through the cell during electrolysis (*Q*_electrolysis_ = –150 C, panel d–f). The potential range considered for the performance test (0 to –0.6 V vs RHE) can be subdivided into two characteristic domains in which ${\mathrm{NO}}_{3}^{-}\mathrm{RR}$ occurs either in the absence or in the presence of the competing and gas‐evolving HER (see also Figure S23 and Video S1, Supporting Information). The total (*j*_tot_) and corresponding partial current density for ammonia production (*j*_NH3_) remain comparatively low at electrolysis potentials of *E* ≥ –0.3 V vs RHE, but reaching nitrate diffusion limiting conditions (e.g., *j*_tot_ = (–59.5 ± 2.3) mA cm^−2^ and *j*_NH3_ = (–58.2 ± 4.4) mA cm^−2^ at –0.2 V vs RHE), consistent with an estimated diffusion layer thickness of ca. 200 µm, characteristic to natural convection (see Table 1 and Figure S1, Supporting Information).

A significant increase in these metrics is observed when the ${\mathrm{NO}}_{3}^{-}\mathrm{RR}$ becomes progressively superimposed on the HER at *E* ≤ –0.4 V vs RHE, reaching remarkable current density values of *j*_tot_ = (–399 ± 11) mA cm^−2^ and *j*_NH3_ = (–230 ± 11) mA cm^−2^ at –0.6 V vs RHE (Figure 4a, see Table S4, Supporting Information for the numerical data). The increasing dominance of the HER with negative going potentials is also evident in the corresponding (*FE*_NH3_ vs *E*) plot (Figure 4b) revealing a near unity ammonia Faradaic efficiency of *FE*_NH3_ = 97.8% ± 3.6% at *E* = –0.2 V vs RHE which gradually declines to *FE*_NH3_ = 57.7% ± 1.2% at –0.6 V vs RHE. When normalized to a constant electrolysis time, the transferred charges follow the expected trend of the potential‐dependent mean total current densities (Figure 4a; Figure S22, Supporting Information) and suggest an increasing reactant consumption with negative‐going potentials. The intended evaluation of catalyst performance, even after comparatively short electrolyses of 30 min duration, becomes already markedly influenced by reactant dilution and resulting mass transport effects, especially at the most negative electrolysis potentials applied (Figure 4c, see Table S4, Supporting Information for the numerical data). The observed decline in Faradaic efficiency to *FE*_NH3_ = 57.7% ± 1.2% at –0.6 V vs RHE (Figure 4b) is therefore more indicative of reactant depletion during electrolysis (Figure 4c) than of an actual loss of catalyst performance. A more appropriate approach to catalyst testing using common batch reactors with limited electrolyte volume is therefore based on constant‐charge electrolysis ensuring comparable reactant consumption during catalyst testing over the entire potential range considered (Figure 4d–f, see Table S4, Supporting Information for the numerical data). In fact, the significant decrease in ammonia efficiency in the HER regime, evident from Figure 4b, is substantially mitigated in the corresponding constant‐charge testing (Figure 4e), thus demonstrating a sufficient reactant supply during electrolysis even at high current densities, as a basis for a proper catalyst performance evaluation. This approach suggests that there is only marginal variation in intrinsic catalyst performance over the full range of electrolysis potentials applied when the Faradaic efficiency for ammonia (*FE*_NH3_) is used as the metric for performance evaluation (Figure 4e). The comparison of the constant‐time and constant‐charge screening approaches also reveals the consequences of the transition from a quiescent solution, i.e., one in which no convection is effective, to a self‐convective system when entering the potential domain, in which the ${\mathrm{NO}}_{3}^{-}\mathrm{RR}$ is superimposed on the gas‐evolving HER. It is the gas‐evolving HER which introduces extra electrolyte stirring to the system affecting the ${\mathrm{NO}}_{3}^{-}\mathrm{RR}$ rates which are governed by nitrate mass transport limitations at high current densities due to increasing nitrate consumption. Although the nitrate concentration had decreased significantly by ≈53% to (46.79 ± 0.39) mmol L^−1^ after 30 min of electrolysis at –0.6 V (Figure 4c), the corresponding partial ammonia current density remained on a high level of *j*_NH3_ = (–230 ± 11) mA cm^−2^ (Figure 4a) which is comparable to the value in the corresponding constant‐charge experiment at –0.6 V, *j*_NH3_ = (–274 ± 14) mA cm^−2^ (Figure 4d), where the reactant consumption is still negligible (Figure 4f). This suggests that the partial current densities for ammonia production can remain high due to an additional HER‐mediated convection, compensating for adverse effects on the ammonia production rate due to reactant depletion/consumption. Qualitatively similar effects are observed for the nitrite reduction reaction (Figure S24 and Table S5, Supporting Information).

More extended electrolysis experiments, carried out at –0.3 V vs RHE where the ${\mathrm{NO}}_{3}^{-}\mathrm{RR}$ is not yet superimposed on the HER, show the expected correlated decline of the total current density (*j*_tot_), of the ammonia partial current density (*j*_NH3_), and of the ammonia Faradaic efficiency (*FE*_NH3_) during electrolysis along with the decrease in nitrate concentration (Figure S25 and Table S6, Supporting Information). In the absence of *vigorous* gas evolution (HER‐mediated “self‐convection”), the limiting current density for the conversion of nitrate to ammonia (j_NH3,lim_) must decrease during electrolysis as the reactant concentration decreases. The replenishment of the electrolyte solution following each 6‐h period of continuous electrolysis resulted in the almost complete recovery of the initially observed ammonia Faradaic efficiency (*FE*_NH3_) close to 100%. This result also demonstrates the robustness of the cobalt‐based catalyst system employed in this study when the catalyst is subjected to various forms of stress.

The observed increase in the transport‐limited current of nitrate reduction (Figure 4a,d), which coincides with the initiation of hydrogen evolution and keeps increasing with rising *j*_HER_, can be attributed to a convective (stirring) effect induced by hydrogen bubbles detaching from the electrode surface. This convective effect arises from multiple factors,^[^ ^15^ ^]^ but the most straightforward model based on the works of Venczel and Ibl^[^ ^15a^ ^]^ rationalizes it by a shortening of the diffusion layer thickness *δ* caused by hydrogen bubbles vacating the surface, thereby allowing fresh electrolyte solution to replenish their previously occupied space. According to this model, the diffusion layer thickness *δ* depends on the *D* diffusion coefficient of the reacting species (for nitrate ions *D* ≈ 1.6 · 10^−5^ cm^2^ s^−1^), the *r* average leaving radius of the bubbles, the *θ* coverage of the surface by the bubbles, and a *ν*_g_ velocity term gained by normalizing the (volumetric) rate of gas evolution to the surface area of the electrode:

(7) $$\delta =\sqrt{\frac{\pi Dr}{6v_{g}}\left(1-\theta \right)}$$

Using the ideal gas law, *v*_g_ can be related to the partial current density of hydrogen evolution

**Figure 5:**
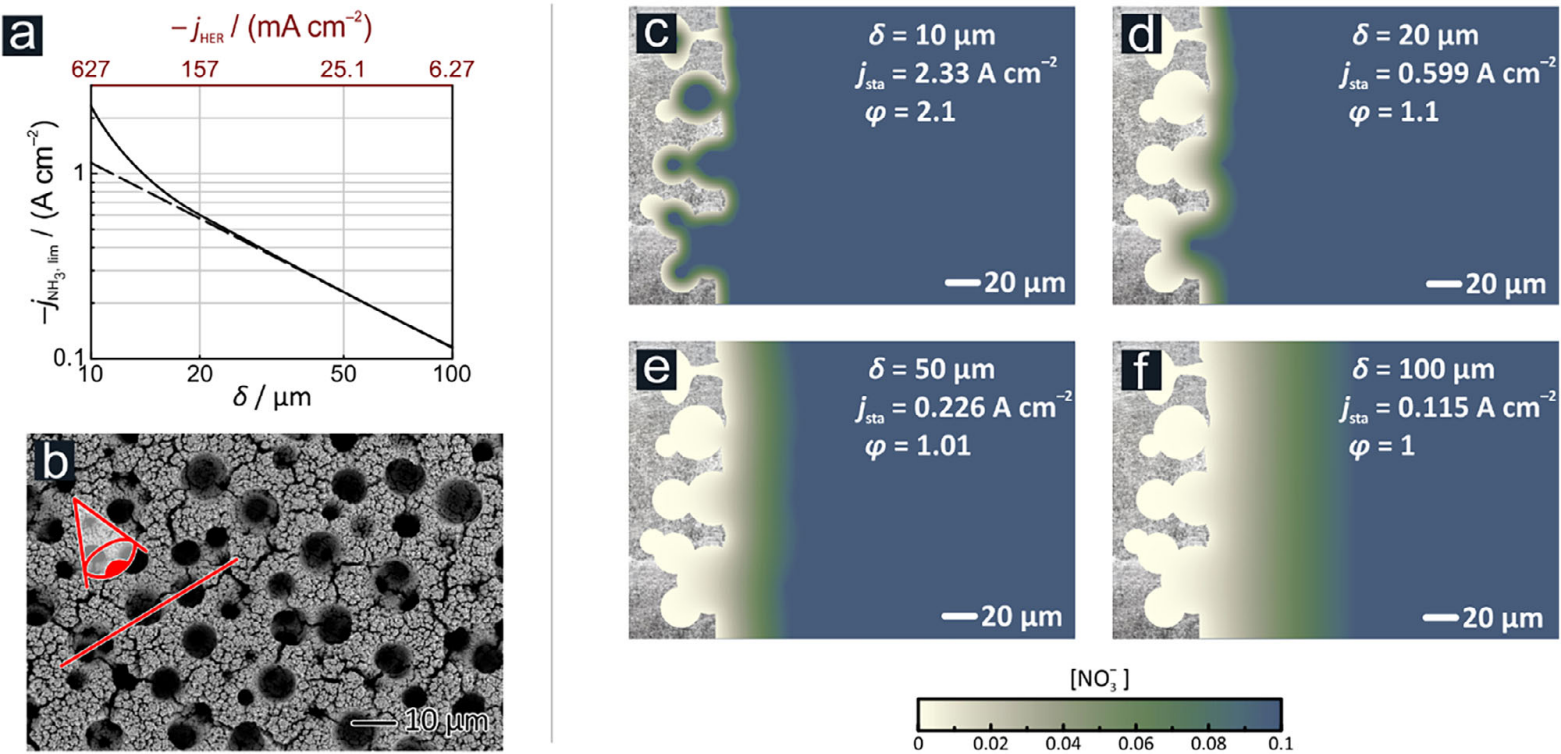
a) Correlation of the limiting partial current density for ammonia production ($-j_{NH_{3},\;\lim}$) to the associated partial current density of parasitic HER (− *j*_HER _) and the corresponding diffusion layer thickness for nitrate (*δ*), calculated using Equations (8) and (9) and approximate bubble parameters listed in the text. b) Representative top‐down SEM image. c–f) Simulated nitrate concentration gradients under stationary conditions depending on the nitrate diffusion layer thickness.

*j*_HER_ as

(8) $$v_{g}=\frac{j_{\mathrm{HER}}RT}{2FP}$$

where *P* is the pressure of hydrogen inside the bubbles of curved surface. While Equations (7) and (8) contain several parameters that are difficult to determine experimentally, it can safely be stated that under realistic conditions (*r* ≈ 100 µm, *θ* ≈ 5%, *P* = 1 bar, *T* = 300 K) hydrogen evolution partial current densities in the range of few hundred mA cm^−2^ can already compress the diffusion layer thickness from a few hundred µm (characteristic to natural convection, Table 1) to ≈10 µm. Even on planar electrodes, this enhanced material transport rate could enable nitrate reduction to reach higher than –1 A cm^−2^ current density. This can be seen by solving the diffusion equation in 1D over a finite domain (see the dashed line in **Figure** **5**a showing the solution). On the metal foam electrodes used in this study, the increase can be even higher (solid curve in Figure 5a) due to the surface structure of the foam (see Figure 5b) that contains openings comparable in size to the diffusion layer thickness. Digital simulations carried out in 2D perpendicular to the foam surface (an assumed in‐depth pore structure along the red line in Figure 5a is used as basis of simulations, results of which are shown in Figure 5c–f) clearly indicate that as the diffusion front is pressed more inside the pores of the foam, the achieved steady‐state current can substantially increase (see Figure S26, Supporting Information for time‐dependent simulations and details of calculation). We introduce the parameter *φ* (see Figure 5c–f) as a measure of how effectively the inner surface of the 3D foam structure is used for the mass transport‐limited nitrate reduction reaction. If, at a given diffusion layer thickness *δ*, the achievable partial current density on a planar electrode would be *j*_planar_, and *j* is the respective current density achieved on a porous electrode at the same diffusion layer thickness, then *φ* is defined as the ratio *j* / *j*_planar_, with both current densities normalized to the geometric surface area. As illustrated in Figure 5f, a *φ* value of 1 indicates a scenario where only the outermost surface of the metal foam (close to its geometric surface area) contributes to the ${\mathrm{NO}}_{3}^{-}\mathrm{RR}$ partial current density. Conversely, a value of *φ* > 1 (Figure 5c) indicates an effective use of the inner surface area of the metal foam. This shows that HER has a dual effect. It accelerates the overall nitrate mass transport through convection contributions and, in the case of 3D foam catalysts, increases the active surface area.

These considerations further underline the importance of designing catalysts surfaces of not only large surface area, but with a roughness that is on the length scale comparable to the achievable diffusion layer thickness. As shown by our simulations, roughness on a smaller scale cannot significantly increase the achievable current density, as pores that are too small (with *R* << *δ*) get depleted already at the start of electrolysis on a sub‐second time scale and cannot be filled with new reactant in the absence of strong convective forces.

### 2.3 Identification of the Catalyst Component Active Toward ${\mathrm{NO}}_{3}^{-}\mathrm{RR}$

**Figure 6:**
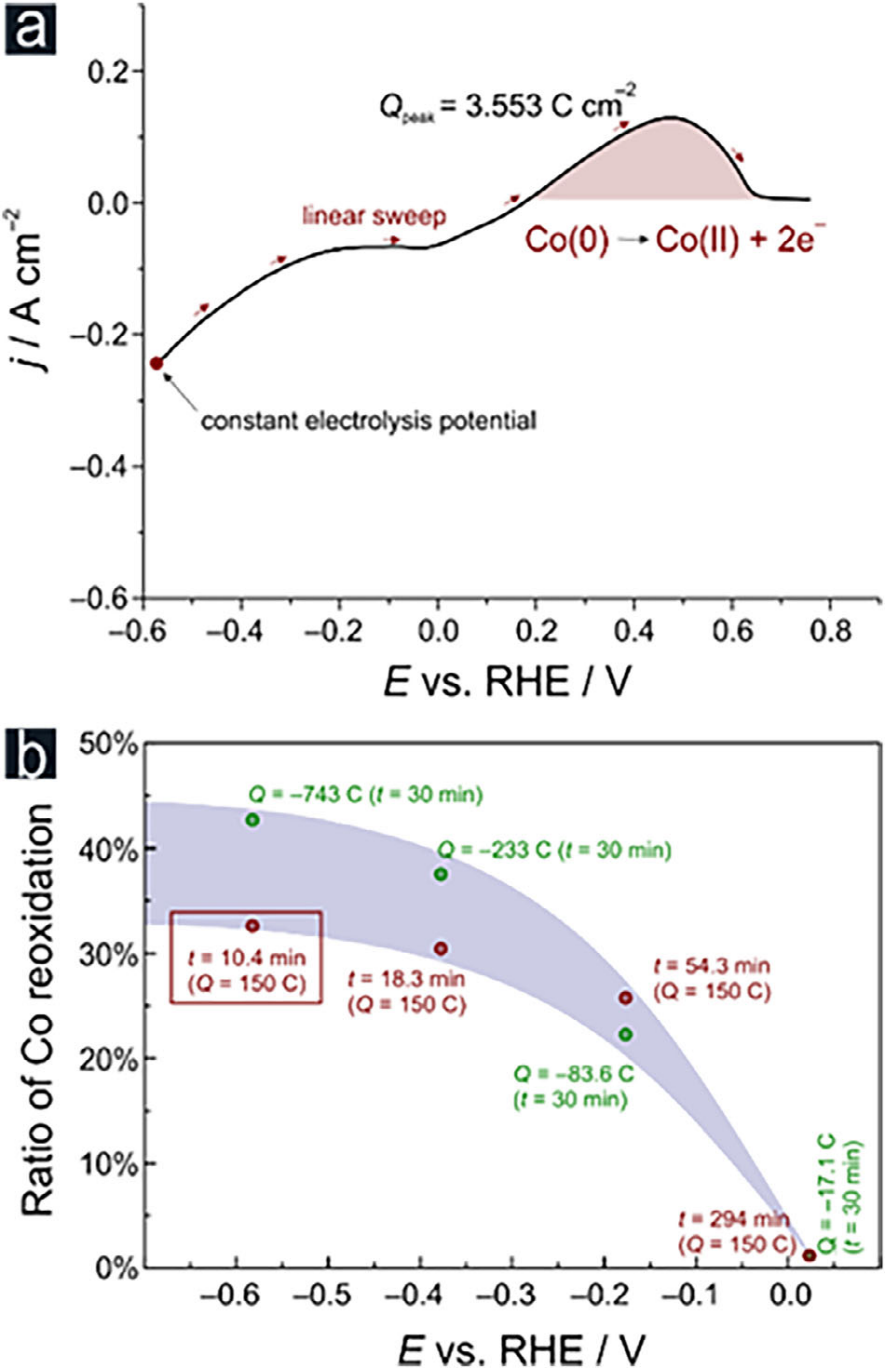
a) Representative linear sweep voltammogram (LSV) recorded at a sweep rate of d*E*/d*t* = 10 mV s^−1^ following a constant charge electrolysis (*Q*_electrolysis_ = –150 C; 10.4 min duration) in 1 mol L^−1^ KOH + 0.1 mol L^−1^ KNO_3_ solution (*p*H 13.7) at *E* = –0.575 V vs RHE using the Co/Co*_x_* O*_y_* H*_z_* foam as the electrocatalyst precursor. The integrated area of the oxidation peak assigned to the Co(0)→Co(II) transition (highlighted red) served as basis for the charge determination. b) Ratio of Co reoxidation derived from LSV analyses (see Figures S27 and S28, Supporting Information). The data point corresponding to the exemplary LSV experiment shown in panel a is highlighted by the red frame in panel b.

The observed increase in ammonia partial current densities, mediated by the self‐convective HER effects, originates from changes in the chemical composition of the active catalyst layer. To study in particular the changing content of metallic Co in the active surface layer, the potentiostatic time‐ and charge‐normalized electrolyses (Figure 4) were further combined with subsequent (backward) linear sweep voltammetry covering the potential range in which the anodic Co(0)/Co(II) transition takes place. A representative LSV following constant‐charge electrolysis of –150 C at –0.6 V vs RHE is presented in **Figure** **6**a. Charges associated with the Co(0)/Co(II) oxidation, derived from the integration of the oxidation peak after background (double layer) correction, were related to the total amount of Co quantified by an ICP‐MS analysis following oxidative catalyst dissolution (Figure 6b; Figures S27 and S28, Supporting Information). Clearly, the Co content in the electrochemically accessible active layer increases with negative‐going electrolysis potentials.

This electrolysis‐related increase in the Co content amounts to ca. 35–45% at –0.6 V vs RHE (Figure 6b). Important to note is that there is no apparent Co‐reoxidation taking place following electrolyses at lowest applied overpotential (0 V vs RHE, Figure 6b), although the ex situ XAS and XRD characterization of the pristine Co/Co*_x_* O*_y_* H*_z_* catalyst precursor clearly proves the presence of a substantial amount of metallic Co present in the as‐prepared Co/Co*_x_* O*_y_* H*_z_* composite (Figure 2; Figures S17 and S18, Supporting Information). This points to the fact that most of the metallic Co present in the pristine Co/Co*_x_* O*_y_* H*_z_* catalyst precursor remains obscured for any electrochemical transformation probed in the voltammetric experiments.

Dedicated catalyst stressing experiments (Figure S29, Supporting Information) indicate that there is no substantial increase/decrease of the catalyst's surface area contributing to the nitrate reduction when the electrode potential is repetitively cycled through the potential range of Co oxidation/reduction.

Ex situ and *operando* XAS measured in transmission mode probe the entire 3D electrode, whereas only the outermost part of the foam material contributes to the electro‐transformations probed in the voltammetric experiments. This analysis points to a metallic interior of the composite foam material which is covered by a thin oxide layer that undergoes potential‐dependent cathodic transformations.

**Figure 7:**
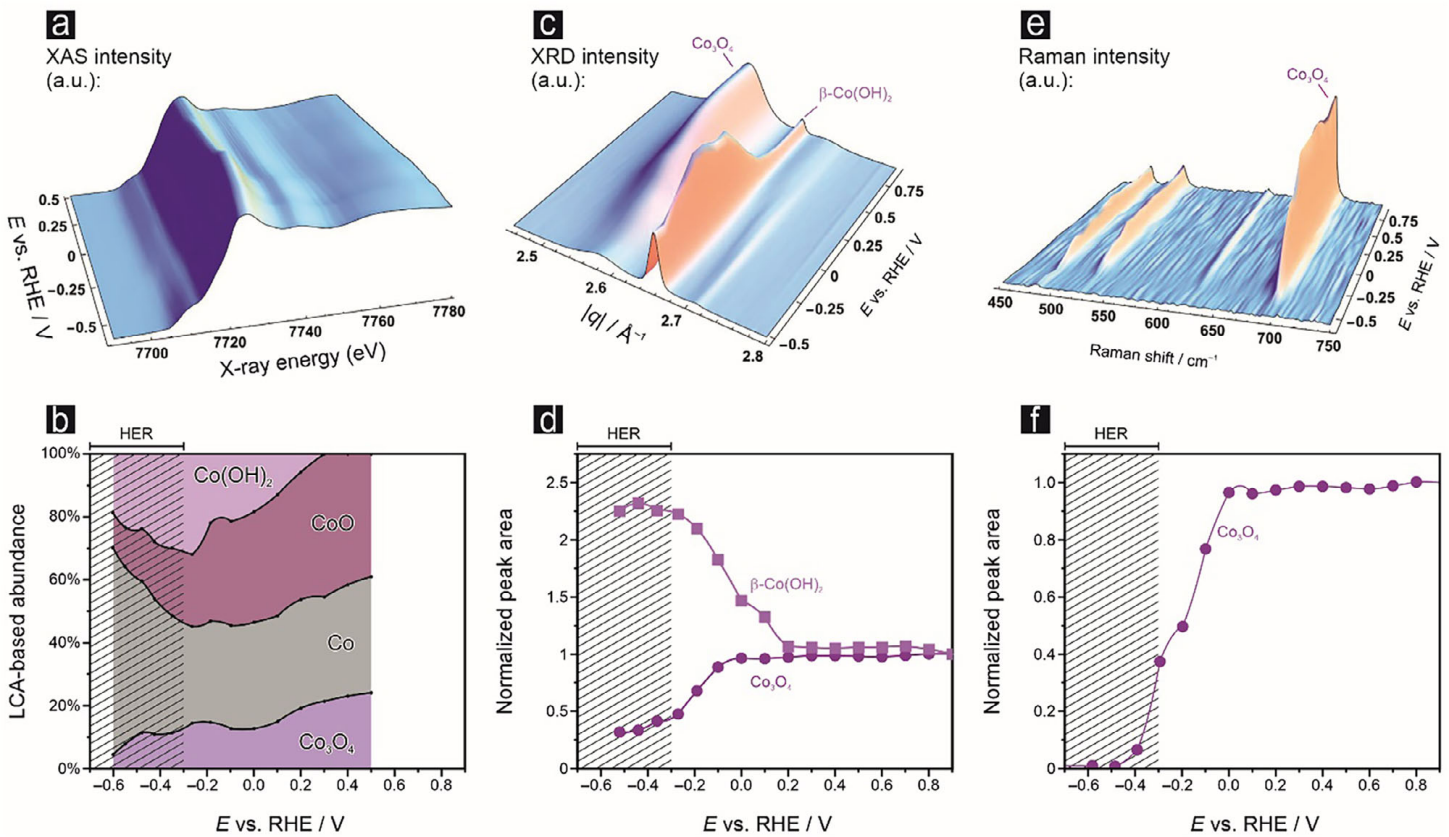
Potential dependent compositional changes of the Co/Co*_x_* O*_y_* H*_z_* foam probed by complementary *operando* techniques. The *operando* experiments were carried out in 1 mol L^−1^ KOH + 0.1 mol L^−1^ KNO_3_ solution (*p*H 13.7). Panel a) shows potential‐dependent Co K‐edge spectra collected in the potential range between +0.5 and –0.6 V vs RHE, yielding the potential‐dependent composition of the probed catalyst foam shown in panel b), determined by a four‐component linear combination analysis. Potential‐dependent *operando* X‐ray diffractograms collected in the range between +0.9 and –0.6 V vs RHE are shown in panel c), with Co_3_O_4_(311) and β‐Co(OH)_2_(101) reflections marked and their peak area plotted in panel d). Panel e) shows potential dependent *operando* Raman spectra, with normalized peak integrals plotted separately in panel f). The presented *operando* data are all *iR*‐corrected; for more detail on the XAS, XRD, and Raman analysis see, respectively, Figures S30–S32 (Supporting Information).

**Figure** **7** summarizes results of the *operando* XAS, XRD, and Raman measurements, which are highly complementary and confirm substantial compositional changes in the 3D bulk of the Co/Co*_x_* O*_y_* H*_z_* composite material. XAS measurements (Figure 7a,b) provide strong evidence for pristine Co_3_O_4_ as the chemical precursor for the reductive Co(OH)_2_ formation. Changes in their LCA weights are anticorrelated up to a potential of ≈–0.2 V vs RHE, while the abundance of Co remains fairly constant in this potential regime. Qiao et al. have recently described a similar catalyst activation process for spinel‐type mixed oxides^[^ ^19c^ ^]^ of CoN, in light of which it is important to note that the observed Co(III)/Co(II) reduction only occurs far below the respective oxidation potential probed in the voltammetry (see Figure 3a). This suggests a significant kinetic stabilization of the Co_3_O_4_ phase (see also the Pourbaix diagram in Figure S15a, Supporting Information). Below –0.3 V vs RHE, the Co content starts to increase substantially at the expense of the oxidized Co(OH)_2_/CoO and Co_3_O_4_ phases, which correlates well with the observed increase in the HER partial current density in the same potential range (see Figures 4a and 7, as well as Figures S23 and S30, Supporting Information). This observation suggests that the increase in HER below –0.3 V vs RHE is directly related to an enrichment of metallic Co in the active surface layer of the foam catalyst. The overall increase in Co content of ≈35–40%, observed by *operando* XAS with respect to the initial conditions after reaching a potential of *E* = –0.6 V vs RHE (Figure 7a), agrees well with the voltammetric analysis shown in Figure 6b, which solely probes transformations occurring in the active surface layer of the catalyst.

An anti‐correlated decrease in the abundance of Co_3_O_4_ and a corresponding increase in the content of crystalline β‐Co(OH)_2_ is further confirmed by the complementary *operando* XRD measurements presented in Figure 7b. Substantial amounts of β‐Co(OH)_2_ forms in the potential regime even before the ${\mathrm{NO}}_{3}^{-}\mathrm{RR}$ onset, indicating that this transformation is purely potential‐dependent and not driven by the ${\mathrm{NO}}_{3}^{-}\mathrm{RR}$ itself. It is of note at this point that the *operando* XRD experiments were, in contrast to the *operando* XAS data, measured in a grazing incidence configuration (incident angle of <2°; beam diameter of ca. 2 µm; foam thickness of ca. 20 µm; beam alignment using the most intense Co_3_O_4_(311) reflection). This explains why the *operando* XRD probes preferentially the outermost section of the foam material and may therefore have stronger relative contributions stemming from the electrochemically accessible active layer than compared to the *operando* XAS measured in transmission mode thereby probing the entire foam structure. Control *operando* XRD experiments carried out in the absence of nitrate in pure 1 mol L^−1^ KOH electrolyte demonstrate a qualitatively similar potential dependence in the relative abundance of the involved Co species as seen for the nitrate containing electrolyte (Figure S31, Supporting Information), thus further supporting our hypothesis that the massive appearance of β‐Co(OH)_2_ is not directly related to ${\mathrm{NO}}_{3}^{-}\mathrm{RR}$. β‐Co(OH)_2hope_ formation can be considered as a surface‐confined precipitation process of hydroxides under alkaline conditions upon release of Co^2+^ from the Co_3_O_4_ precursor following electroreduction of the trivalent Co.

The potential‐dependent consumption of the Co_3_O_4_ precursor is also confirmed by complementary Raman spectroscopy whereas the CoO and Co(OH)_2_ components, although both present in the 3D bulk of the catalyst and its electrochemically accessible surface (Figure 7a), remain invisible in the Raman experiment due to the intense scattering originating from the dominating Co_3_O_4_ spinel surface phase (Figure 7c). Complementary control Raman experiments carried out in pure 1 mol L^−1^ KOH and in nitrite‐containing electrolytes show qualitative similar potential dependencies (Figure S32, Supporting Information). The presence of nitrate and nitrite seems, however, to further suppress Co_3_O_4_ reduction, thus being indicative for a chemisorptive interaction of the nitrate/nitrite with the cobalt oxides. Time‐resolved Raman experiments further confirm the sluggish kinetics in particular of the Co(III)/Co(II) reduction (Figure S33, Supporting Information).

Metal ion precipitation in alkaline media is a common synthetic pathway of synthesizing so‐called layered hydroxide salts (LHSs) with brucite‐like structure, in which, as reviewed by Arizaga et al.,^[^ ^28^ ^]^ certain fraction of the hydroxide groups can even be substituted by water molecules or anions, thus resulting in compounds which are described as β–Co(OH)_2–_ *_x_* A*_x/m_* × *n* H_2_O, where A refers to a co‐precipitated anion and *m* denotes its charge. Nitrate anions are abundant in significant amounts during the surface confined cobalt hydroxide precipitation and may influence the nucleation and growth process of the hydroxide phases. LHSs are well known for the co‐precipitation of nitrate with layered cobalt hydroxides.^[^ ^28^, ^29^ ^]^ It is important to note that the β‐Co(OH)_2_ and derived layered salts are present on the catalyst surface at the ${\mathrm{NO}}_{3}^{-}\mathrm{RR}$ onset (*E* ≥ –0.3 V vs RHE) even before substantial amounts of metallic Co form at *E* < –0.3 V vs RHE, thus suggesting an active role of the β‐Co(OH)_2_ and related LHSs in the nitrate‐to‐ammonia conversion.

**Figure 8:**
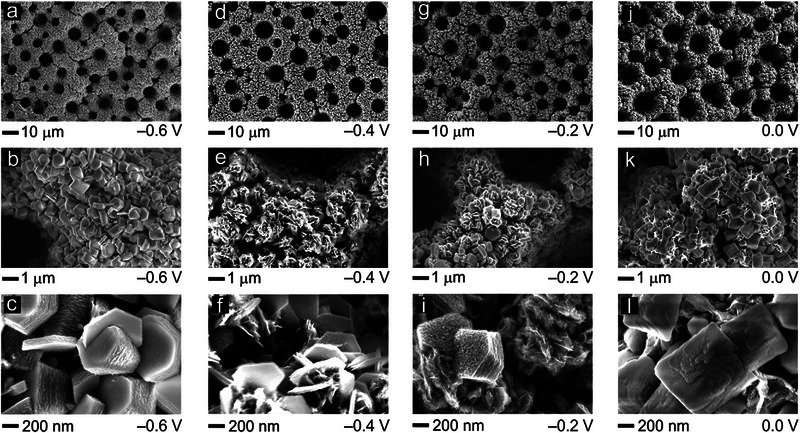
Top‐down SEM characterization at different magnification after potentiostatic (constant‐charge) electrolyses demonstrating the presence of hexagonally shaped Co(OH)_2_ crystallites on the foam surface at all applied potentials. a–c) *E* = –0.6 V vs RHE. d–f) E = –0.4 V vs RHE. g–i) *E* = –0.2 V vs RHE. j–l) *E* = 0.0 V vs RHE.

Complementary post‐electrolysis SEM inspection confirms substantial surface precipitation of highly crystalline β‐Co(OH)_2_, regardless of the applied electrolysis potential (**Figure** **8**; Figure S34, Supporting Information). This observation may also explain the marginal variation of the nitrate‐to‐ammonia efficiencies in the constant‐charge screening experiments (Figure 4e). High‐resolution SEM imaging reveals layered, hexagonally shaped crystallites with dimensions of up to micrometers (Figure S34, Supporting Information). The observed morphological features are fully consistent with the literature describing a hexagonal appearance of brucite‐type crystallites of β‐Co(OH)_2_ and related phases, e.g., synthesized by homogeneous (additive‐assisted) precipitation approaches.^[^ ^30^ ^]^

Identical location SEM imaging (Figure S35, Supporting Information), carried out after given electrolysis times of 5, 15, 30, and 60 min, also indicates that massive β‐Co(OH)_2_ precipitation in the form of µm sized crystallites of layered hydroxides (Figure S34, Supporting Information) occurs only under potential control, but not at OCP. In general, the surface morphology on a larger‐than‐micrometer length scale remains fully unaffected by the electrolysis, whereas the cobalt hydroxide crystallites tend to get perforated, exhibiting surface roughening on a smaller, nanometer length scale (Figure S35, Supporting Information). This also hints to the direct involvement of cobalt hydroxide in ${\mathrm{NO}}_{3}^{-}\mathrm{RR}$ as hypothesized above.

Also note that formed ammonia as the main ${\mathrm{NO}}_{3}^{-}\mathrm{RR}$ product is a strongly complexing ligand that can in principle coordinate and, when present in sufficient amounts, further leach out Co^2+^ from the β‐Co(OH)_2_ crystallites. ICP‐MS control experiments exclude, however, massive cobalt leaching into the electrolyte during extended electrolysis (see Figure S25e, Supporting Information).

A very minor accumulation of cobalt in the electrolyte is observed particularly in an early stage of electrolysis, most likely due to the initial catalyst activation which is based on partial oxide reduction. Such metal ion dissolution upon oxide reduction has been reported by Mayrhofer et al. for numerous systems, mainly for acidic media.^[^ ^31^ ^]^ IL‐SEM control experiments, carried out in nitrate‐free alkaline electrolyte (Figure S36, Supporting Information), also demonstrate the appearance of surface β‐Co(OH)_2_ in agreement with the corresponding *operando* XAS and XRD data (Figures S30 and S31, Supporting Information). The shape of the formed β‐Co(OH)_2_ crystallites is, however, much less well‐defined than in the corresponding nitrate‐containing electrolyte (Figures S34 and S35, Supporting Information) thus proving a pivotal role of the nitrate in the surface faceting when the β‐Co(OH)_2_ and related LHSs are precipitated. Nitrate intercalation into the layered oxides cannot be excluded at this stage and will be addressed in a forthcoming work.

**Figure 9:**
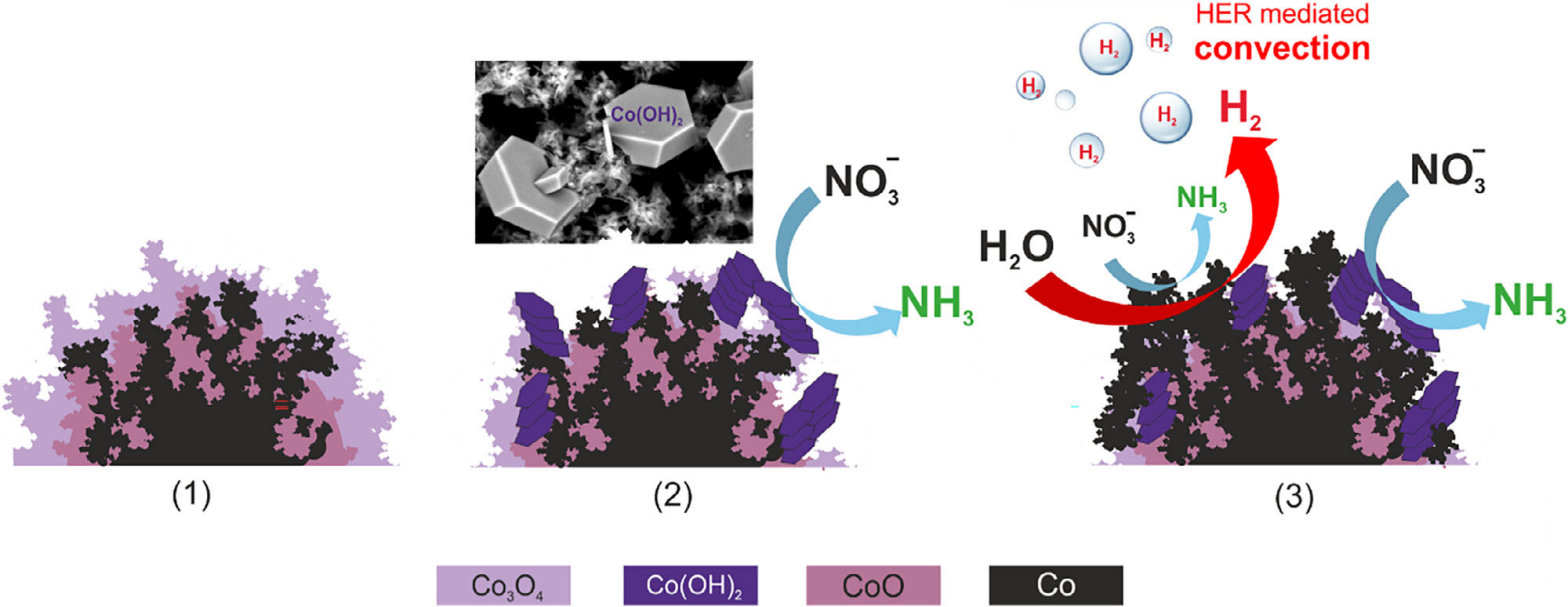
Schematic illustration showing the chemical composition of the Co/Co*_x_* O*_y_* H*_z_* foam: (1) as‐prepared; (2) after β‐Co(OH)_2_ surface precipitation under mild ${\mathrm{NO}}_{3}^{-}\mathrm{RR}$ conditions (e.g., at *E* = 0 V vs RHE); (3) after surface accumulation of metallic Co under competitive HER/ ${\mathrm{NO}}_{3}^{-}\mathrm{RR}$ conditions (*E* < –0.3 V vs RHE).

**Figure** **9** summarizes our findings on the catalyst composition derived from the electrochemical analysis and the combination of complementary *operando* techniques. For the as‐prepared Co/Co*_x_* O*_y_* H*_z_* foam, we assume a layered catalyst material with the Co in its highest oxidation state (Co_3_O_4_ spinel phase) at the outermost surface, while the metallic Co(0) forms the core of the foam, providing sufficient conductivity as a prerequisite for the observed potential‐dependent chemical transformations in the outer active layer. The partial reduction of Co(III) and subsequent release from the Co_3_O_4_ spinel phase gives rise to the surface precipitation of β‐Co(OH)_2_ as the active catalyst phase in the initial stage of ${\mathrm{NO}}_{3}^{-}\mathrm{RR}$. The appearance and accumulation of metallic Co, although it enables HER and thus decreases the *FE* of ${\mathrm{NO}}_{3}^{-}\mathrm{RR}$, does not decrease but rather increases the rate of nitrate reduction by several times. This increase is due to an extra ${\mathrm{NO}}_{3}^{-}\mathrm{RR}$ catalytic pathway that opens up under these cathodic conditions and, most importantly, to additional convection effects originating from the hydrogen bubbles that form and agitate the solution near the electrode, thus enabling sufficient electrolyte replenishment within the porous 3D foam structure.

## 3 Conclusion

In the present study, a novel Co/Co*_x_* O*_y_* H*_z_* model catalyst has been presented, which was prepared by means of the DHBT deposition approach combined with thermal treatment. This catalyst exhibits excellent performance in the electrochemical reduction of nitrate (${\mathrm{NO}}_{3}^{-}\mathrm{RR}$) and nitrite (${\mathrm{NO}}_{2}^{-}\mathrm{RR}$) achieving almost unity faradaic efficiencies for ammonia and current densities in the range of several hundred milliamperes per geometric square centimeter. Even in the case of our foam‐type electrocatalysts, characterized by a 3D architecture of interconnected and open pores and a resulting huge surface area, the achievement of such large currents only becomes possible by significant convection. In the studied system, “self‐convection” is realized by superimposing effervescent hydrogen evolution on the targeted ${\mathrm{NO}}_{3}^{-}\mathrm{RR}$ process. We demonstrated by means of digital simulations that due to this self‐stirring, the diffusion front of ${\mathrm{NO}}_{3}^{-}\mathrm{RR}$ can be effectively pressed into the pore structure of the foam catalyst, allowing the utilization of a large portion of the foam surface. The initiation of the gas‐evolving hydrogen evolution reaction (HER) –typically considered as parasitic to the ${\mathrm{NO}}_{3}^{-}\mathrm{RR}$– provides means for an efficient reactant replenishment in the interior of the 3D foam catalyst, while sacrificial losses of the Faradaic efficiency for ammonia remain moderate. In this present study, we demonstrate that this HER‐mediated boost of the ${\mathrm{NO}}_{3}^{-}\mathrm{RR}$ is, in contrast to the copper reference system, strongly correlated with potential‐dependent compositional changes occurring in the outermost active Co*_x_* O*_y_* H*_z_* oxide/hydroxide layer. Highly complementary *operando* techniques in combination with reverse linear sweep voltammetry after potentiostatic electrolysis provided evidence for β‐Co(OH)_2_, formed by a surface precipitation reaction following reduction of the largely amorphous Co_3_O_4_ catalyst precursor, as the active ${\mathrm{NO}}_{3}^{-}\mathrm{RR}$ catalyst in its initial stage (this means prior to the HER on‐set). ${\mathrm{NO}}_{3}^{-}\mathrm{RR}$ rates experience a substantial boost only after further reduction of the active Co*_x_* O*_y_* H*_z_* layer at more cathodic potentials thereby accumulating metallic Co at the catalyst surface. The effect is a dual one by opening an extra pathway for the ${\;\mathrm{NO}}_{3}^{-}\mathrm{RR}$ (catalytic effect) and accelerating the gas‐evolving HER impacting the system transport conditions (stirring effect). The *operando* analytical techniques applied here confirm a sluggish kinetics for the involved cobalt oxide‐metal transitions taking place at potentials far below the thermodynamic equilibrium potentials which may explain the partially conflicting results and discussions in literature on the chemical nature of Co‐based catalysts for the ${\mathrm{NO}}_{3}^{-}\mathrm{RR}$.

## 4 Experimental Section

### Electrode Fabrication

For the catalyst preparation, 3D Co foams were electrodeposited onto Ni foil supports (MaTeck, 99.7 % metal basis, 0.25 mm thickness, Figure S2, Supporting Information) using the dynamic hydrogen bubble template (DHBT) deposition approach.^[^ ^21^ ^]^ Prior to the electroplating process, 1 × 2.5 cm^2^ cuts of the as‐received Ni sheets were masked with PTFE to obtain a total geometric surface area of 1 cm^2^ (Figure S3, Supporting Information), which was subsequently exposed to the plating bath. Electrolyte solutions were prepared with Milli‐Q water (Millipore, 18.2 MΩ cm, total organic carbon content (TOC) below 5 ppb) and contained 1.5 mol L^−1^ NH_4_Cl (ACS reagent grade, ≥99.5%, Sigma–Aldrich), 0.1 mol L^−1^ CoSO_4_ · 7 H_2_O (ReagentPlus, ≥99%, Sigma Aldrich) as the Co source, and 0.01 mol L^−1^ sodium citrate monohydrate (purum p.a., anhydrous, ≥99.0%, Sigma–Aldrich) as a plating additive. The resulting *p*H of the solution was ≈4.5. It was of note that the use of citrate as an additive had a positive effect on the adhesion of Co foams to the Ni support. In the absence of citrate, the Co foams produced tend to delaminate from the Ni support during electrolysis, particularly under gas‐evolving HER conditions.

The Co‐foaming process was carried out under galvanostatic conditions in a three‐electrode configuration (Figure S4, Supporting Information) using a potentiostat/galvanostat (Methrohm multi Autolab M204 N, Switzerland) equipped with a 10 A booster. The masked Ni support, a Pt foil (MaTeck, Germany, A_geo_ ≈ 25 cm^2^), and a Ag/AgCl/3M KCl electrode (Metrohm, double junction design) served as the working (WE), the counter (CE), and the reference electrode (RE), respectively. The geometric current density (*j*_geo_) was set to –3 A cm^−2^ for 5, 10, 20, 30, 40, 50, and 60 s, resulting in Co foams with varying thickness and pore size distribution (Figures S5 and S7 and Table S1, Supporting Information). Note that the Co foaming process was significantly influenced by the presence and concentration of the citrate additive, which can influence hydrogen bubble break‐off diameter and, consequently, the porosity of the Co foam (Figure S8, Supporting Information).

The mass loading was determined gravimetrically, and for selected samples also by means of ICP‐MS (NexION‐2000, Perkin) analysis, following their oxidative dissolution in 1.5 mL of 30 wt.% HNO_3_ solution that was further diluted with Milli‐Q water to a volume of 10 mL. Aliquots of this latter solution were further diluted by a factor of 10^4^ using 2wt. % HNO_3_ solution for the actual ICP‐MS measurement.

The electrodeposition process was applied to both sides of the Ni foils. Subsequent inspection by means of scanning electron microscopy (SEM) following the metal foaming process revealed no significant differences in the morphology of the Co foams deposited on the front and back sides of the Ni support electrode (Figure S9, Supporting Information). After plating, the Co deposits were thoroughly rinsed and soaked in Milli‐Q water for 1 h for complete removal of residual traces of electrolyte (particularly ${\mathrm{NH}}_{4}^{+}$ ions). Subsequently, the cleaned foams were dried in an Ar gas stream (Carbagas, Switzerland, 99.999 %) and stored in air. In the following, these were denoted as‐deposited Co/Co(OH)_2_@Ni foams, as they were prone to oxidation, forming a thin surface layer of CoO which readily hydrolyzes in humid air and water into Co(OH)_2_ as evidenced by top‐down SEM inspection (Figures S5–S7, Supporting Information) and ex situ Raman spectroscopy (Figure S10, Supporting Information). Note that the extent of this Co(OH)_2_ formation on the foam surface can vary depending on the drying conditions after removing the foam from the plating solution (Figure S11, Supporting Information). For this reason, the Co foams were subjected to a controlled thermal treatment (calcination) step prior to electrolysis to generate a well‐defined oxide layer as a starting point for the electrolytic reaction (see below).

For dedicated *operando* Raman, XAS, and XRD experiments, similar Co foams were fabricated on carbon support electrodes, in the following denoted Co/Co(OH)_2_@C. Prior to the Co electrodeposition, the 0.13 mm thick graphite support electrode (Alfa Aesar, 99.8 %) was activated by thermal treatment at 500 °C in air for 12 h (Nabertherm tube furnace, Germany). As evidenced by control SEM experiments, the obtained Co/Co(OH)_2_@C foams reveal similar morphological characteristics (Figure S12, Supporting Information) as the corresponding Co/Co(OH)_2_@Ni samples used for the electrochemical performance tests (Figures S5–S7, Supporting Information). For further catalyst activation, the as‐deposited Co/Co(OH)_2_@Ni and Co/Co(OH)_2_@C foams were thermally annealed in air at 300 °C for 6 h (Nabertherm tube furnace, Germany) thereby forming metal‐oxide composites, in the following referred to as Co/Co*_x_* O*_y_* H*_z_* @Ni (Figure S7c, Supporting Information) and Co/Co*_x_* O*_y_* H*_z_* @C foams. For the subsequent nitrate electrolysis experiments, these metal‐oxide composite foams served as catalyst precursors.

### Catalyst (Precursor) Characterization

The surface morphology of the as received Ni foils was characterized by means of atomic force microscopy (Nanosurf Flex AFM V2 system, Tap150Al‐G silicon cantilever). Morphological analysis of the as‐deposited Co/Co(OH)_2_ foam and the thermally treated Co/Co*_x_* O*_y_* H*_z_* samples was carried out by means of scanning electron microscopy (SEM) using a Zeiss Gemini 450 instrument equipped with a secondary electron and a backscattered electron detector. Accelerating voltages (electron currents) of 5.0 kV (100 pA) were applied as default settings for the secondary and backscattered electron detection mode. AZtec 4.2 software (Oxford Instruments) was used to acquire and process energy dispersive X‐ray (EDX, 20 kV (1.5 nA) point spectra and 2D elemental mappings. Raman spectroscopic measurements were performed using a LabRAM HR800 confocal microscope (Horiba Jobin Yvon) featuring a large working distance objective lens (50 times magnification, 8 mm focal length) with a numerical aperture of 0.1 to focus a diode‐pumped solid‐state laser beam (excitation wavelength 532 nm) on the sample. Raman signals were collected in backscattering geometry using a custom‐made spectro‐electrochemical flow‐cell which was described elsewhere.^[^ ^32^ ^]^ To prevent undesired beam damages, an optimized laser power of 3 and 0.12 mW was applied for the *operando* and ex situ Raman investigation, respectively. All Raman experiments were carried out in three‐electrode configuration with the Co/Co*_x_* O*_y_* H*_z_* @C foams serving as the working electrode (WE), a Au wire was employed as the counter electrode (CE), and a Ag/AgCl/3M KCl (MW‐2030, BASi, US) served as the reference electrode (RE). During Raman measurements, electrolysis potentials were controlled by an Autolab 3 potentiostat (Metrohm, Switzerland) operated by GPES 4.0 software. Cell resistances were measured prior to the *operando* Raman experiments by electrochemical impedance spectroscopy (EIS) using an Autolab 302N instrument (Metrohm, Switzerland) equipped with an FRA module. The resting and measurement times at each applied electrolysis potential before and during the Raman measurements were 1 and 3 min, respectively. During the measurement process, a total of ten individual Raman spectra were acquired at each applied potential and subsequently averaged.

XRD experiments were performed at the ID31 high‐energy beamline at the European Synchrotron Radiation Facility (ESRF, Grenoble, France). The 69 keV monochromatic X‐ray beam was focused on the sample position to the spot size of 5 × 20 µm2 (vertical × horizontal). 2D XRD patterns were collected with a Dectris Pilatus 2 M CdTe detector. Ex situ XRD measurements were performed in transmission mode.

A custom‐made PEEK electrochemical flow‐cell contained a Pt wire as CE, a leakless Ag/AgCl/3M KCl RE (eDAQ) and the sample WE (Co/Co*_x_* O*_y_* H*_z_* @C foam) which was electrically connected through a Au wire. All Co/Co*_x_* O*_y_* H*_z_* @C samples were first measured at the open circuit potential (OCP) followed by a negative‐going potential step sequence with an increment of 100 mV, covering the potential range from –0.1 to –1.6 V vs Ag/AgCl_3M_. The resting time at each applied electrolysis potential prior (for equilibrating purposes) and during the XRD measurement was 1 and 3 min, respectively. These *operando* XRD experiments were carried out with an incidence angle of <2° (beam diameter of ca. 2 µm; foam thickness of ca. 20 µm; beam alignment using the most intense Co_3_O_4_(311) reflection). This implies that the *operando* XRD experiments preferentially probed the outermost part of the foam structure exposed to the electrolyte.

The raw 2D diffraction patterns were radially integrated to the final 1D patterns using the pyFAI package.^[^ ^33^ ^]^ A selected number of 1D patterns were subjected to further analysis by integrating their potential‐dependent peak intensities and fitting a Gaussian function to determine the full‐width at half‐maximum (FWHM). *Operando* XRD measurements were performed using a beam energy of 69 keV (λ = 0.179 Å), while the ex situ measurements were conducted at 75 keV (λ = 0.165 Å).

XAS data were collected at the ROCK beamline of the French national synchrotron facility SOLEIL (Saint‐Aubin, France). The incident beam (500 mA current) was collimated with a bending magnet (*E*c = 14.5 keV) and a toroidal Si mirror with 50 nm Ir coating and monochromatized with a Si(111) monochromator. Two mirrors for harmonic rejection (1st flat, 2nd bendable) were tilted at 3 mrad with B4C stripes. The beam was attenuated using a 250 CVD attenuator. XAS measurements were performed in transmission mode at the Co K edge. All ionization chambers were filled with pure nitrogen. The length of the ionization chamber for the incident X‐ray intensity (I_0_) was 188 mm. The X‐ray intensity transmitted through the sample and reference (*I*_1_ and *I*_ref_), respectively, was measured using ionization chambers with a length of 328 mm. The Co/Co*_x_* O*_y_* H*_z_* @C samples were placed into a custom‐made PEEK spectro‐electrochemical flow‐cell, as described by Binniger et al.^[^ ^34^ ^]^ All XAS measurements were performed in a three‐electrode configuration with the Co/Co*_x_* O*_y_* H*_z_* @C serving as the WE, a graphite foil (Alfa Aesar, 99.8 %) and a leakless Ag/AgCl (eDAQ) acting as the CE and RE, respectively. Prior to use, the graphite foil was activated by thermal treatment at 500 °C for 12 h in air (Nabertherm tube furnace, Germany). A peristaltic pump was used to achieve an electrolyte flow rate of 1 mL min^−1^. XAS data were processed using the DEMETER software package.^[^ ^35^ ^]^ The amplitude reduction factor was estimated by an EXAFS fit of the Co reference foil (*S*_0_2 = 0.83). Fit ranges used where R = 1.2–3 Å, *k* = 3–12 Å^−1^, and fitted with a *k*‐weighting of *k* ^3^. Commercially available standards were used for the reference measurements (Co_3_O_4_: nano‐powder, <50 nm particle size (SEM), 99.5% trace metals basis, Sigma–Aldrich; CoO: Technical grade, 95%, Sigma–Aldrich; Co(OH)_2_: nano‐powder, <50 nm average particle size, ≥99.5% trace metals basis, Sigma–Aldrich).

### Catalyst Performance Testing

Voltammetric and potentiostatic electrolysis experiments were performed in a three‐electrode configuration using a divided H‐type cell (Figure S13, Supporting Information) with the Co/Co*_x_* O*_y_* H*_z_* @Ni foam, a Pt foil, and a Ag/AgCl/3M KCl (Pine research) serving as the WE, the CE, and the RE, respectively. Catholyte and anolyte compartments were separated from each other by an anion exchange membrane (Sustainion, X37‐50 RT) and filled with 15 mL of electrolyte solution. Electrolyte solutions were prepared with Milli‐Q water and contained 1 mol L^−1^ KOH (reagent grade, 90%, Sigma–Aldrich) as a supporting electrolyte and extra 0.1 mol L^−1^ KNO_3_ (ACS reagent, ≥99.0%, Sigma–Aldrich) or 0.1 mol L^−1^ KNO_2_ (ACS reagent, ≥96.0%, Sigma–Aldrich) as reactants for the respective nitrate/nitrite electrolysis. Prior to the experiment, the catholyte was degassed for 30 min with Ar gas (99.999%, Carbagas, Switzerland) to remove oxygen. For each voltammetric/potentiostatic electrolysis experiment, a freshly prepared Co/Co*_x_* O*_y_* H*_z_* @Ni foam was used. Potential control was achieved through an Autolab 302N potentiostat/galvanostat (Metrohm, Switzerland) operated by the NOVA 2.1 software. Prior to the electrolysis, cell resistances were measured using the current interrupt method. Unless otherwise stated, the experimental protocol comprised three replicates of potentiostatic electrolysis experiments for each electrolysis potential covering a range from –1.0 to –1.6 V vs Ag/AgCl/3M KCl.

Unless otherwise stated, all reported potentials were *iR*‐compensated (80%) or corrected (e.g., for the *operando* XAS/Raman/XRD experiments), and converted to the RHE scale according to

(9) $$E_{\mathrm{RHE}}=E_{\mathrm{Ag}/\mathrm{AgCl}/3M\;\mathrm{KCl}}+0.210\;V+0.0591\;V\cdot pH$$

Given a solution *p*H of 13.7, the potential shift between the Ag/AgCl/3M KCl and the RHE scale amounts to ≈1.02 V.

### Product Quantification

Ammonia detection and quantification were performed using the indophenol blue method^[^ ^36^ ^]^ (Figure S14, Supporting Information). This analytical approach had been well‐established in the literature and cross‐validated for ${\mathrm{NO}}_{3}^{-}\mathrm{RR}$ applications by complementary analysis methods, e.g., by NMR techniques.^[^ ^36^ ^]^ For quantification purposes,^[^ ^37^ ^]^ catholyte aliquots (25–200 µL) were collected at particular electrolysis times, diluted with Milli‐Q water (1975–1800 µL) and subsequently mixed with 2 mL of 1 mol L^−1^ KOH solution containing 5 wt.% of salicylic acid (ACS reagent, ≥99.0%, Sigma–Aldrich), 5 wt.% of sodium citrate monobasic (purum p.a., anhydrous, ≥99.0%, Sigma–Aldrich), 0.05 M NaOCl (reagent grade, available chlorine 10–15%, Sigma–Aldrich) solution, and 200 µL of an aqueous solution containing 1 wt.% sodium nitroferricyanide(III) dihydrate (ACS reagent, ≥99%, Sigma–Aldrich). Following a reaction time of 1.5 h, UV–vis absorption spectra were recorded in the spectral range from 450 to 900 nm using a Cary 60 UV–vis spectrophotometer. The characteristic absorption maximum of the indophenol derivative was observed at a wavelength of λ = 677 nm.^[^ ^38^ ^]^ Indophenol (ammonia) quantification was based on calibration curves derived from reference solutions in the concentration range from 0.01 to 0.50 mm, prepared from a 100 mm analytical standard solution (Sigma Aldrich, analytical standard). Representative calibration measurements are presented in Figure S14 (Supporting Information).

The Faradaic efficiency of ammonia production (*FE*_NH3_) was determined as the ratio of the charge consumed for NH_3_ production and the total charge (*Q*_tot_) passed through the cell during electrolysis according to:

(10) $$FE_{NH_{3}}=\frac{n\;\cdot \;F\;\cdot c_{NH_{3}}\cdot \;V}{Q_{\mathrm{tot}}}\cdot 100\%$$

The total charge *Q*_tot_ (in C) was determined through integration of the respective electrolysis current vs electrolysis time traces. *F* refers to the Faraday constant (96485 C mol^−1^), $c_{NH_{3}}$ denotes the spectroscopically determined NH_3_ concentration (in mol L^−1^), *V* represents the volume of the catholyte (*V* = 0.015 L), and *n* refers to the number of transferred electrons depending on the reaction considered (*n* = 8 for nitrate‐to‐ammonia and *n* = 6 for nitrite‐to‐ammonia reduction).

The quantification of ${\mathrm{NO}}_{3}^{-}$ (${\mathrm{NO}}_{3}^{-}\mathrm{RR}$ reactant) and ${\mathrm{NO}}_{2}^{-}$ (possible ${\mathrm{NO}}_{3}^{-}\mathrm{RR}$ by‐product) was performed by ion exchange chromatography (IC, Figure S15, Supporting Information) using a Metrohm 940 Professional IC Vario instrument, which was operated by MagIC Net 3.3 software. Catholyte solutions were diluted 20 or 200 times in Milli‐Q water prior to IC analysis.

### Simulations of Nitrate Transport Phenomena During ${\mathrm{NO}}_{3}^{-}\mathrm{RR}$

Simulations carried out in this paper utilize a 2D equidistant mesh with an irregular interface that mimics possible real‐life electrode surfaces of a foamy structure, as hinted by SEM micrographs of the employed Co foams. The purpose of the simulation is two‐fold; that is, the aim was to determine (nitrate) concentration changes as a function of space and time within the diffusion layer (the thickness of which is thought to be fixed by the stirring effect of hydrogen production), and the resulting nitrate current density was also calculated. The latter was a transient signal that in time reaches a limiting current value pertaining to stationary finite diffusion. In the simulations, Dirichlet boundary conditions were used for the interface (where the concentration of the reacting species was fixed at 0) and for a bulk layer positioned at a known distance δ from the surface, where a fixed concentration was assumed. An explicit scheme using a 2D, mutli‐point weighted stencil was used for the simulations, as detailed in the Supporting Information.

### Statistical Analysis

The error bars shown in figures were determined based on, unless otherwise stated, three independent measurements by estimating the expected value through calculating the arithmetic mean, and the standard error (SE) by dividing the standard deviation of measurements with the square root of the sample size. Numerical data (individual measurements, as well as average ± SE intervals) are shown in Tables S3–S5 (Supporting Information).

## Conflict of Interest

The authors declare no conflict of interest.

## Supporting information



Supporting Information

Supplemental Video 1

## Data Availability

Data Availability The raw data for this paper are made fully accessible to the public via Zenodo https://doi.org/10.5281/zenodo.15106959, along with the publication of this manuscript.
