# Supplementary material for: Transport Matters: The Critical Role of the Hydrogen Evolution Reaction (HER) in Accelerating Electrochemical Nitrate to Ammonia Conversion
Source: Adv Sci (Weinh). 2025 Sep 23;12(44):e06733. doi: 10.1002/advs.202506733 (PMC12667537; doi:10.1002/advs.202506733)
Supplement: Supplementary file 1 — Supporting Information [file ADVS-12-e06733-s001.docx]

**Supporting Information**

***to the paper***

**Transport Matters: The Critical Role of the Hydrogen Evolution Reaction (HER) in Accelerating Electrochemical Nitrate to Ammonia Conversion**

Nandu Ashtaman-Pillai Syamaladevi,^1,2^ Abhijit Dutta,^*1,2^ Alain Rieder,^1,2^ Xin Yu,^1,2^ Hridya Nedumkulam,^3^ Jakub Drnec,^3^ Zsolt Szakály,^4^ Soma Vesztergom,^4^ Rebecca Katharina Pittkowski,^5^ and Peter Broekmann^*1,2^

^1^ Department of Chemistry, Biochemistry and Pharmaceutical Sciences, University of Bern, Freiestrasse 3, 3012 Bern, Switzerland

^2^ NCCR Catalysis, Switzerland

^3^ European Synchrotron Radiation Facility (ESRF), 71 Avenue des Martyrs, 38000 Grenoble, France

^4^ MTA–ELTE Momentum Interfacial Electrochemistry Research Group, Eötvös Loránd University, Pázmány Péter Sétány 1/A, 1117 Budapest, Hungary

^5^ Center for High Entropy Alloy Catalysis, Department of Chemistry, University of Copenhagen, Universitetsparken 5, 2100 Copenhagen, Denmark

*Corresponding authors: [abhijit.dutta@unibe.ch](mailto:abhijit.dutta@unibe.ch); [peter.broekmann@unibe.ch](mailto:peter.broekmann@unibe.ch)

**Table of Contents:**

[**Figure S1**](#FigureS1)**:** The effect of diffusion layer thickness on the achievable limiting current density of nitrate-to-ammonia reduction p. 3

[**Figure S2**](#FigureS2)**:** Characterization of the Ni support (photo and AFM) p. 3

[**Figure S3**](#FigureS3)**:** Photographs of foam preparation stages p. 4

[**Figure S4**](#FigureS4)**:** Photograph of the foam deposition cell p. 4

[**Figure S5**](#FigureS5)**:** SEM inspection of deposited Co foams, overview p. 5

[**Figure S6**](#FigureS6)**:** SEM inspection of deposited Co foams, shorter deposition times p. 6

[**Figure S7**](#FigureS7)**:** SEM inspection of deposited Co foams, longer deposition times,
effect of calcination p. 7

[**Figure S8**](#FigureS8)**:** SEM inspection of foams, effect of citrate additive p. 8

[**Figure S9**](#FigureS9)**:** SEM inspection of foams, effect of substrate orientation p. 8

[**Figure S10**](#FigureS10)**:** Ex-situ Raman characterization of deposited foams p. 9

[**Figure S11**](#FigureS11)**:** SEM inspection of foams, effect of drying conditions p. 9

[**Figure S12**](#FigureS12)**:** SEM inspection of foams, substrate effects p. 10

[**Figure S13**](#FigureS13)**:** Photograph of the H-type electrolysis cell p. 10

[**Figure S14**](#FigureS14)**:** Overview of the applied analytic method for NH₃ quantification p. 11

[**Figure S15**](#FigureS16)**:** Chromatograms and calibration plots for nitrate and nitrite quantification p. 12

[**Figure S16**](#FigureS16)**:** EDX mapping and point analysis results obtained from calcinated foam samples p. 13

[**Figure S17**](#FigureS17)**:** Pre-electrolysis XAS investigation of the Co/Co*_x_*O*_y_*H*_z_*@C foam p. 14

[**Figure S18**](#FigureS18)**:** XAS-based chemical composition of the Co/Co*_x_*O*_y_*H*_z_*@C foam
before electrolysis p. 15

[**Figure S19**](#FigureS19)**:** Pourbaix diagrams and related thermodynamic data of Co and N species p. 16

[**Figure S20**](#FigureS20)**:** Cyclic voltammetry of the Co/Co*_x_*O*_y_*H*_z_*@Ni foams used for nitrate reduction,
the effect of negative vertex on hysteresis p. 17

[**Figure S21**](#FigureS21)**:** Experiments screening the efficiency of nitrate-to-ammonia reduction on Co/Co*_x_*O*_y_*H*_z_*@Ni foams prepared using different deposition times p. 18

[**Figure S22**](#FigureS22)**:** Details of the current transients, the integration of which resulted in charges
plotted in Figure 4 of the main text p. 19

[**Figure S23**](#FigureS23)**:** Photographs showing H_2_ evolution concurrent with nitrate reduction
at different applied potentials p. 20

[**Figure S24**](#FigureS24)**:** Overview of electrocatalytic performance towards nitrite electroreduction p. 20

[**Figure S25**](#FigureS25)**:** Results of long-term (discontinuous 24-hours) electrolysis p. 21

[**Figure S26**](#FigureS26)**:** Simulation-based animation of the depletion of diffusion
layers of different thickness p. 22

[**Figure S27**](#FigureS27)**:** Linear sweep voltammograms following constant time electrolyses p. 23

[**Figure S28**](#FigureS28)**:** Linear sweep voltammograms following constant charge electrolyses p. 24

[**Figure S29**](#FigureS29)**:** Linear sweep voltammograms following progressively
extended electrolysis times p. 25

[**Figure S30**](#FigureS30)**:** Details of potential-dependent operando XAS measurements, in parts used for the creation of Figure 7.a and 7.b of the main text p. 26

[**Figure S31**](#FigureS31)**:** Details of potential-dependent operando XRD measurements, in parts used for the creation of Figure 7.c and 7.d of the main text p. 27

[**Figure S32**](#FigureS32)**:** Details of potential-dependent operando Raman measurements, in parts used for the creation of Figure 7.e and 7.f of the main text p. 28

[**Figure S33**](#FigureS33)**:** Details of time-dependent operando Raman measurements p. 28

[**Figure S34**](#FigureS34)**:** Top-down SEM images measured after electrolysis, showing the formation of hexagonally shaped β-Co(OH)_2_ particles p. 29

[**Figure S35**](#FigureS35)**:** Identical location SEM monitoring of the catalyst surface during electrolyses lasting different times: nitrate-containing case p. 30

[**Figure S36**](#FigureS36)**:** Identical location SEM monitoring of the catalyst surface during electrolyses lasting different times: nitrate-free case p. 31

[**Table S1**](#TableS1)**:** Foam thickness, pore diameter, and mass loading values
corresponding to Figure S5 p. 31

[**Table S2**](#TableS2)**:** Calculated XRD patterns p. 32

[**Table S3**](#TableS3)**:** Numerical data for Figure S21 p. 35

[**Table S4**](#TableS4)**:** Numerical data for Figure 4 of the main text p. 36

[**Table S5**](#TableS5)**:** Numerical data for Figure S24 p. 39

[**Table S6**](#TableS6)**:** Numerical data for Figure S25 p. 42


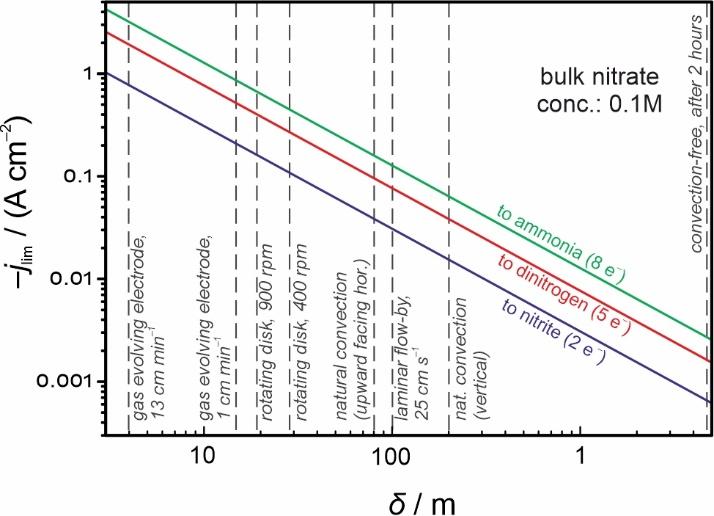


**Figure S1.** Limiting current densities of nitrate reduction yielding three different products are plotted as a function of the diffusion layer thickness. Some diffusion layer thickness values, created by typical hydrodynamic scenarios, are marked by the vertical lines; see Table 1 in the main manuscript for numerical data.


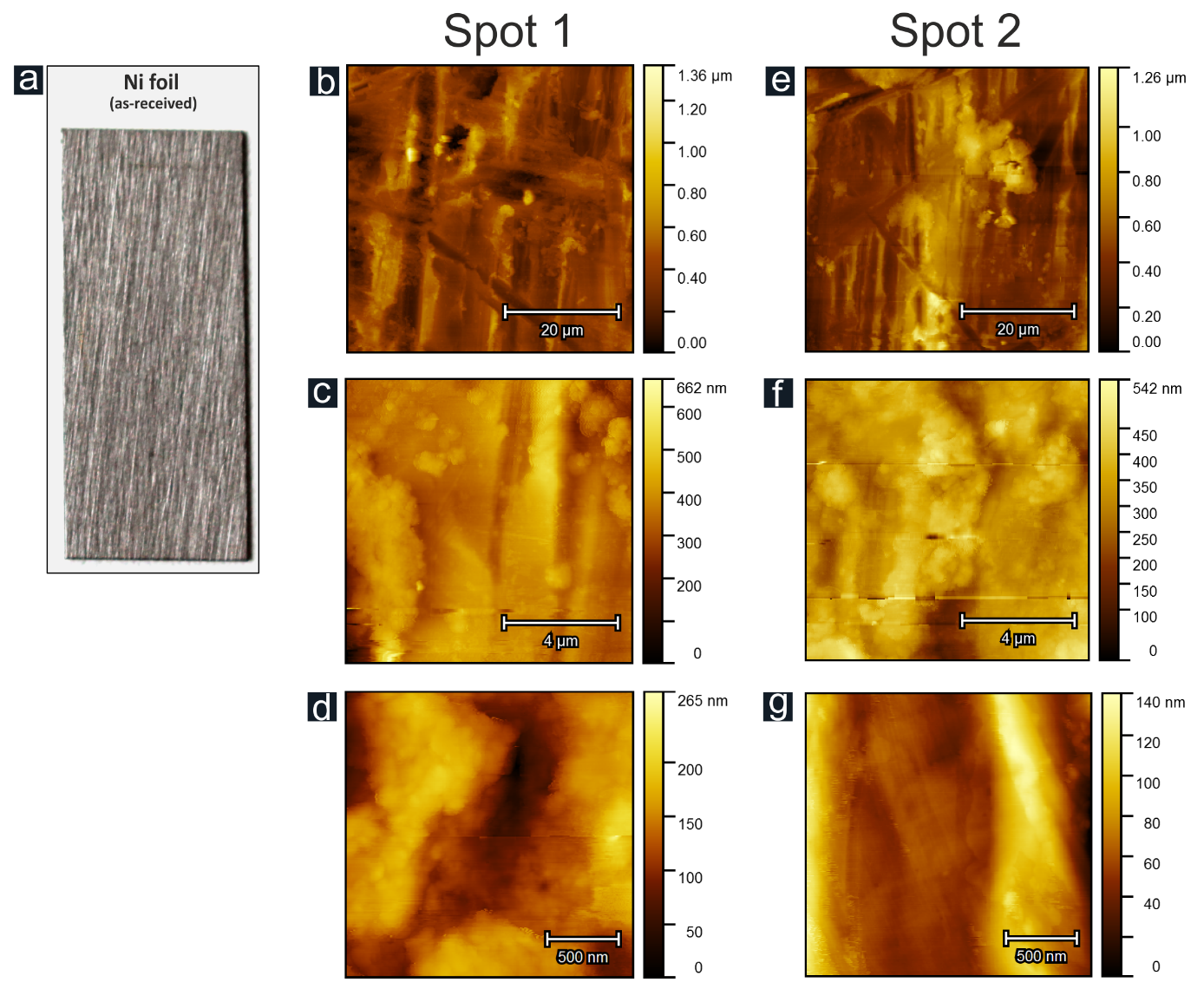


**Figure S2.** a) Photograph of the as-received Ni foil (1 × 2.5 cm^2^ cut) serving as the support for the Co foam electrodeposition. b) – d) AFM images of the as-received Ni support (spot 1). e) – g) AFM images of the as-received Ni support (spot 2). The AFM characterization shows a surface roughness on the µm length scale originating from the foil fabrication (roll out process).


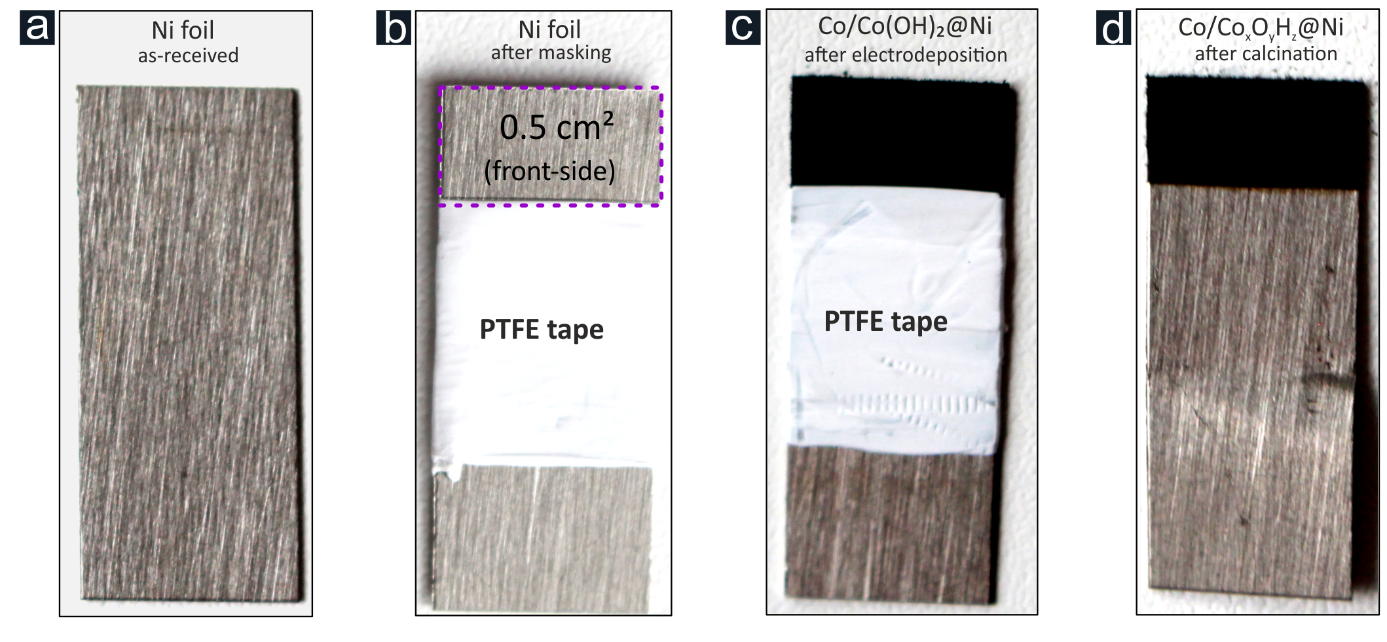


**Figure S3.** Photographs of the Ni support electrode (1 × 2.5 cm^2^ cut) as-received (panel a), after masking with insulating PTFE tape (panel b), after Co foam electrodeposition (panel c), and after thermal treatment (calcination) of the Co foam at 300°C for 6 hours (panel d).


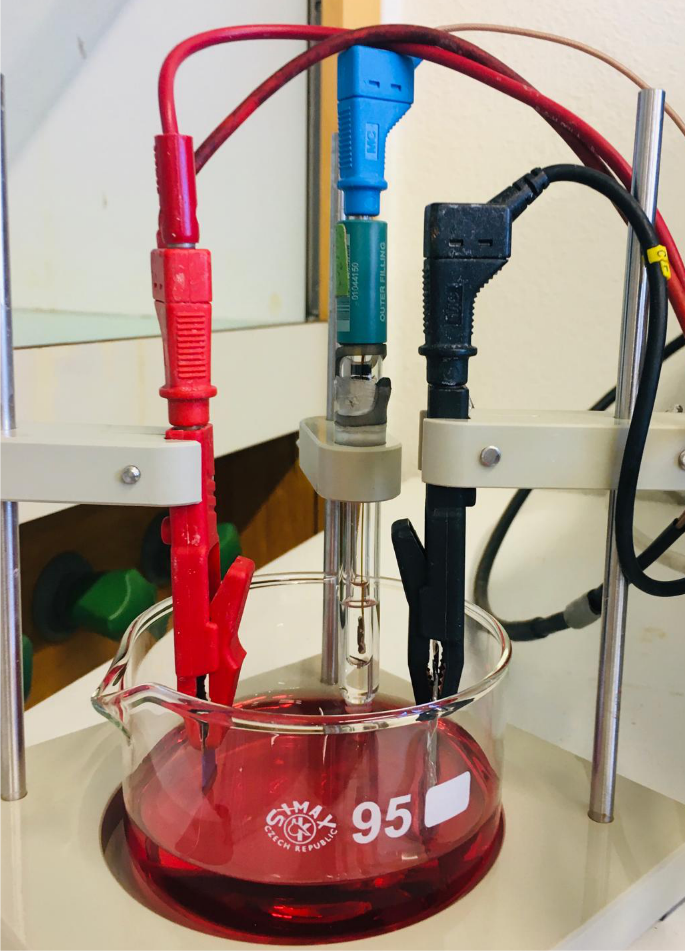


**Figure S4.** Photograph of the plating cell utilized for the deposition of the Co foams. The glass beaker was filled with 100 mL of plating solution containing 1.5 mol L^–1^ NH_4_Cl (ACS reagent grade, ≥ 99.5%, Sigma-Aldrich), 0.1 mol L^–1^ CoSO_4_ · 7 H_2_O (ReagentPlus®, ≥ 99%, Sigma Aldrich) as the Co source, and 0.01 mol L^–1^ sodium citrate monohydrate (purum p.a., anhydrous, ≥ 99.0%, Sigma-Aldrich) as a plating additive. It is important to note that the relative positions and distances of the WE (Ni foil), the CE (Pt foil), and the RE (Ag/AgCl3M electrode) were fixed in the same way for all the deposition processes. The WE and CE electrodes were placed face to face, 5 cm apart.


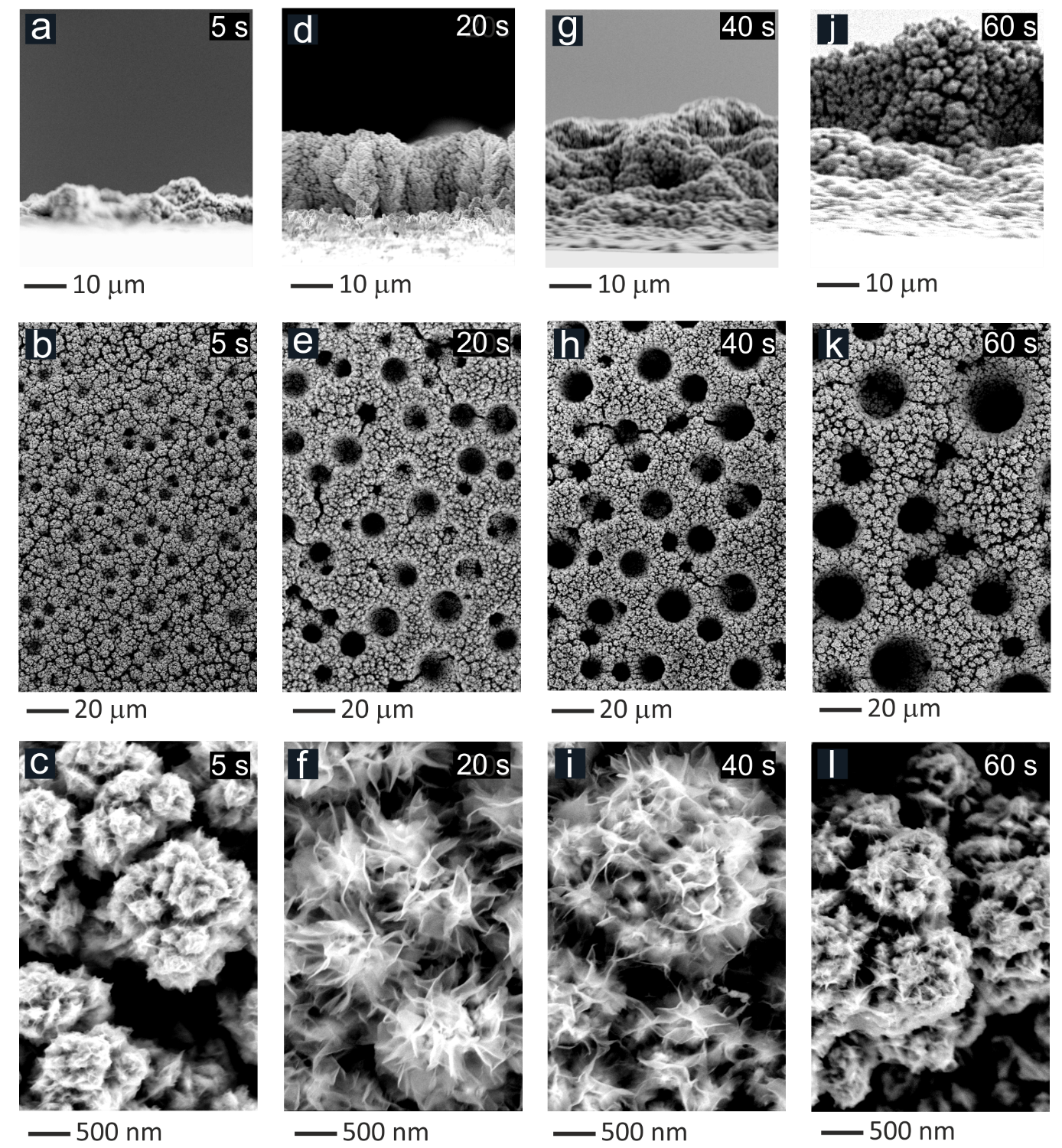


**Figure S5.** Cross-sectional and top-down SEM inspection of the electrodeposited Co/Co(OH)_2_@Ni foam samples following electrodeposition for 5 s (panel a – c), 20 s (panel d – f), 40 s (panel g – i), and 60 s (panel j – l). (See Table S1 for further information on foam thicknesses, surface pore sizes, and mass loadings.)


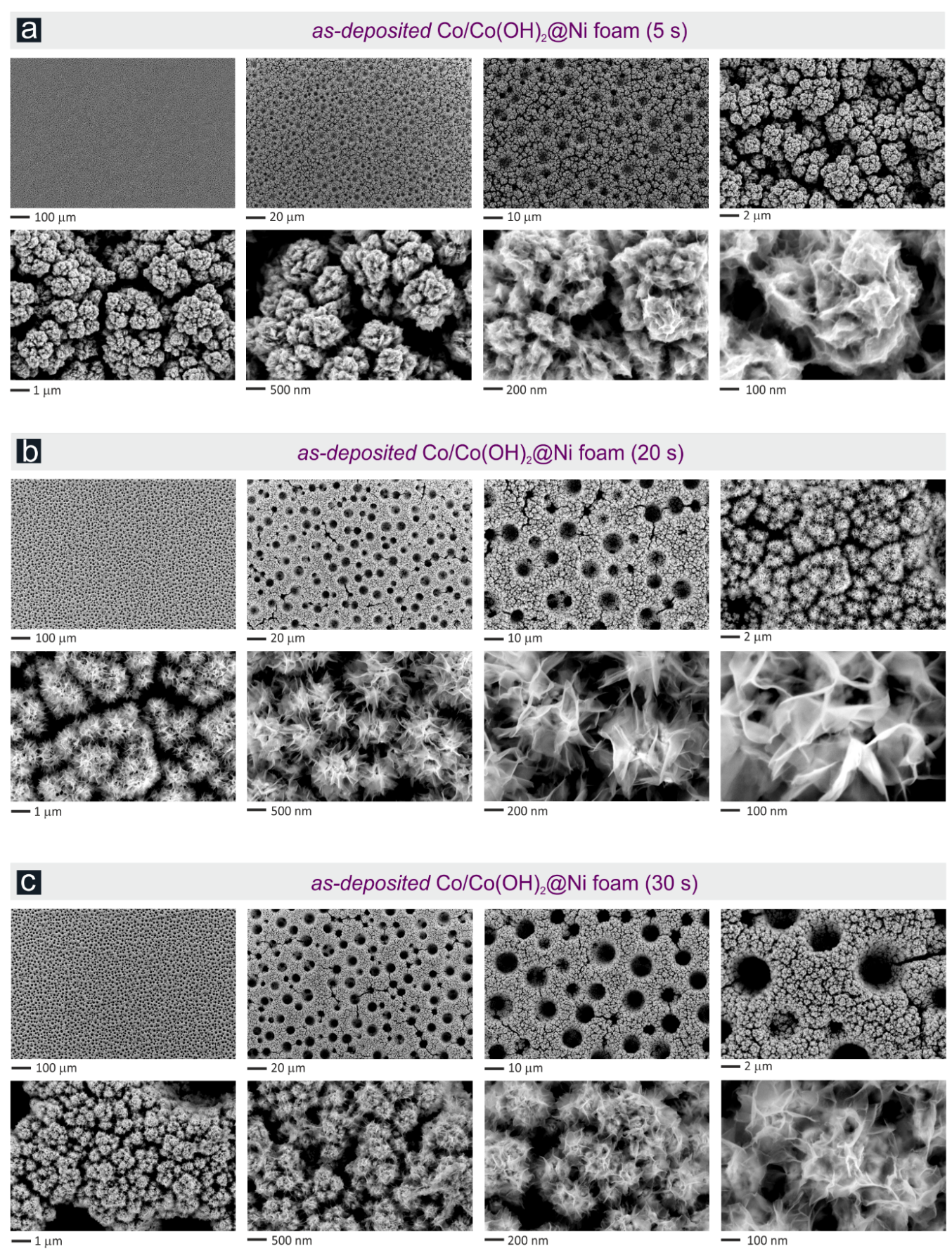


**Figure S6.** Top-down SEM inspection of the as-deposited Co/Co(OH)_2_@Ni foam samples. a) 5 s deposition time. b) 20 s deposition time. c) 30 s deposition time.


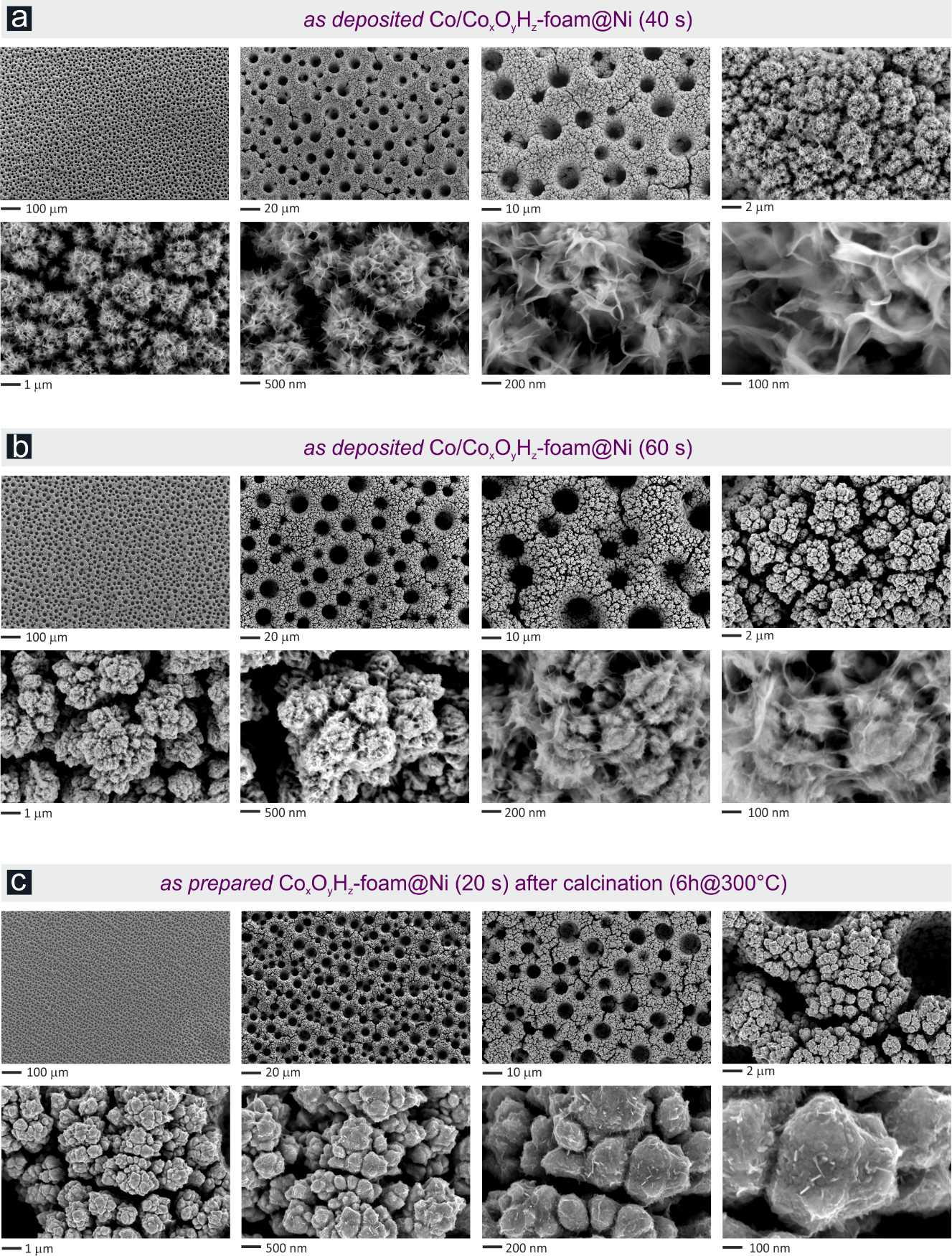


**Figure S7.** Top-down SEM inspection of the as-deposited Co/Co(OH)_2_@Ni foam samples. a) 40 s deposition time. b) 60 s deposition time. c) Co/Co*_x_*O*_y_*H*_z_*@Ni foam following a 20 s Co deposition and subsequent 6 h of thermal treatment (calcination) at 300 °C in air. This foam was used as a catalyst precursor for the routine electrolysis experiments discussed in this work.


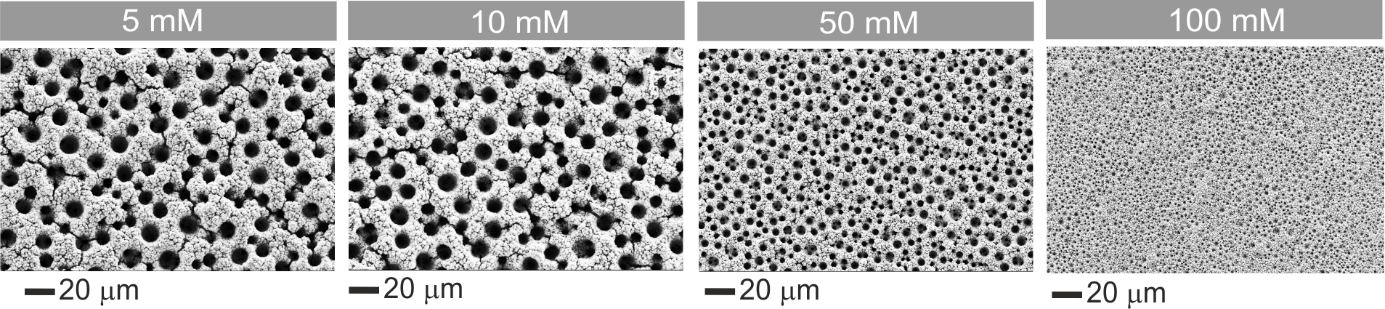


**Figure S8.** Representative top-down SEM inspection of the Co/Co(OH)_2_@Ni foam samples following 20 s of Co deposition performed in the presence of varying concentrations of the sodium citrate monohydrate additive ranging from 5 mmol L^–1^ to 100 mmol L^–1^. The critical diameter of the bubble break-off significantly decreases for citrate concentrations above 10 mmol L^–1^, thus leading to Co foams with reduced surface pore diameters. Citrate was added to the plating bath at a concentration of 10 mM L^–1^ to enhance the adhesion of the formed Co foam to the Ni support, thereby mitigating catalyst delamination during electrolysis under HER conditions.


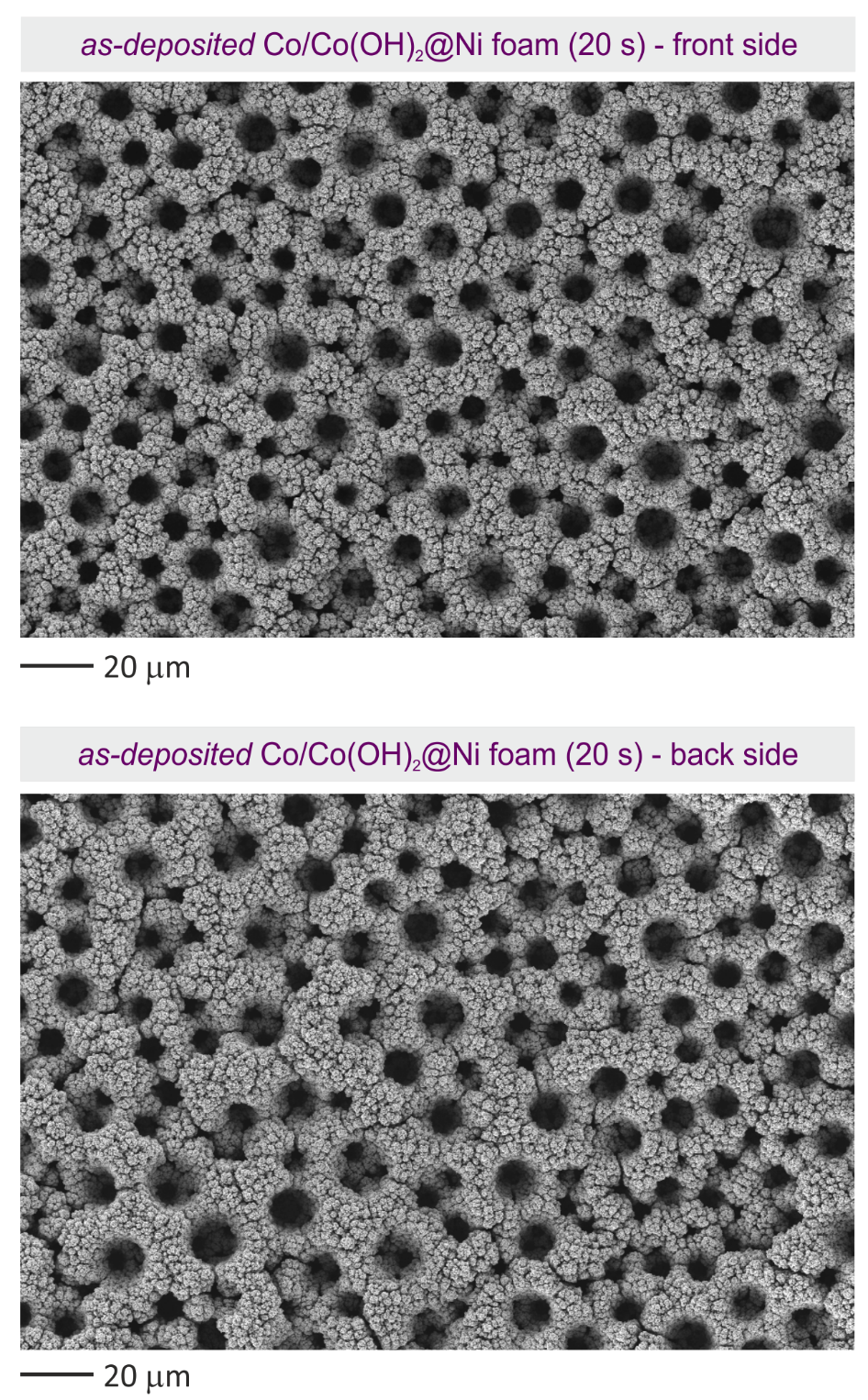


**Figure S9.** Representative top-down SEM inspection of the Co/Co(OH)_2_@Ni foam sample comparing the front and back side of the Ni support electrode following 20 s of Co deposition.


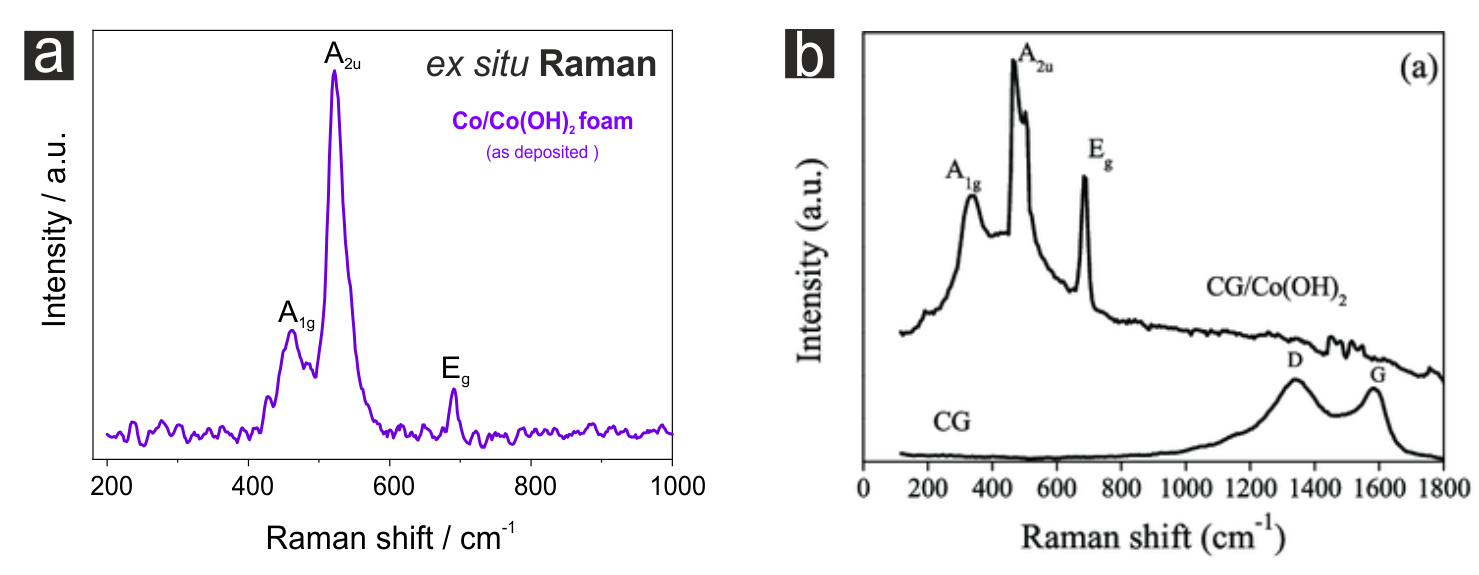


**Figure S10.** a) Representative *ex situ* Raman spectrum of the as-deposited Co/Co(OH)_2_@C foam (following 20 s of deposition) demonstrating the presence of Co(OH)_2_ on the outermost foam surface. Note that neither CoO nor Co_3_O_4_ could be detected by Raman spectroscopy on the as-deposited Co/Co(OH)_2_ foam immediately after drying and prior to thermal annealing. b) Corresponding reference spectrum of Co(OH)_2_ on glassy carbon, reproduced from *Arab. J. Chem.* **2020**, *13*, 3448–3459 under terms of a CC-BY license (© Elsevier, 2020).


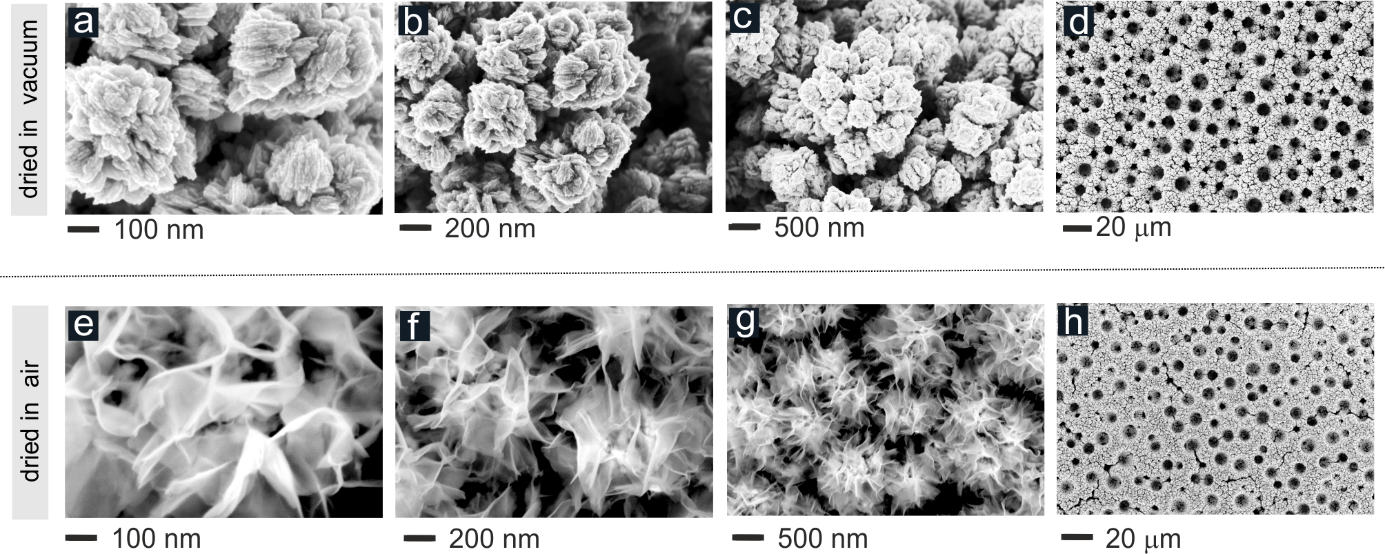


**Figure S11**. a) – d) Top-down SEM inspection of the as-deposited Co/Co(OH)_2_@Ni foam samples following electrodeposition for 20 s, soaking the foam in Milli-Q water for 60 minutes and subsequent drying under vacuum conditions (10^–5^ Pa). There is no indication for surface Co(OH)_2_ formation. e) – h) Respective top-down SEM inspection of the as-deposited Co/Co(OH)_2_@Ni foam samples after drying in an Ar gas stream (Carbagas, Switzerland, 99.999 %) and stored in air. These results confirm that the appearance of Co(OH)_2_ strongly depends on the drying conditions as discussed in the Experimental section of the manuscript.


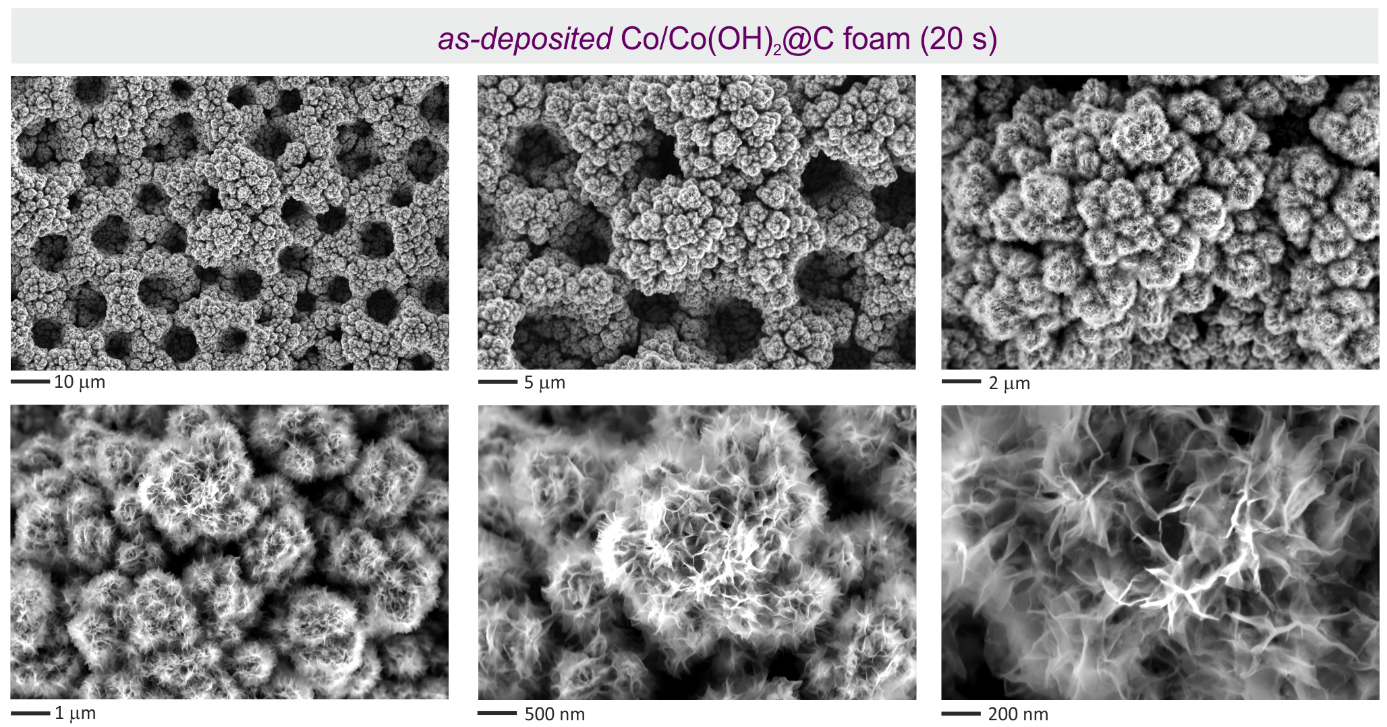


**Figure S12.** Representative top-down SEM analysis of the as-deposited Co/Co(OH)_2_@C foam following a 20 s Co deposition demonstrating similar morphological characteristics as observed for the as-deposited Co/Co(OH)_2_@Ni foam (Figure S6b). Note that the pore size distribution and density at the reported deposition time of 20 s may differ slightly from the corresponding Co/Co(OH)_2_@Ni sample (Figure S6b) due to differences in the average (hydrogen) bubble release diameter when using different substrates for the electro-foaming process.


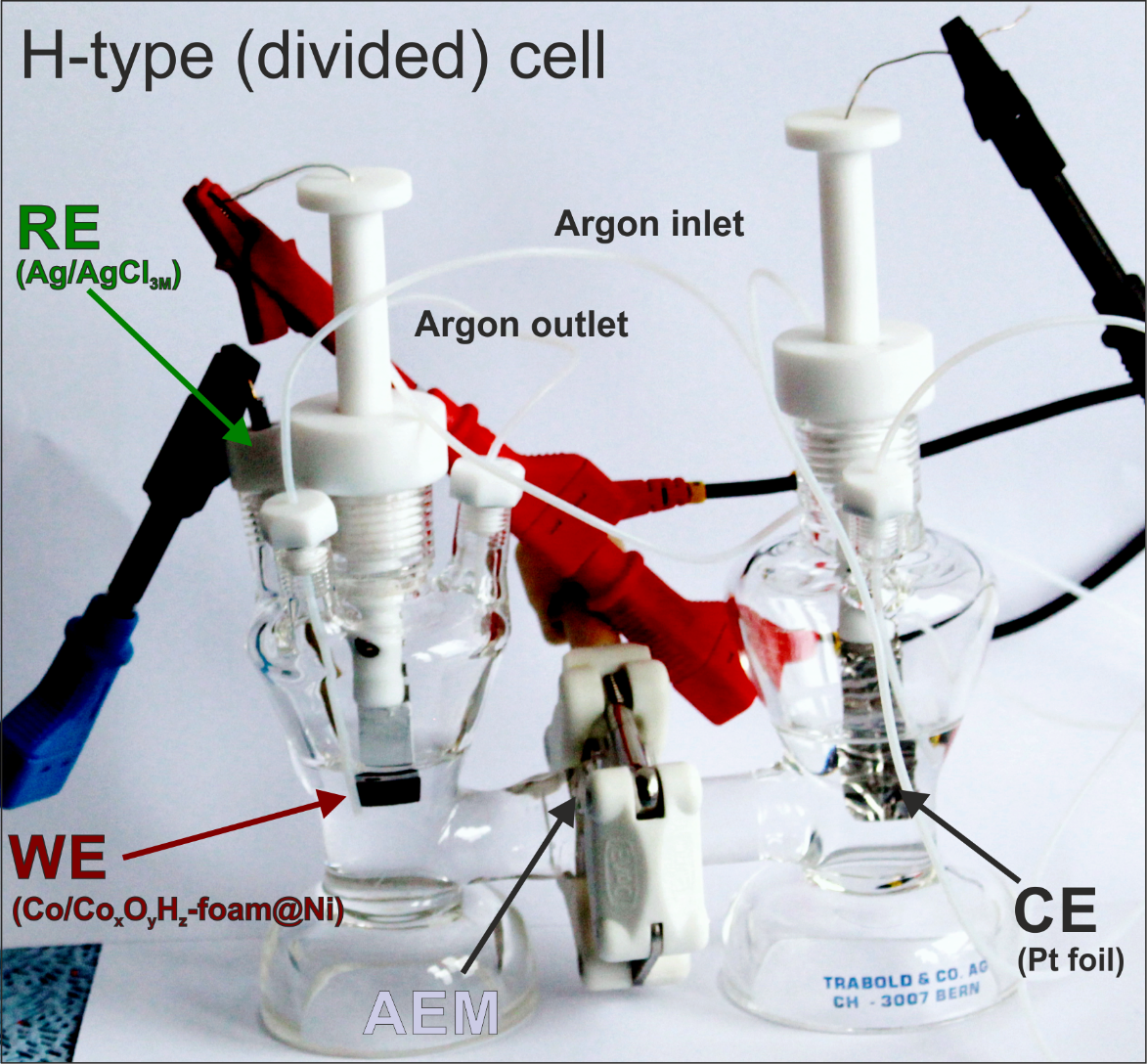


**Figure S13.** Photograph of the customized (divided) H-type electrolysis cell used in this study for voltammetric and potentiostatic electrolysis experiments. The anode and cathode compartments were filled with 15 mL of electrolyte solution. The catholyte was deoxygenated with Ar gas for 30 minutes before the electrochemical experiments were started. Both compartments were separated by an anion exchange membrane (Sustainion, X37-50 RT). A Pt foil served as the counter electrode (CE), an Ag/AgCl/3MKCl electrode was used as the reference electrode (RE), and the Co/Co*_x_*O*_y_*H*_z_* foams served as the working electrode (WE).


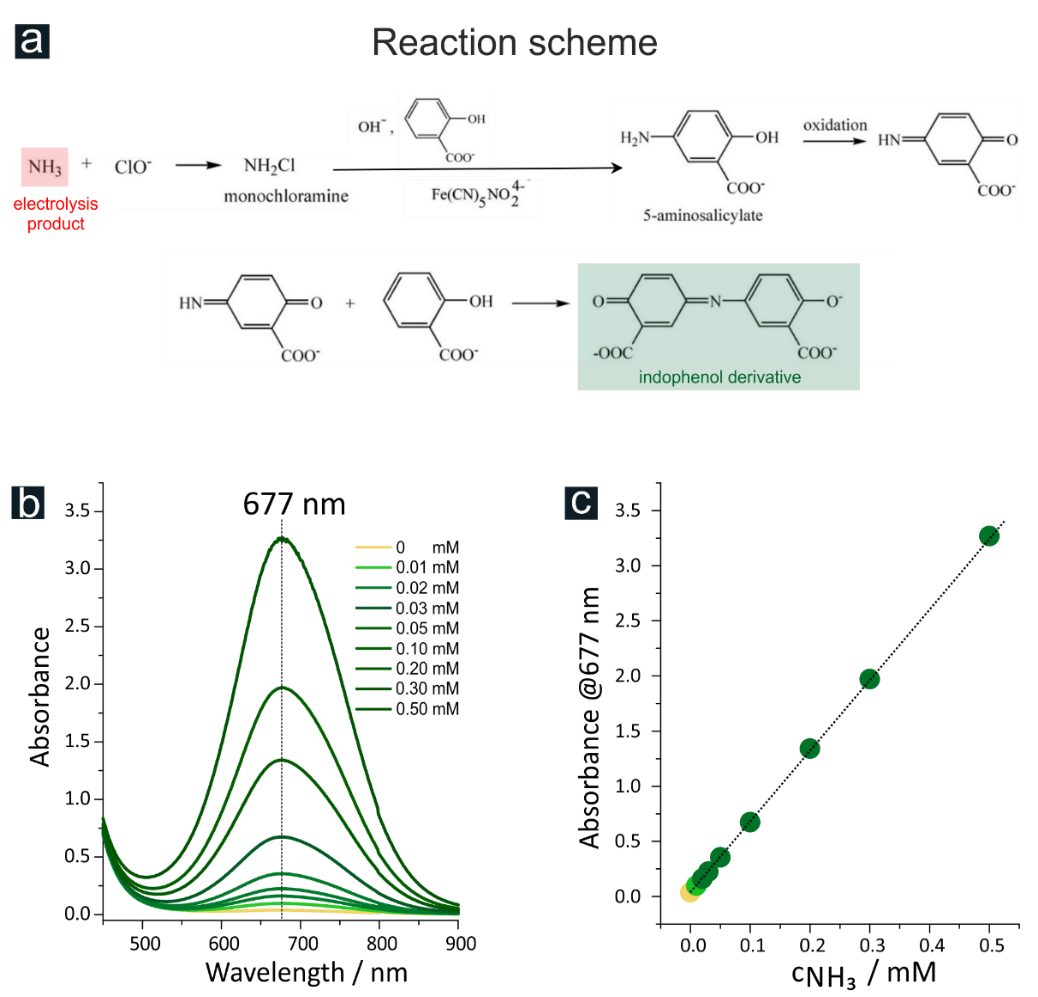


**Figure S14.** a) Reaction scheme showing the transformation of ammonia into an indophenol derivative as the basis for its quantification. b) Set of representative UV-Vis spectra of standard solutions used for calibration purposes. c) Calibration curve showing a linear scaling of the absorbance at 677nm with the ammonia concentration. Note that for ammonia quantification, diluted electrolyte solutions were used resulting in absorbances in the range between 0 and 2.5 (detection limit: 10 µM ammonia concentration).


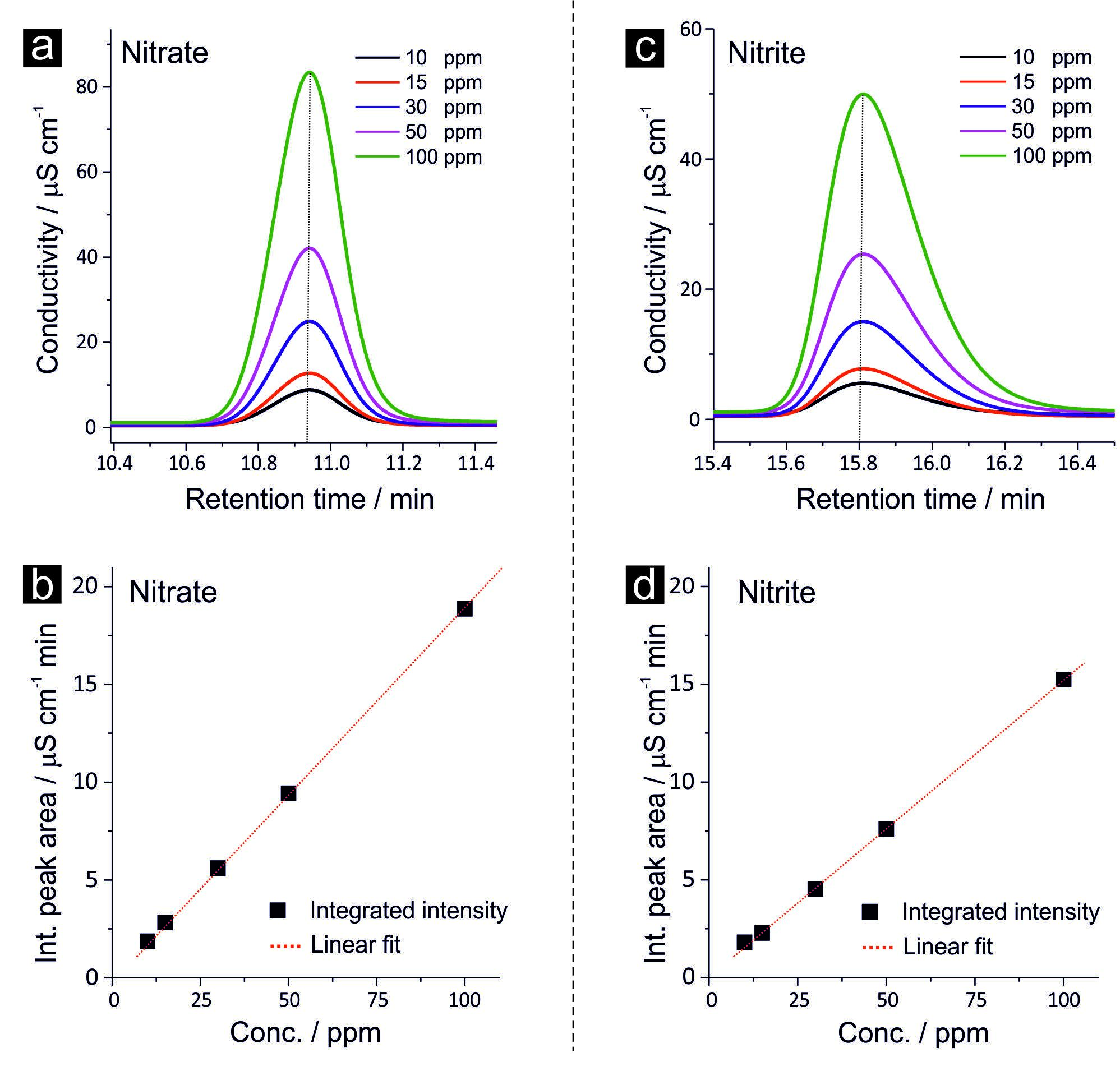


**Figure S15.** a) Representative set of ion exchange chromatograms of nitrate standard solutions. b) Corresponding calibration curve demonstrating a linear relationship between the integrated peak areas of the chromatograms and the nitrate ($NO_{3}^{-}$) concentration. c) Representative set of ion exchange chromatograms of nitrite standard solutions. d) Corresponding calibration curve demonstrating a linear relationship between the integrated peak areas of the chromatograms and the nitrite ($NO_{2}^{-}$) concentration. (Detection limit: 100 ppb).


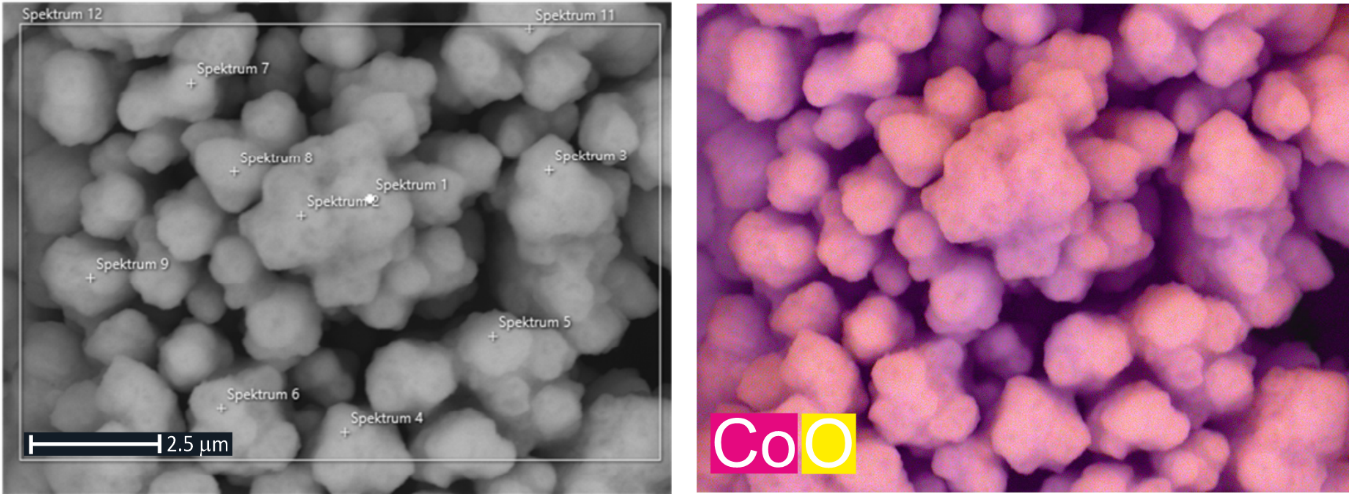


| **Location** | **Atomic % / O** | **Atomic % / Co** |
| --- | --- | --- |
| Spectrum 1 | 54.33 | 45.67 |
| Spectrum 2 | 52.98 | 47.02 |
| Spectrum 3 | 59.14 | 40.86 |
| Spectrum 4 | 54.52 | 45.48 |
| Spectrum 5 | 57.70 | 42.30 |
| Spectrum 6 | 57.72 | 42.28 |
| Spectrum 7 | 56.17 | 43.83 |
| Spectrum 8 | 59.69 | 40.31 |
| Spectrum 9 | 49.68 | 50.32 |
| Spectrum 10 | 57.11 | 42.89 |
| Spectrum 11 | 41.05 | 58.95 |

**Figure S16.** Energy-dispersive X-ray spectroscopy (EDX, K^α^ signals) mapping and point analysis were performed on cobalt foam samples subjected to calcination at 300 °C for 6 hours.


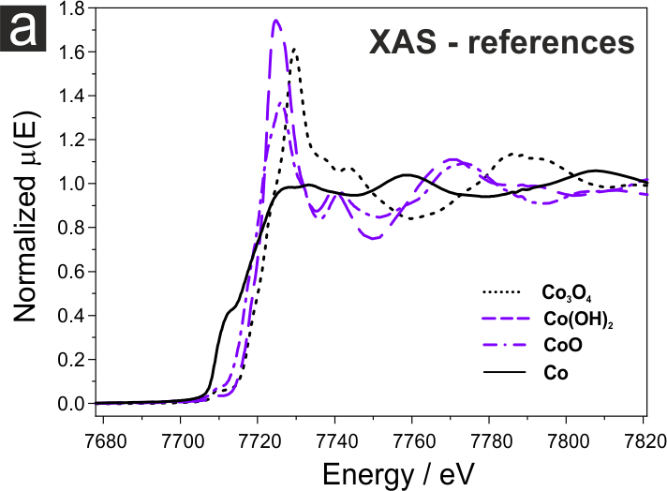


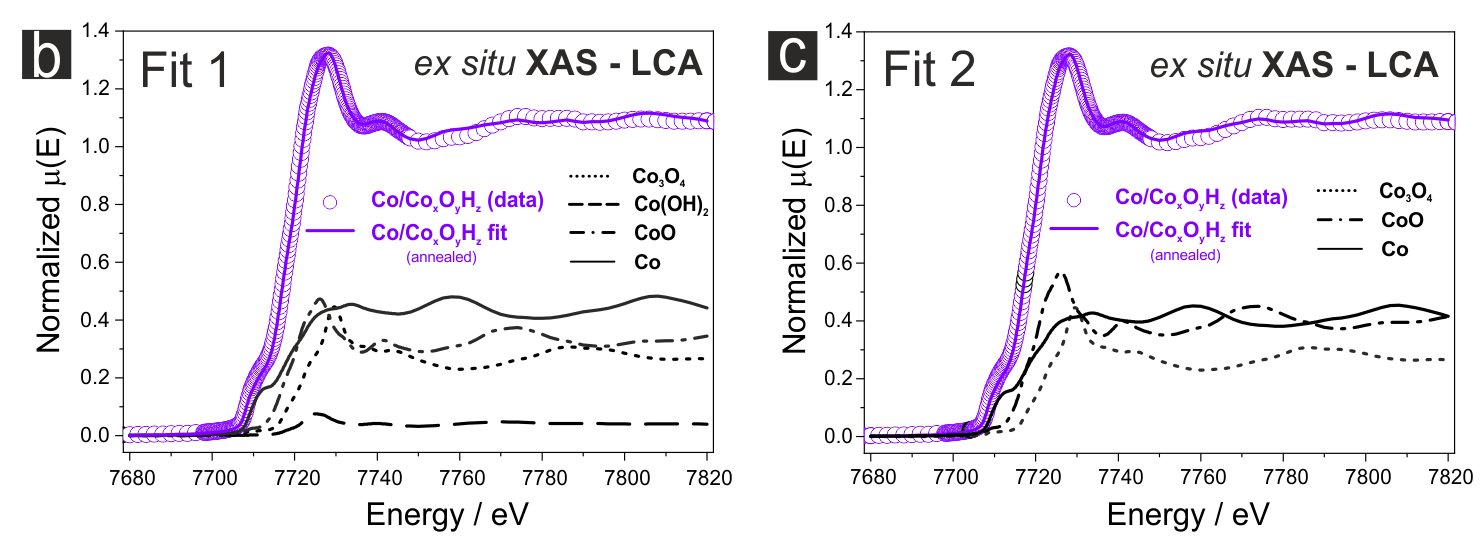


|  | **LCA weights** | | | | ***χ*^2^** |
| --- | --- | --- | --- | --- | --- |
|  | **Co** | **Co(OH)_2_** | **CoO** | **Co_3_O_4_** |  |
| Fit1 | 0.431441 | 0.035763 | 0.297034 | 0.240339 | 0.019 |
| Fit2 | 0.405678 | 0 | 0.358305 | 0.240593 | 0.023 |


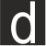


**Figure S17.** a) XAS spectra of reference materials serving as the basis for the linear combination analysis (LCA) of the Co/Co*_x_*O*_y_*H*_z_*@C foam samples. b) LCA (*ex situ* XAS data) of a Co/Co*_x_*O*_y_*H*_z_*@C foam (following thermal annealing for 6h@300 °C) considering Co, Co(OH)_2_, CoO, and Co_3_O_4_ references (denoted Fit 1). c) LCA (*ex situ* XAS data) of the same Co/Co*_x_*O*_y_*H*_z_*@C foam, omitting the Co(OH)_2_ component (denoted Fit 2). d) Table detailing the chemical composition of the Co/Co*_x_*O*_y_*H*_z_* foam according to Fit 1 and Fit 2.


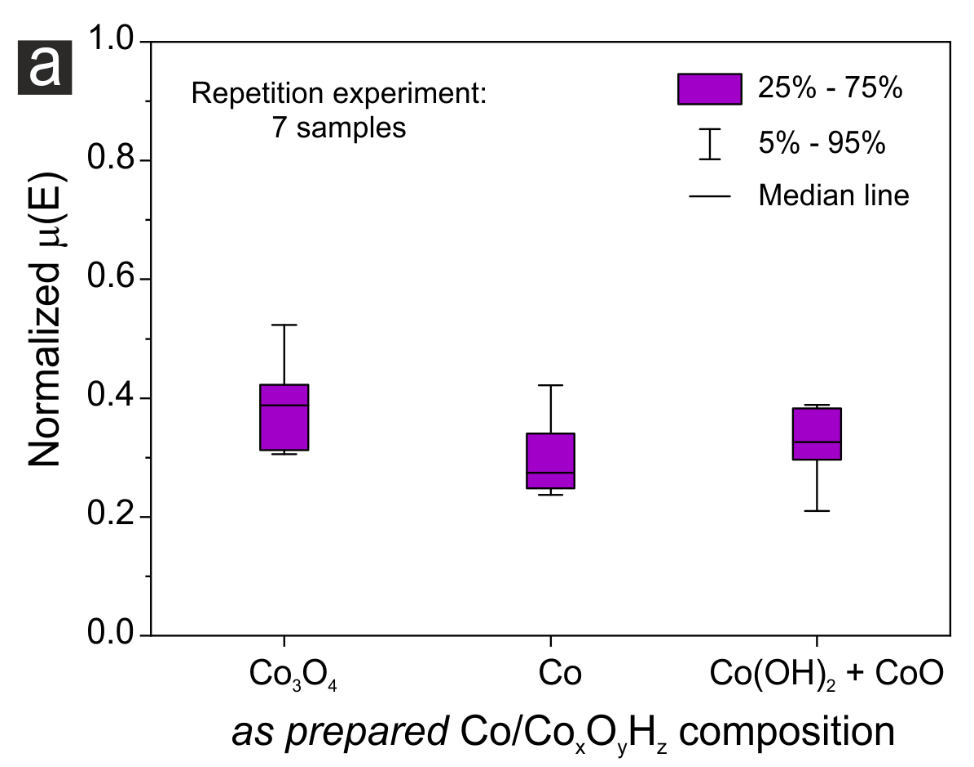


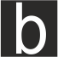


|  | **LCA weights** | | |
| --- | --- | --- | --- |
|  | **Co** | **CoO + Co(OH)_2_** | **Co_3_O_4_** |
| Sample 1 | 0.23690 | 0.38875 | 0.38772 |
| Sample 2 | 0.42175 | 0.29673 | 0.31248 |
| Sampel 3 | 0.24773 | 0.34615 | 0.42069 |
| Sample 4 | 0.27396 | 0.32621 | 0.42275 |
| Sample 5 | 0.26719 | 0.20971 | 0.52328 |
| Sample 6 | 0.34050 | 0.38307 | 0.30539 |
| Sample 7 | 0.30834 | 0.31467 | 0.37850 |

**Figure S18.** a) Plot showing the average chemical composition of as-prepared Co/Co*_x_*O*_y_*H*_z_* foam (following thermal annealing for 6h@300°C) considering the LCA analysis of seven individual samples. b) Corresponding numerical data of the Co/Co*_x_*O*_y_*H*_z_* foams analyzed.


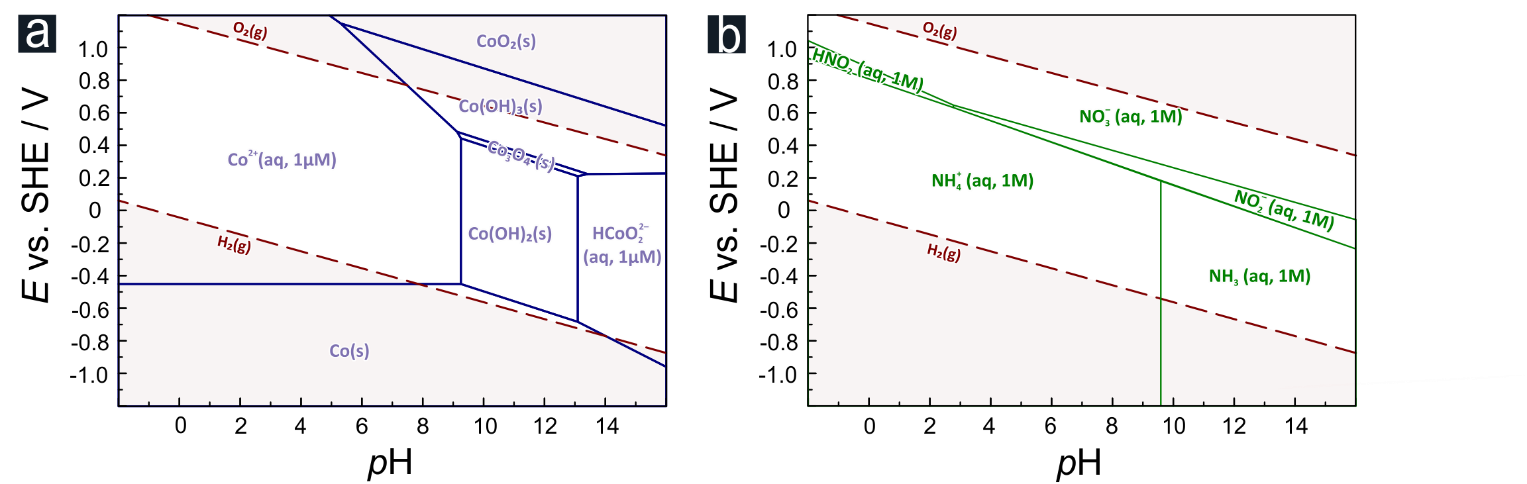


**Figure S19.** a) Pourbaix diagram of the aqueous Co system assuming a Co^2+^ ion concentration of 10^–6^ mol L^–1^. The HER and the OER are indicated by the dashed red lines. b) Pourbaix diagram of the aqueous system relevant to the NO_3_RR.

For constructing the diagrams, unity activity of components present in the gas or solid phase was assumed, while for dissolved species the activities shown by the labels of Figure S19 were used. The following equilibria and corresponding equations were applied in all calculations, where all standard potentials all meant vs. SHE, and [S] denotes the absolute activity of species S, referenced to the standard 1 mol L^–1^ molar concentration:

**Hydrogen and oxygen evolution equilibria:**

$2H^{+}+2e^{-}\rightleftharpoons H_{2}$ $E= -0.0591 V\cdot pH$

$O_{2}+4e^{-}+4H^{+}\rightleftharpoons2H_{2}O$ $E=1.230 V -0.0591 V\cdot pH$

**Equilibria involving Co containing species:**

$\mathrm{Co}^{2+}+2e^{-}\rightleftharpoons Co$ $E= -0.277 V+0.0295 V\cdot\log\left[ \mathrm{Co}^{2+} \right]$

$\mathrm{Co}\left( \mathrm{OH} \right)_{2}+2e^{-}+2H^{+}\rightleftharpoons Co+2H_{2}O$ $E=0.095 V -0.0591 V\cdot pH$

$\mathrm{Co}{(OH)}_{2}+{2H}^{+}\rightleftharpoons\mathrm{Co}^{2+}+2H_{2}O$ $pH=6.30 -\frac{1}{2}\log\left[ \mathrm{Co}^{2+} \right]$

$\mathrm{Co}{(OH)}_{2}\rightleftharpoons\mathrm{HCoO}_{2}^{-}+H^{+}$ $pH=19.10+\log\left[ \mathrm{HCoO}_{2}^{-} \right]$

$\mathrm{Co}_{3}O_{4}+8H^{+}+2e^{-}\rightleftharpoons{3Co}^{2+}+4H_{2}O$ $E=2.112 V -0.2364 V\cdot pH -0.0886 V\cdot\log\left[ \mathrm{Co}^{2+} \right]$

$\mathrm{Co}_{3}O_{4}+2H_{2}O +2e^{-}\rightleftharpoons3\mathrm{HCoO}_{2}^{-}+H^{+}$ $E= -0.700 V +0.0295 V\cdot pH -0.0886 V\cdot\log\left[ {HCoO}_{2}^{-} \right]$

$\mathrm{Co}_{3}O_{4}+2H^{+} +2H_{2}O+2e^{-}\rightleftharpoons3 Co{(OH)}_{2}$ $E= 0.993 V -0.0591 V\cdot pH$

$3Co{(OH)}_{3}+H^{+} +e^{-}\rightleftharpoons\mathrm{Co}_{3}O_{4}+5H_{2}O$ $E= 1.018 V -0.0591 V\cdot pH$

$\mathrm{Co}{(OH)}_{3}+3H^{+} +e^{-}\rightleftharpoons\mathrm{Co}^{2+}+3H_{2}O$ $E= 1.746 V -0.1773 V\cdot pH-0.0591 V\cdot\log\left[ \mathrm{Co}^{2+} \right]$

$\mathrm{Co}{(OH)}_{3}+e^{-}\rightleftharpoons\mathrm{HCoO}_{2}^{-}+H_{2}O$ $E= -0.128 V -0.0591 V\cdot\log\left[ \mathrm{HCoO}_{2}^{-} \right]$

$\mathrm{CoO}_{2}+H^{+} +H_{2}O+e^{-}\rightleftharpoons Co{(OH)}_{3}$ $E= 1.477 V -0.0591 V\cdot pH$

$\mathrm{CoO}_{2}+4H^{+} +2e^{-}\rightleftharpoons\mathrm{Co}^{2+}+2H_{2}O$ $E= 1.612 V -0.1182 V\cdot pH-0.0295 V\cdot\log\left[ \mathrm{Co}^{2+} \right]$

**Equilibria involving N containing species:**

$\mathrm{NH}_{4}^{+}\rightleftharpoons\mathrm{NH}_{3}+H^{+}$ $pH= 9.27+\log\frac{\left[ {NH}_{3} \right]}{\left[ {NH}_{4}^{+} \right]}$

$\mathrm{NO}_{2}^{-}+7H^{+} +6e^{-}\rightleftharpoons\mathrm{NH}_{3}+2H_{2}O$ $E= 0.806 V -0.0689 V\cdot pH+0.0098 V\cdot\log\frac{\left[ \mathrm{NO}_{2}^{-} \right]}{\left[ \mathrm{NH}_{3} \right]}$

$\mathrm{NO}_{2}^{-}+8H^{+} +6e^{-}\rightleftharpoons\mathrm{NH}_{4}^{+}+2H_{2}O$ $E= 0.897 V -0.0788 V\cdot pH+0.0098 V\cdot\log\frac{\left[ \mathrm{NO}_{2}^{-} \right]}{\left[ \mathrm{NH}_{4}^{+} \right]}$

$\mathrm{HNO}_{2}+7H^{+} +6e^{-}\rightleftharpoons\mathrm{NH}_{4}^{+}+2H_{2}O$ $E= 0.864 V -0.0689 V\cdot pH+0.0098 V\cdot\log\frac{\left[ \mathrm{HNO}_{2} \right]}{\left[ \mathrm{NH}_{4}^{+} \right]}$

$\mathrm{HNO}_{2}\rightleftharpoons\mathrm{NO}_{2}^{-}+H^{+}$ $pH= 3.35+\log\frac{\left[ {NO}_{2}^{-} \right]}{\left[ {HNO}_{2} \right]}$

$\mathrm{NO}_{3}^{-}+3H^{+}+2e^{-}\rightleftharpoons\mathrm{HNO}_{2}+H_{2}O$ $E= 0.934 V -0.0886 V\cdot pH+0.0295 V\cdot\log\frac{\left[ \mathrm{NO}_{3}^{-} \right]}{\left[ \mathrm{HNO}_{2} \right]}$

$\mathrm{NO}_{3}^{-}+2H^{+}+2e^{-}\rightleftharpoons\mathrm{NO}_{2}^{-}+H_{2}O$ $E= 0.835 V -0.0591 V\cdot pH+0.0295 V\cdot\log\frac{\left[ \mathrm{NO}_{3}^{-} \right]}{\left[ \mathrm{NO}_{2}^{-} \right]}$

Source of data: M. Pourbaix, Atlas of Electrochemical Equilibria in Aqueous Solutions, National Association of Corrosion Engineers, Houston TX, 1974, 2nd edition.


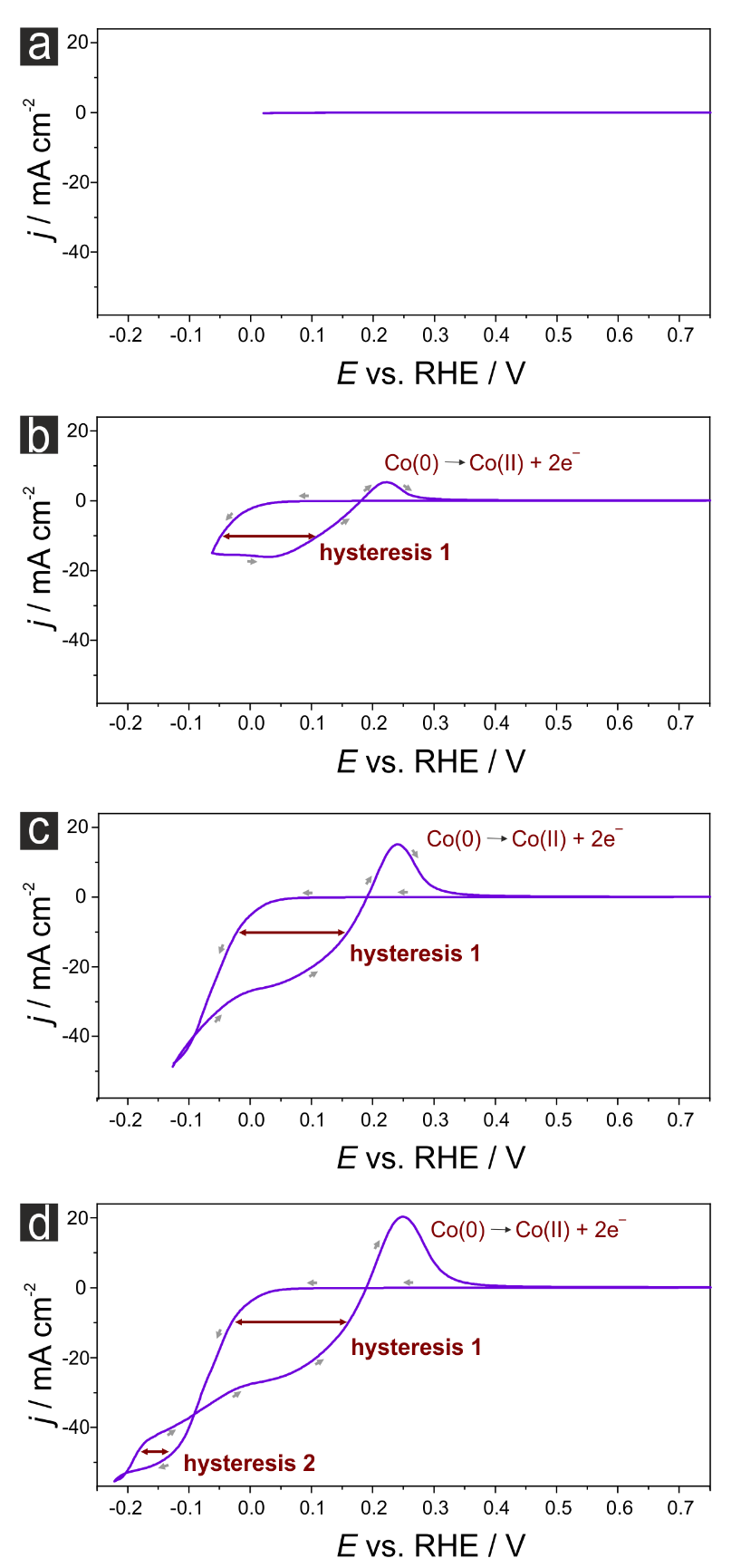


**Figure S20.** Variation of the cathodic vertex potential: Cyclic voltammograms (CVs) recorded in 1 mol L^–1^ KOH + 0.1 mol L^–1^ KNO_3_ solution (pH 13.7) using the Co/Co*_x_*O*_y_*H*_z_*@Ni foam as the WE. The potential sweep rate (d*E*/d*t*) was 10 mV s^–1^.


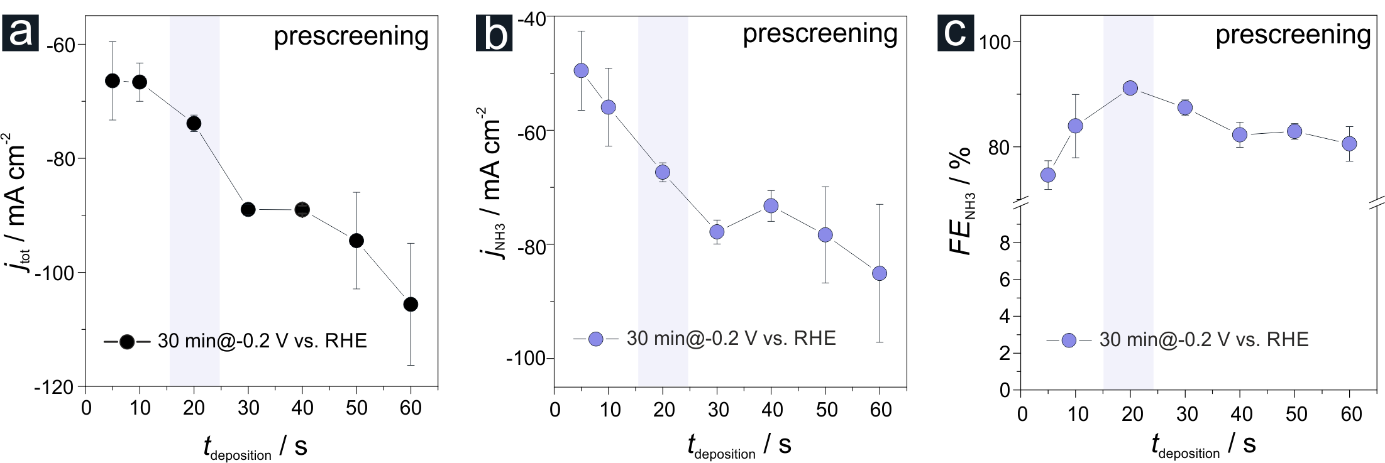


**Figure S21.** Prescreening of catalyst precursors using Co/Co*_x_*O*_y_*H*_z_*@Ni differing in the duration of the deposition time used for the initial metal foaming. The 30 minutes lasting potentiostatic prescreening experiments were performed in 1 mol L^–1^ KOH + 0.1 mol L^–1^ KNO_3_ solution (pH 13.7) at –0.3 V vs. RHE. a) Plot of the total current densities (*j*_tot_) achieved for the different Co/Co*_x_*O*_y_*H*_z_*@Ni catalysts. b) Corresponding plot of the partial current densities for ammonia (*j*_NH₃_). c) Corresponding Faradaic efficiencies for ammonia production. The presented data are derived from two independent electrolyses per experimental setting. Highlighted in grey are the Co/Co*_x_*O*_y_*H*_z_*@Ni samples (20 s deposition time) selected for the in-depth analyses described in the main manuscript. (See Table S3 for the numerical data).


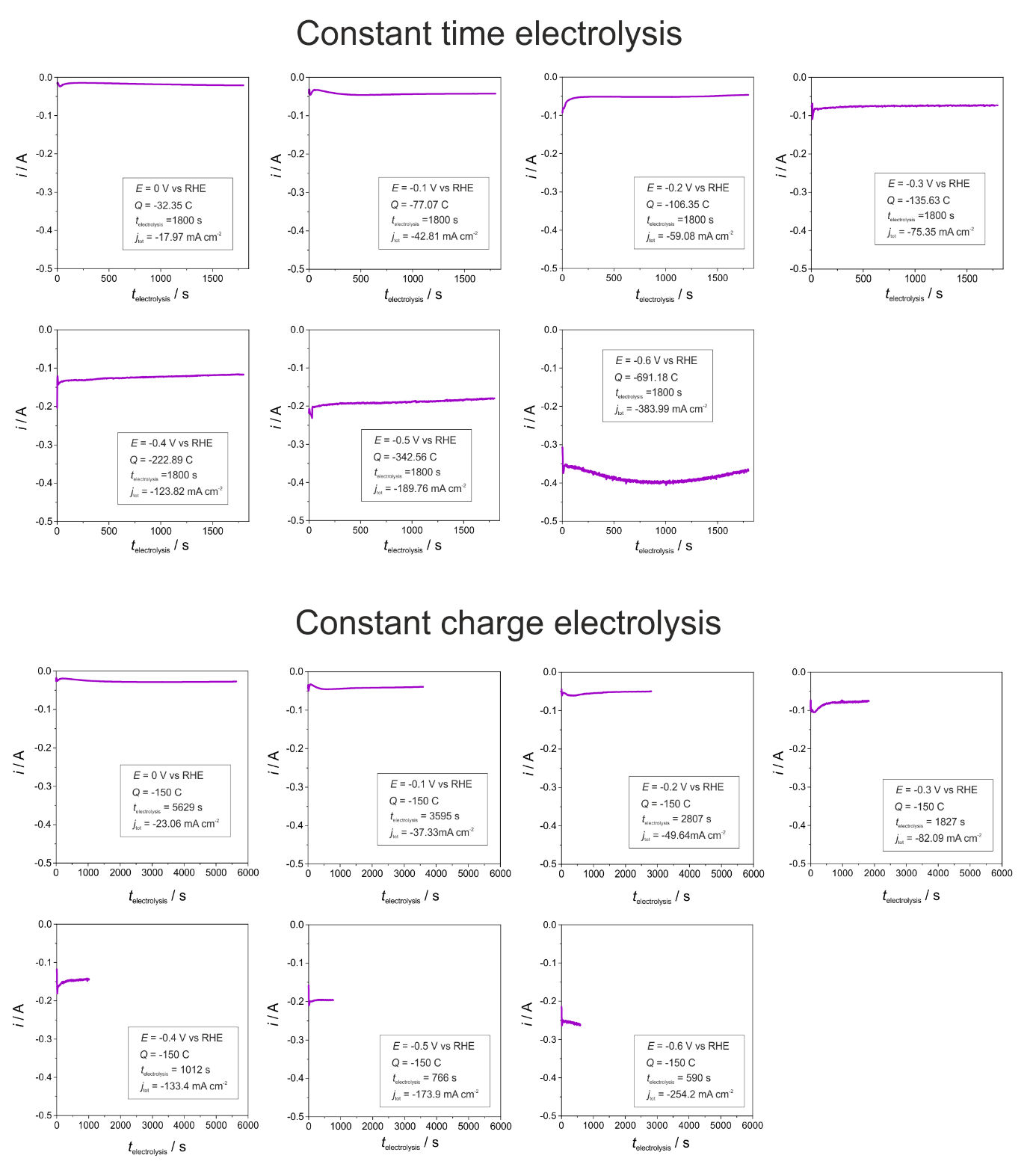


**Figure S22.** Representative data set showing current (*i*) versus electrolysis time (*t*_electrolysis_) transient measurements corresponding to Figure 4 in the main manuscript. Their integration serves as the basis for the calculation of the mean (total and partial) current densities presented in Figure 4. Note that for the actual calculation, triplicate measurements were considered.


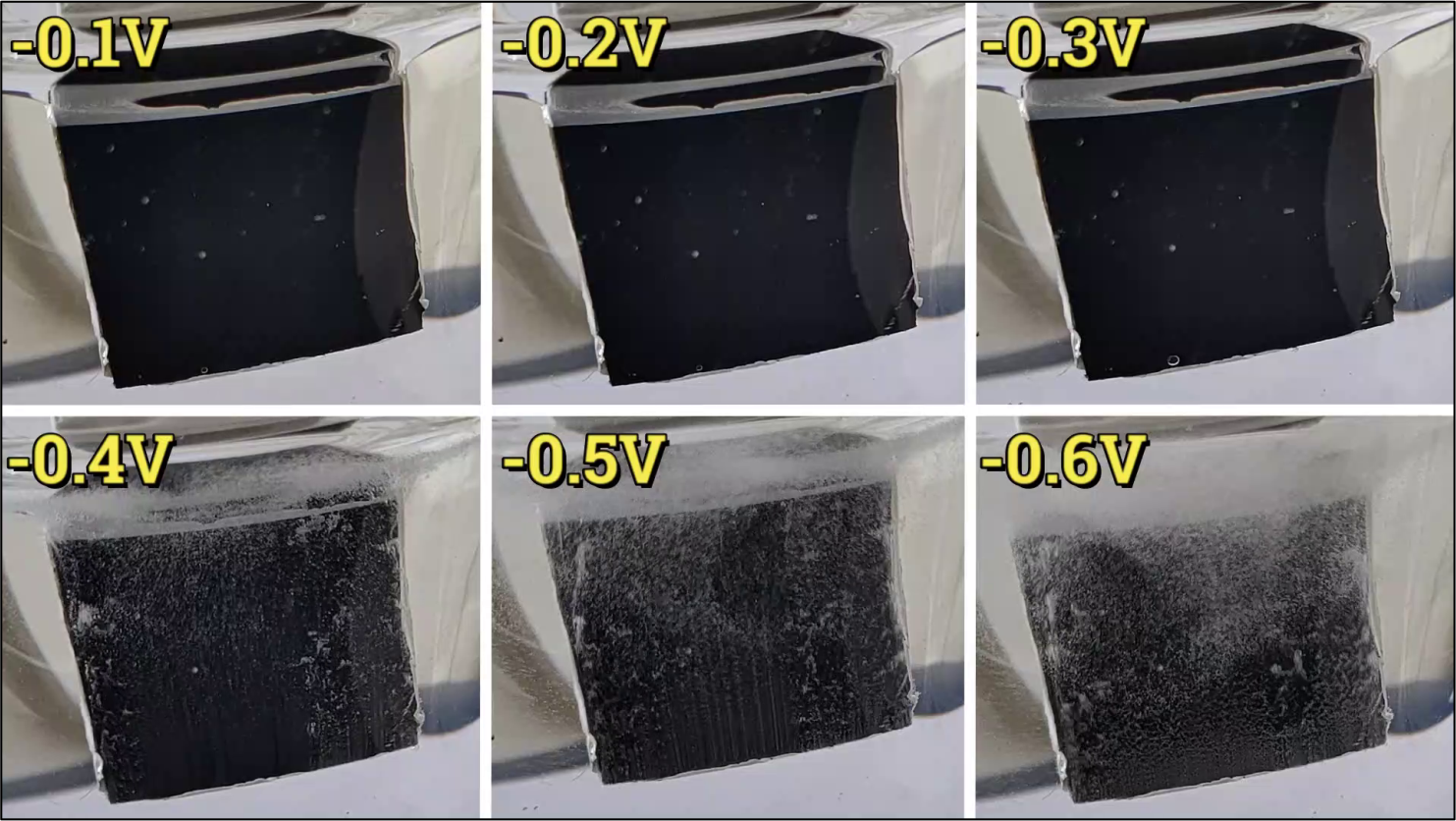


**Figure S23.** Photographs taken during potentiostatic electrolysis demonstrating massive gas evolution at the immersed Co/Co*_x_*O*_y_*H*_z_*@Ni catalyst which can be attributed to the HER at potentials ≤ –0.4 V vs. RHE. The constant electrolysis potentials are indicated in the respective image.


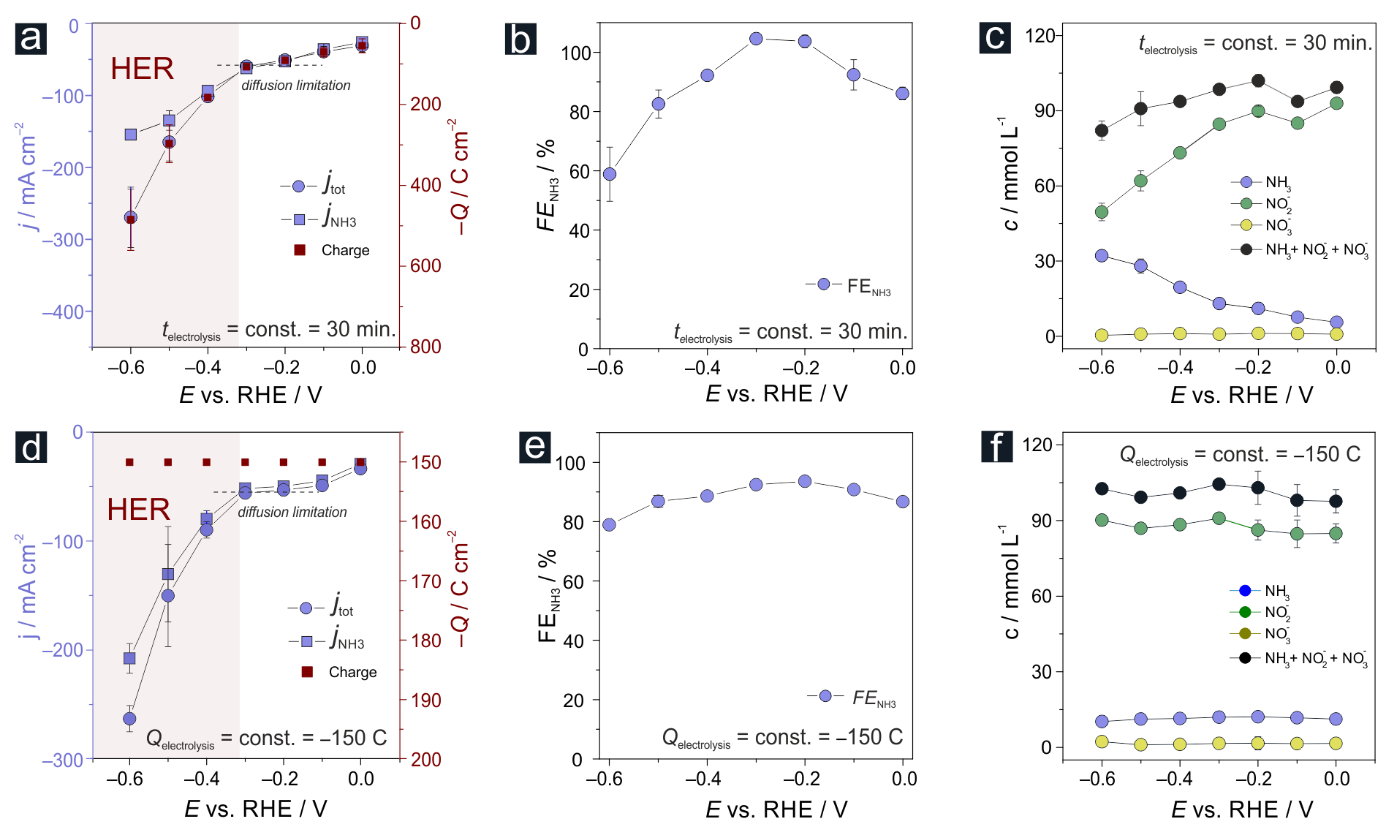


**Figure S24.** Electrocatalytic performance of the Co/Co*_x_*O*_y_*H*_z_*@Ni foam towards $NO_{2}^{-}\mathrm{RR}$. a) *(j* vs. *E*) plot of potentiostatic electrolyses performed in 1 mol L^–1^ KOH + 0.1 mol L^–1^ KNO_2_ solution (pH 13.7); the electrolyses were normalized to a constant electrolysis time (telectrolysis) of 30 minutes. b) Corresponding (*FE*_NH₃_ *vs.* *E*) plot. c) Corresponding (*c* vs. *E*) plot representing the electrolyte composition after the respective time-normalized electrolysis. d) (*j* *vs. E*) plot of potentiostatic electrolyses performed in 1 mol L^–1^ KOH + 0.1 mol L^–1^ KNO_2_ solution (pH 13.7); electrolyses were normalized to a constant charge (*Q*_electrolysis_) of –150 C. e) Corresponding (*FE*_NH₃_ vs. *E*) plot. f) Corresponding (*c vs. E*) plot representing the electrolyte composition after the respective charge-normalized electrolysis. Note that in panels a and d, the potential regime corresponding to significant hydrogen gas evolution and associated convective effects is highlighted in red. (See Table S5 for numerical data.)


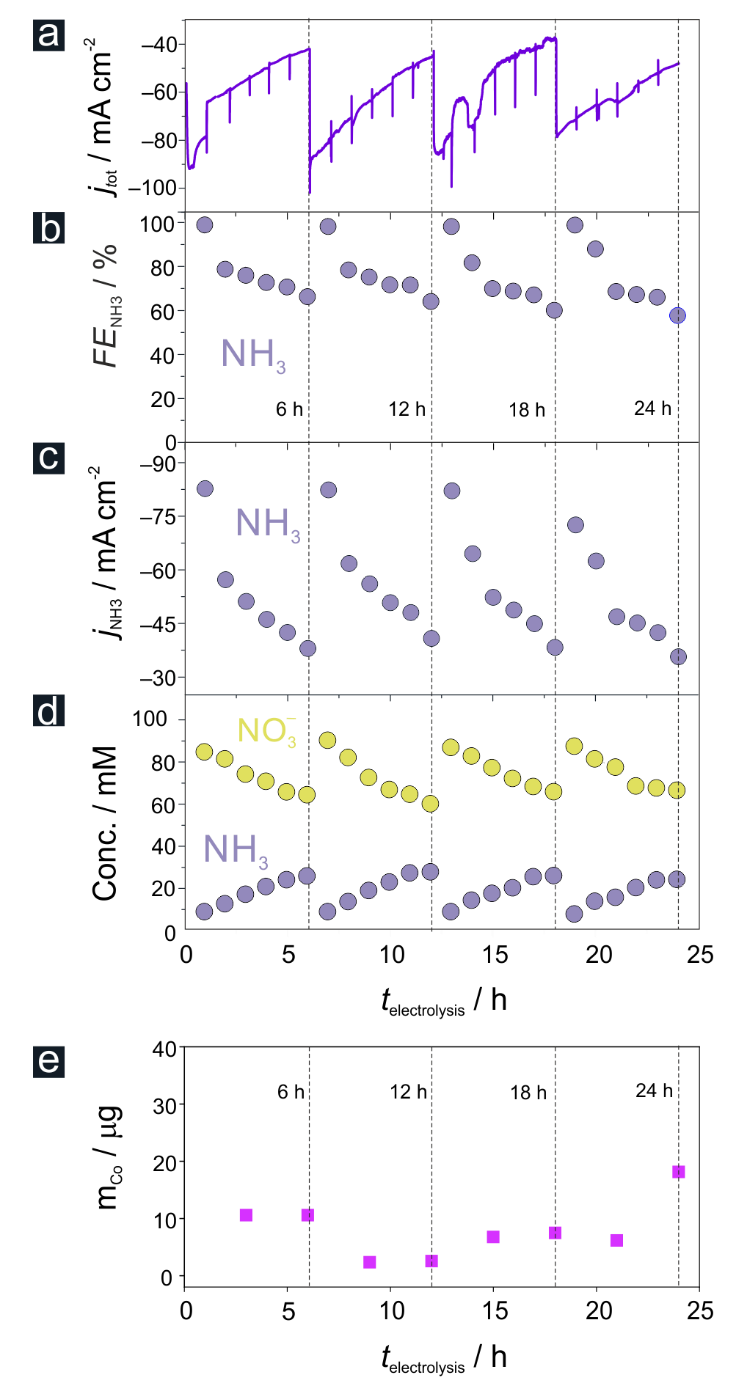


**Figure S25.** Discontinuous 24-hours electrolysis carried out at –0.3 V vs. RHE in 1 mol L^–1^ KOH + 0.1 mol L^–1^ KNO_3_ electrolyte solution (pH 13.7). The overall electrolysis time was subdivided into intervals of six-hours lasting continuous electrolyses. After six-hours the Co/Co*_x_*O*_y_*H*_z_*@Ni foam was removed from the electrolysis cell and the electrolyte was replenished before restarting the electrolysis. a) Time-dependent total current density (*j*_tot_). b) Time-dependent Faradaic efficiency for ammonia (*FE*_NH₃_). c) Time-dependent partial current density for ammonia (*j*_NH₃_). d) Time-dependent concentration changes. e) Time-dependent mass of Co detected in the electrolyte by means of ICP-MS.


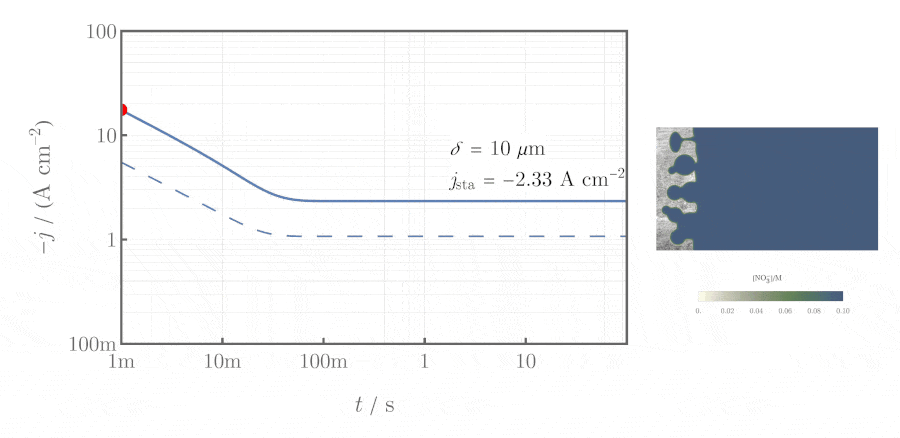

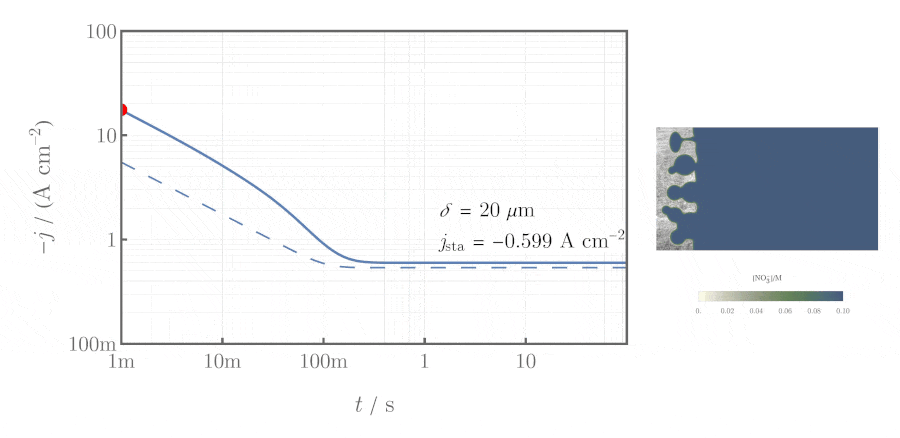

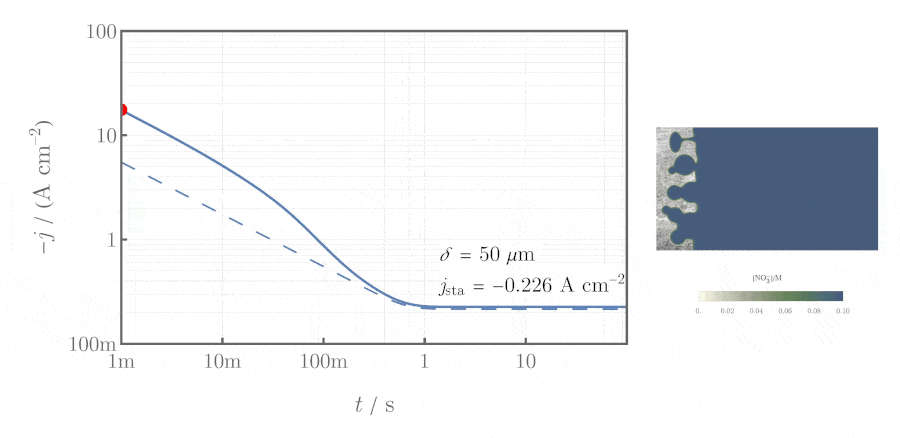

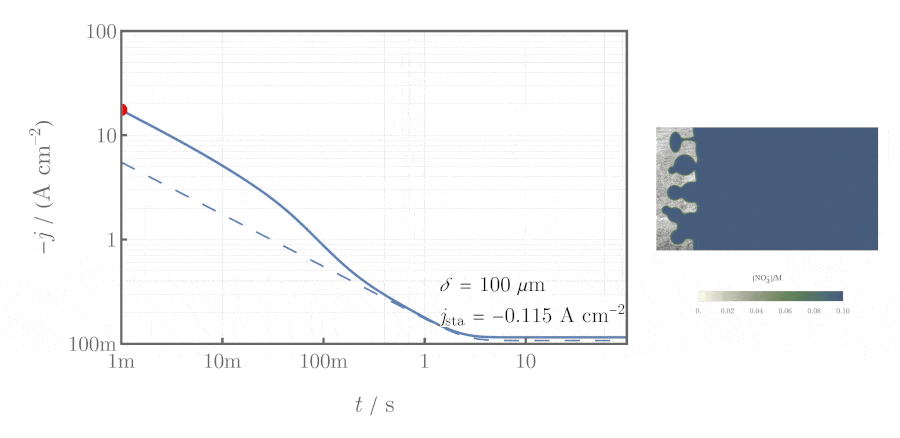


**Figure S26.** Steady-state concentration profiles (Figures 5c–f of the main text) and current densities (Figure 5a of the main text) were calculated using a 2D diffusion simulation, shown here in the form of animation (click an image to start). The simulation is based on an assumed schematic electrode geometry based on a scanning electron microscope (SEM) image of a Co foam (Figure 5b), where pore sizes were estimated based on the assumption of closely spherical pores that result in the given lateral SEM view. The home-written simulation algorithm utilized a Cartesian grid with 400 nm cell size. At the beginning of the simulation, each (solution containing) cell was set to contain nitrate in a 0.1 M (bulk) concentration. In each simulation step, the concentration of the near-surface cells was zeroed (thus contributing to the simulated current), and the discretized version of Fick’s diffusion equation (with a diffusion coefficient of 1.6 ·10^–5^ cm2 s^–1^) was solved over the simulation grid to propagate the effect of near-surface concentration changes. Finally, in order to model the effect of convection, the concentration of cells that lied out of a *δ* Euclidean distance from the surface was set to the bulk concentration. In order to simulate the diffusion process, a fixed time step of 1 ms was used and new concentrations over the grid were calculated from the previous ones by using a truncated Gaussian filter approximation of the exponential of the discrete Laplace operator. See *J. Electrochem. Sci. Eng.* **2018**, *8*, 171 for more detailed background.


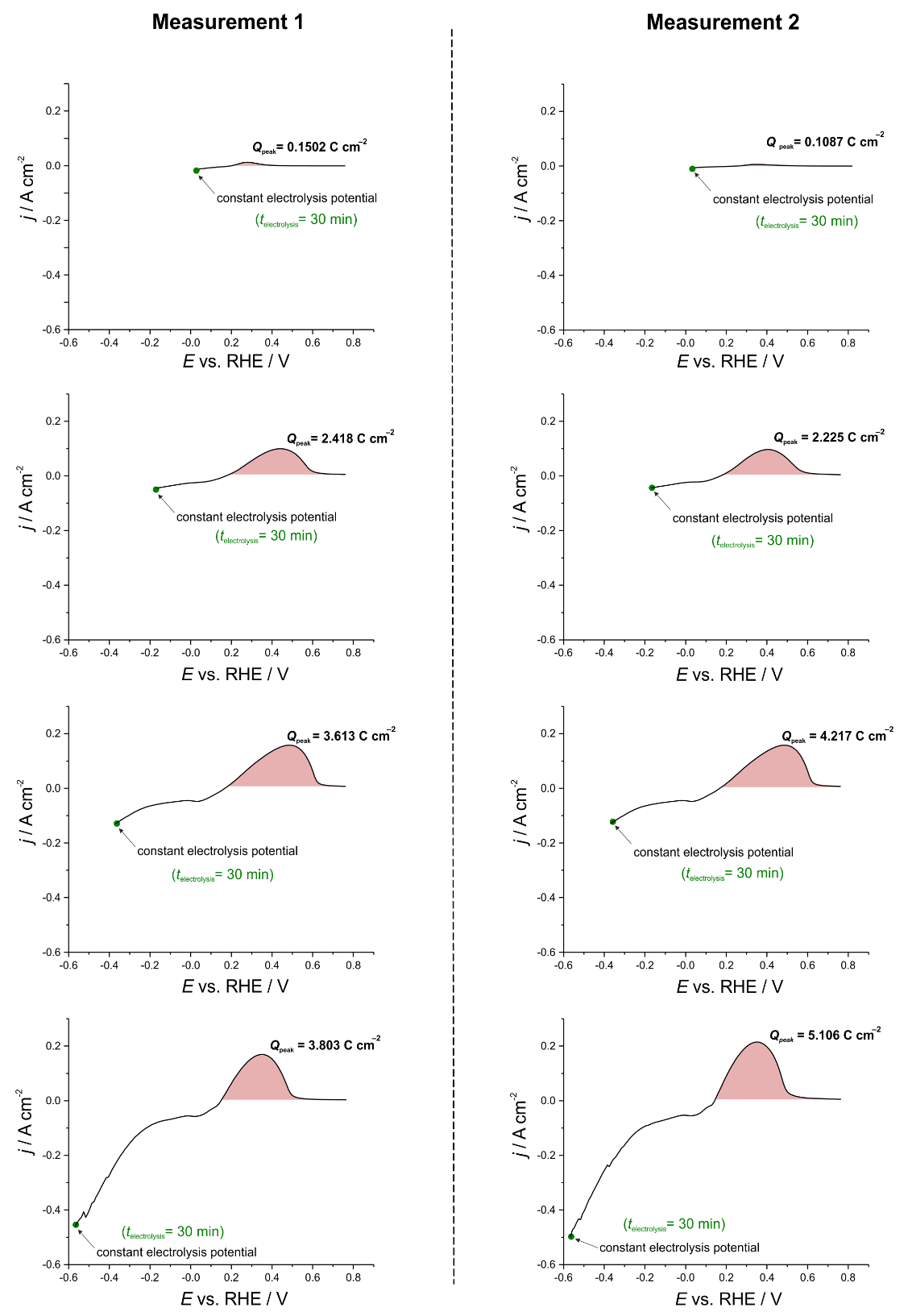


**Figure S27.** Linear sweep voltammograms (d*E*/d*t* = 10 mV s^–1^) following constant time electrolysis (*t*_electrolysis_= 30 minutes) performed at varying potentials. The respective electrolysis potential served as the starting point for the backward LSV passing the oxidation peak assigned to the Co(0)/Co(II) transition. The charge involved in the Co(0)/Co(II) transition was derived through peak integration after correction for capacitive currents. The derived charge is indicated next to the peak. Measurements 1 and 2 refer to two independent measurements campaigns. For each electrolysis and backward LSV, a fresh catalyst was used.


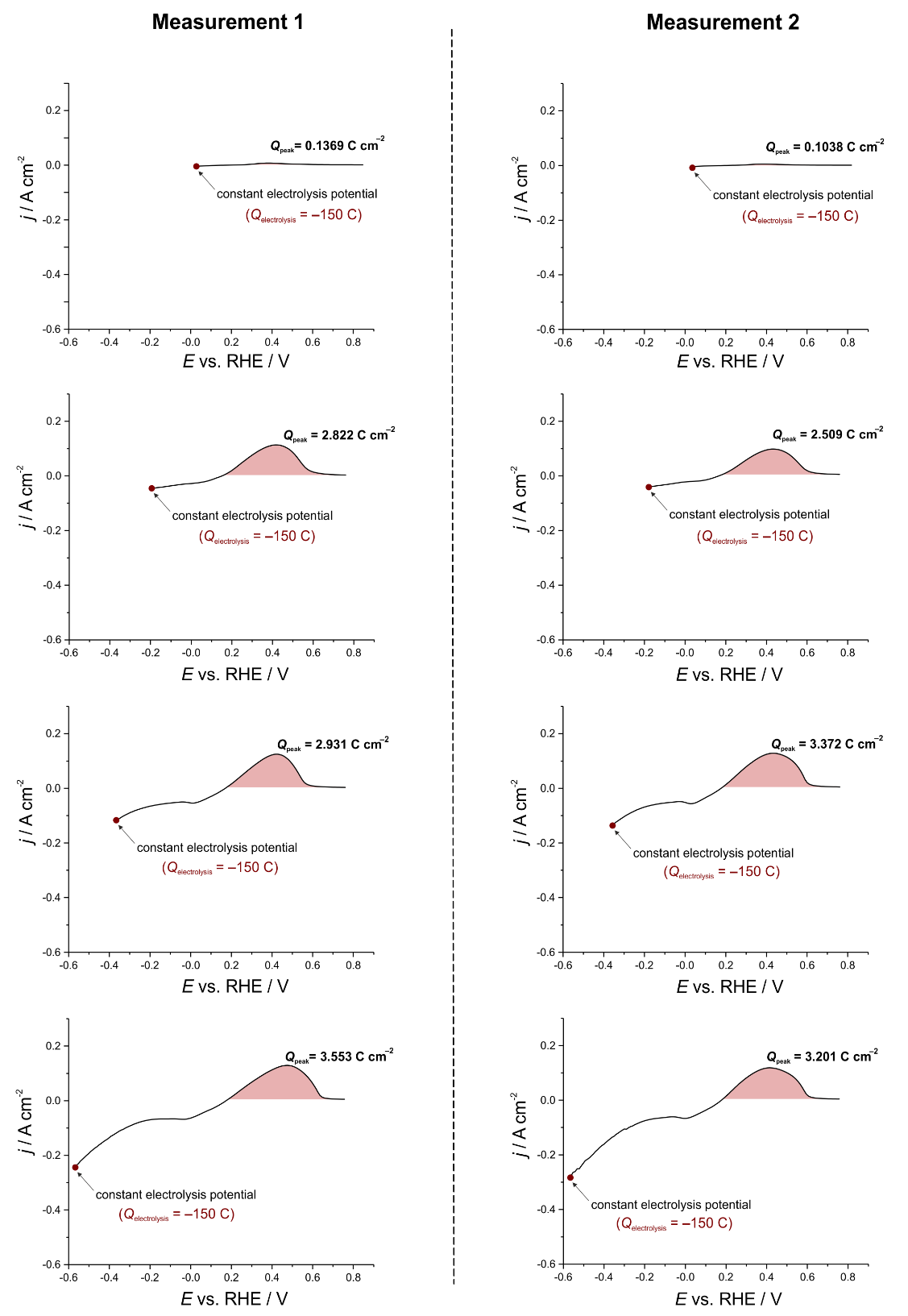


**Figure S28.** Linear sweep voltammograms (d*E*/d*t* = 10 mV s^–1^) following constant charge electrolysis (*Q*_electrolysis_ = –150 C) performed at varying electrolysis potentials. The respective electrolysis potential served as the starting point for the backward LSV passing the oxidation peak assigned to the Co(0)/Co(II) transition. The charge involved in the Co(0)/Co(II) transition was derived through integration after correction for capacitive currents and is indicated next to the peak. Measurements 1 and 2 refer to two independent measurements campaigns. For each electrolysis and backward LSV, a fresh catalyst was used.


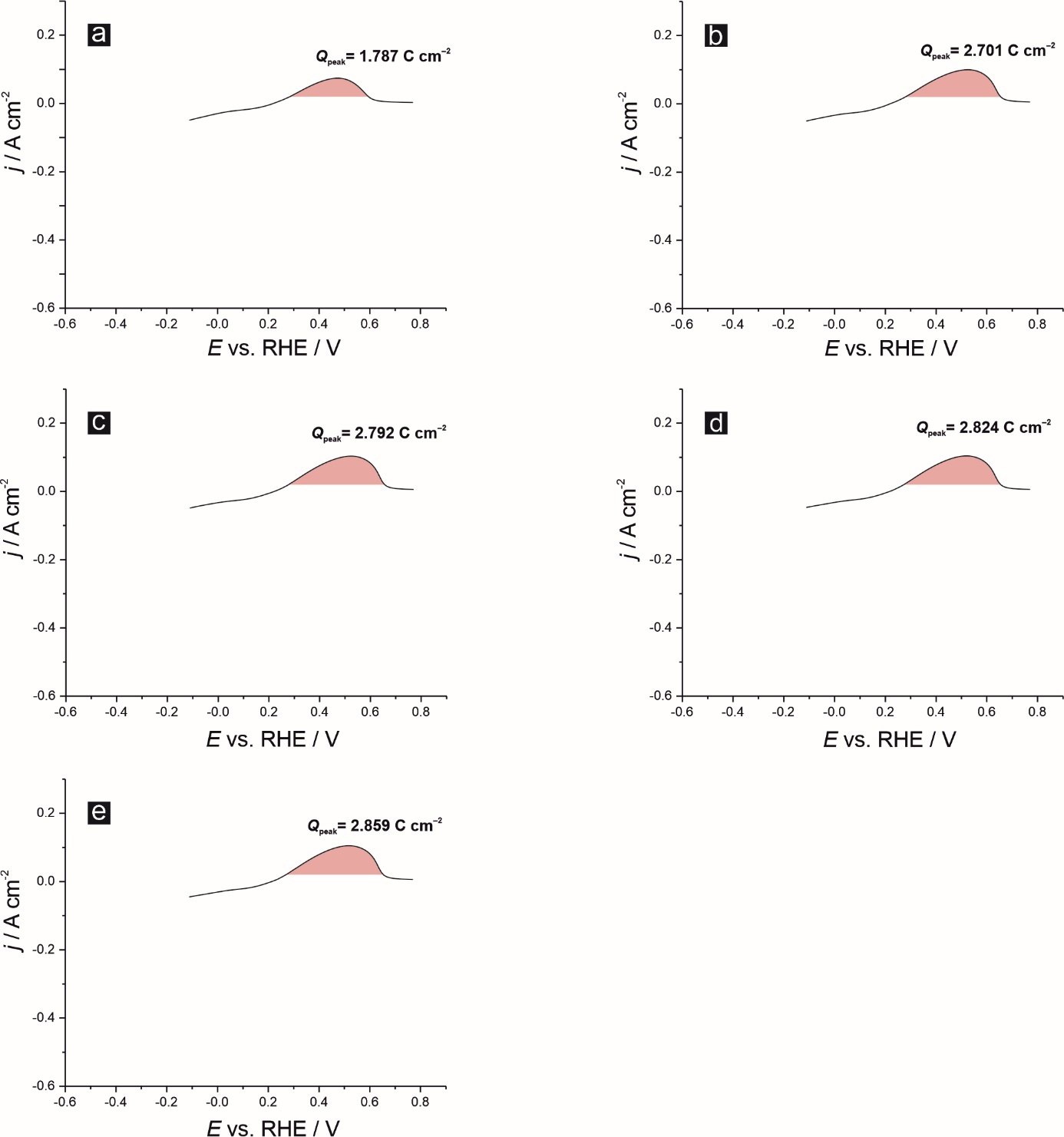


**Figure S29.** Linear sweep voltammograms (d*E*/d*t* = 10 mV s⁻¹) following electrolysis at –0.2 V vs. RHE for progressively extended electrolysis times of 10 minutes (panel a), 30 minutes (panel b), 60 minutes (panel c), 90 minutes (panel d), and 120 minutes (panel e). For the measurement campaign presented, one single catalyst has been used. Each LSV was initiated at the electrolysis potential of –0.2 V vs. RHE and scanned anodically to encompass the oxidation peak attributed to the Co(0)/Co(II) redox transition before returning to the electrolysis potential. The charge involved in the Co(0)/Co(II) transition was derived through peak integration after correction for capacitive currents. Notably, the integrated charges remain effectively invariant across the full time-dependent series, demonstrating the remarkable electrochemical and mechanical stability of the metallic cobalt phase under continuous operation. These findings further indicate that any minor electrolyte or volume changes potentially induced by successive anodic cycling exert a negligible influence on the electrochemically accessible Co(0) content, establishing the robustness of our Co foam catalyst under conditions essential for the combined LSV-ICP-MS approach utilized in Figure 6. These experiments demonstrate that the surface area and the amount of metallic cobalt that undergoes oxidation-reduction cycles do not significantly change during catalyst stressing through repetitive cycling of the electrode potential.


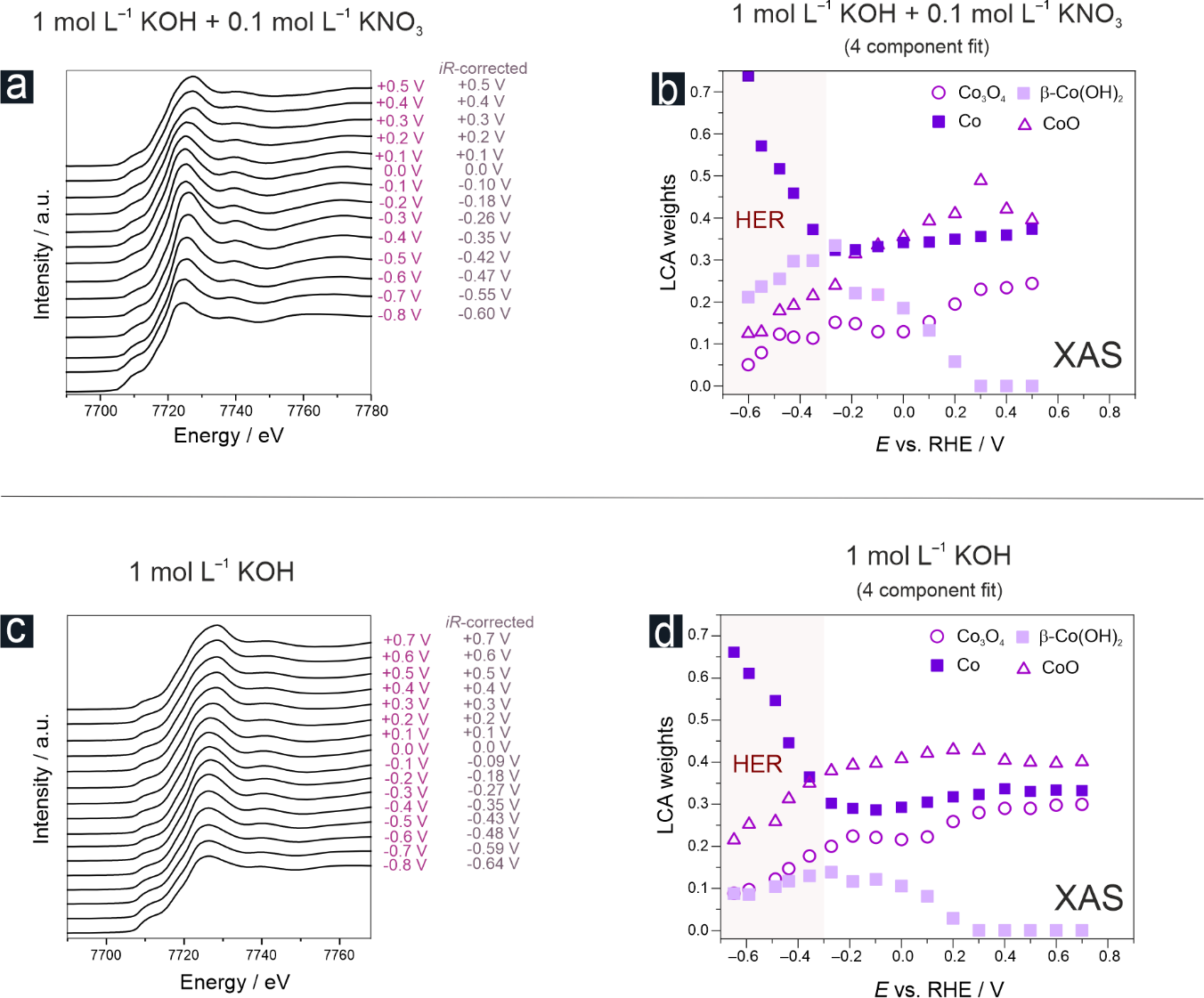


**Figure S30.** a) Potential-dependent operando X-ray absorption spectra (XAS) of the Co/Co*_x_*O*_y_*H*_z_*@C foam exposed to 1 mol L^–1^ KOH + 0.1 mol L^–1^ KNO_3_. b) Corresponding LCA weights derived from operando XAS measurements. c) Potential-dependent operando XAS of the Co/Co*_x_*O*_y_*H*_z_*@C foam exposed to 1 mol L^–1^ KOH. d) Corresponding LCA weights derived from operando XAS measurements. Note that all potential-dependent measurements are *iR*-corrected.


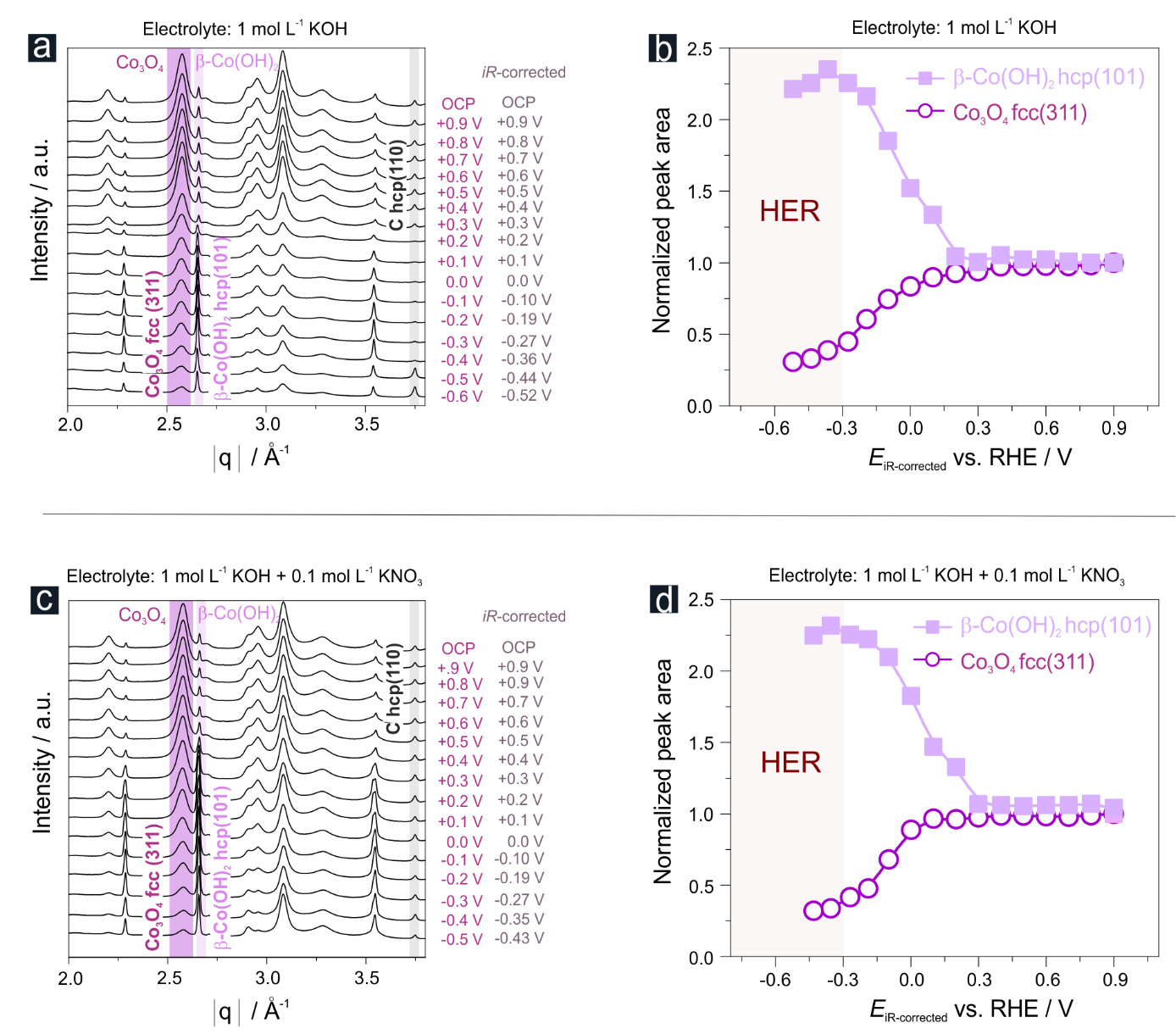


**Figure S31.** a) Potential-dependent operando X-ray diffraction (XRD) spectra of the Co/Co*_x_*O*_y_*H*_z_*@C foam exposed to 1 mol L^–1^ KOH. b) Corresponding normalized peak intensities for Co_3_O_4_(311) and β-Co(OH)_2_(101) derived from the operando XRD measurements. c) Potential-dependent operando XRD of the Co/Co*_x_*O*_y_*H*_z_*@C foam exposed to 1 mol L^–1^ KOH + 0.1 mol L^–1^ KNO_3_. d) Corresponding normalized peak intensities for Co_3_O_4_(311) and β-Co(OH)_2_(101) derived from the operando XRD measurements. Note that all potential dependent measurements are *iR*-corrected.


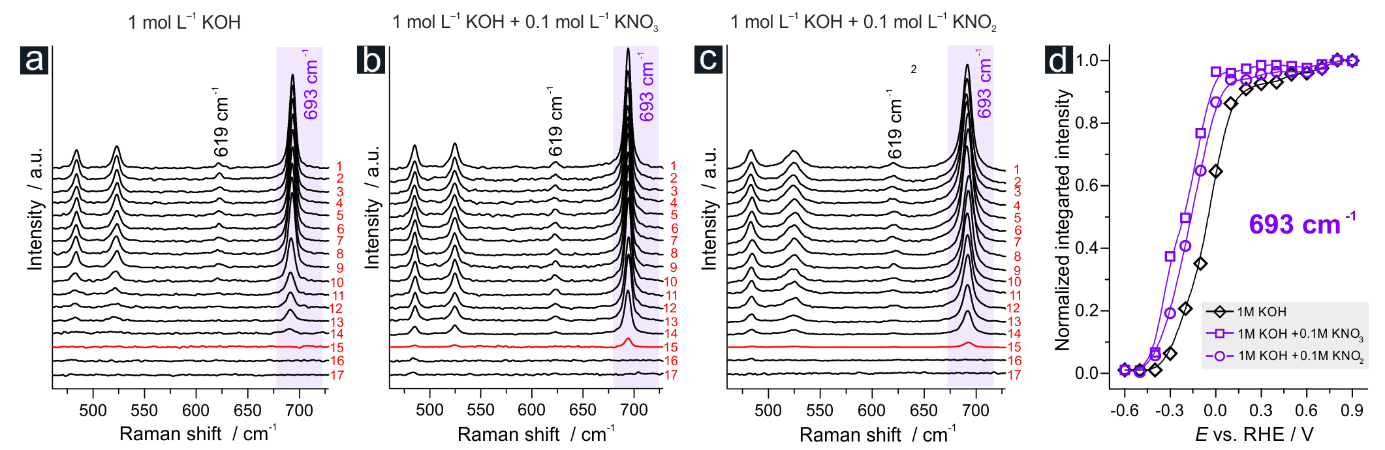


**Figure S32.** a) Potential-dependent operando Raman spectra of the Co/Co*_x_*O*_y_*H*_z_*@C foam exposed to 1 mol L^–1^ KOH electrolyte solution demonstrating the consumption of Co_3_O_4_ phase with increasing cathodic potentials. The series covers the potential range from +0.9 V vs. RHE (spectrum 1) to –0.6 V vs. RHE (spectrum 17). b) Corresponding series of Raman spectra recorded in 1 mol L^–1^ KOH + 0.1 mol L^–1^ KNO_3_ electrolyte solution. c) Corresponding series of Raman spectra recorded in 1 mol L^–1^ KOH + 0.1 mol L^–1^ KNO_2_ electrolyte solution. d) Plot of the normalized integrated intensity of the Raman band at 692 cm^–1^ as a function of the applied electrode potential (*iR*-corrected data). Note that the Co_3_O_4_ phase appears to be stabilized in the presence of nitrate or nitrite in the electrolyte solution. The consumption of the Co_3_O_4_ phase starts at more cathodic potentials when nitrate/nitrite is present. This supports the assumption of a chemisorptive (stabilizing) interaction of the nitrate/nitrite with the cobalt oxide.


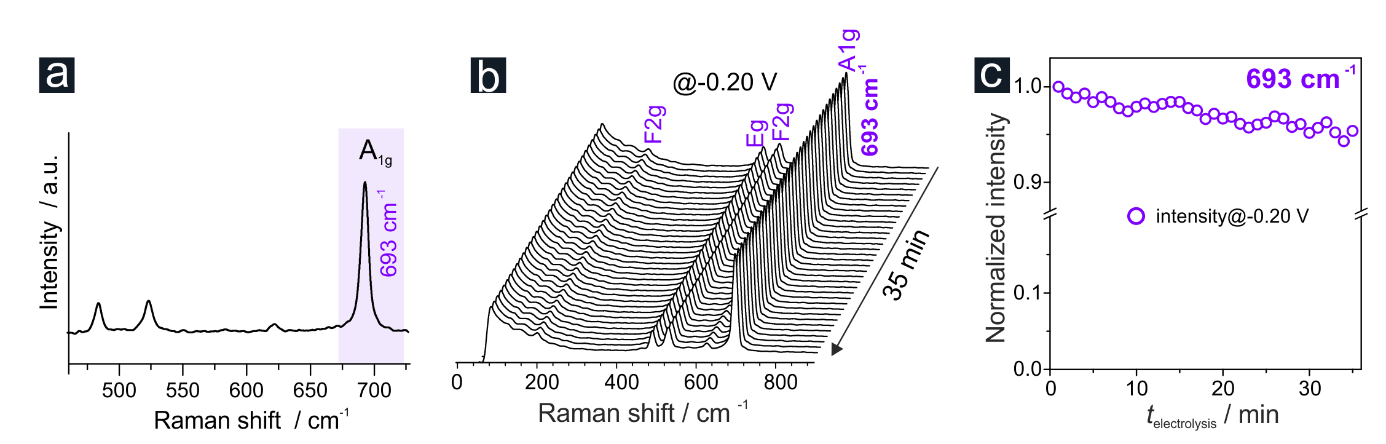


**Figure S33.** a) Representative operando Raman spectrum recorded at *E* = –0.2 V vs. RHE. b) Set of time-dependent operando Raman spectra recorded at *E* = –0.2 V vs. RHE. c) Normalized integrated intensities derived from the spectra shown in panel b.


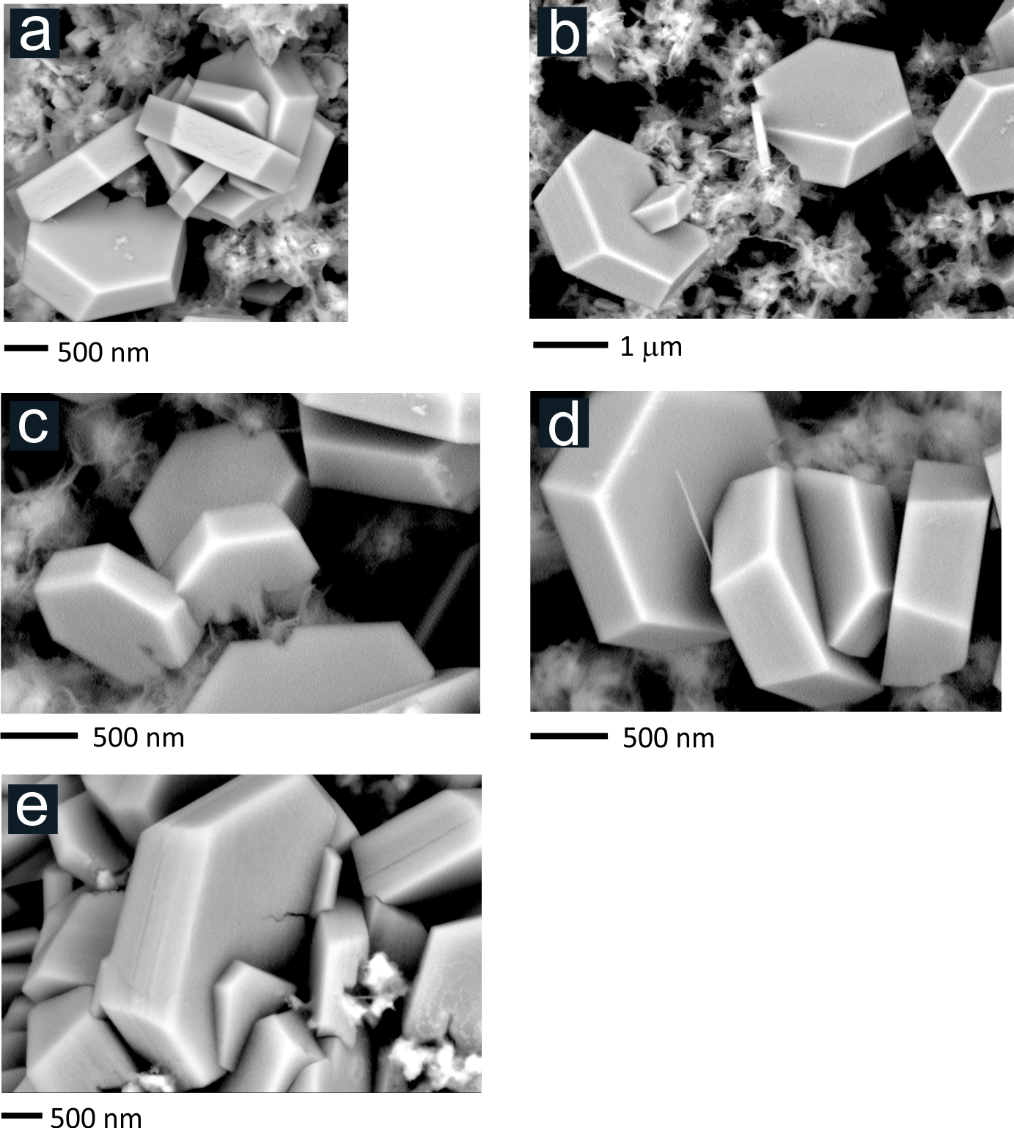


**Figure S34.** Representative top-down SEM images measured after potentiostatic electrolyses in 1 mol L^–1^ KOH + 1 mol L^–1^ KNO_3_ solution (pH 13.7) demonstrating the presence of well-crystallized and hexagonally shaped β-Co(OH)_2_ particles embedded in a disordered composite catalyst matrix. β-Co(OH)_2_ particles are present on the catalyst surface after electrolysis no matter which electrolysis potential has been applied for the electrolysis in the range from 0 V vs. RHE to –0.6 V vs. RHE.


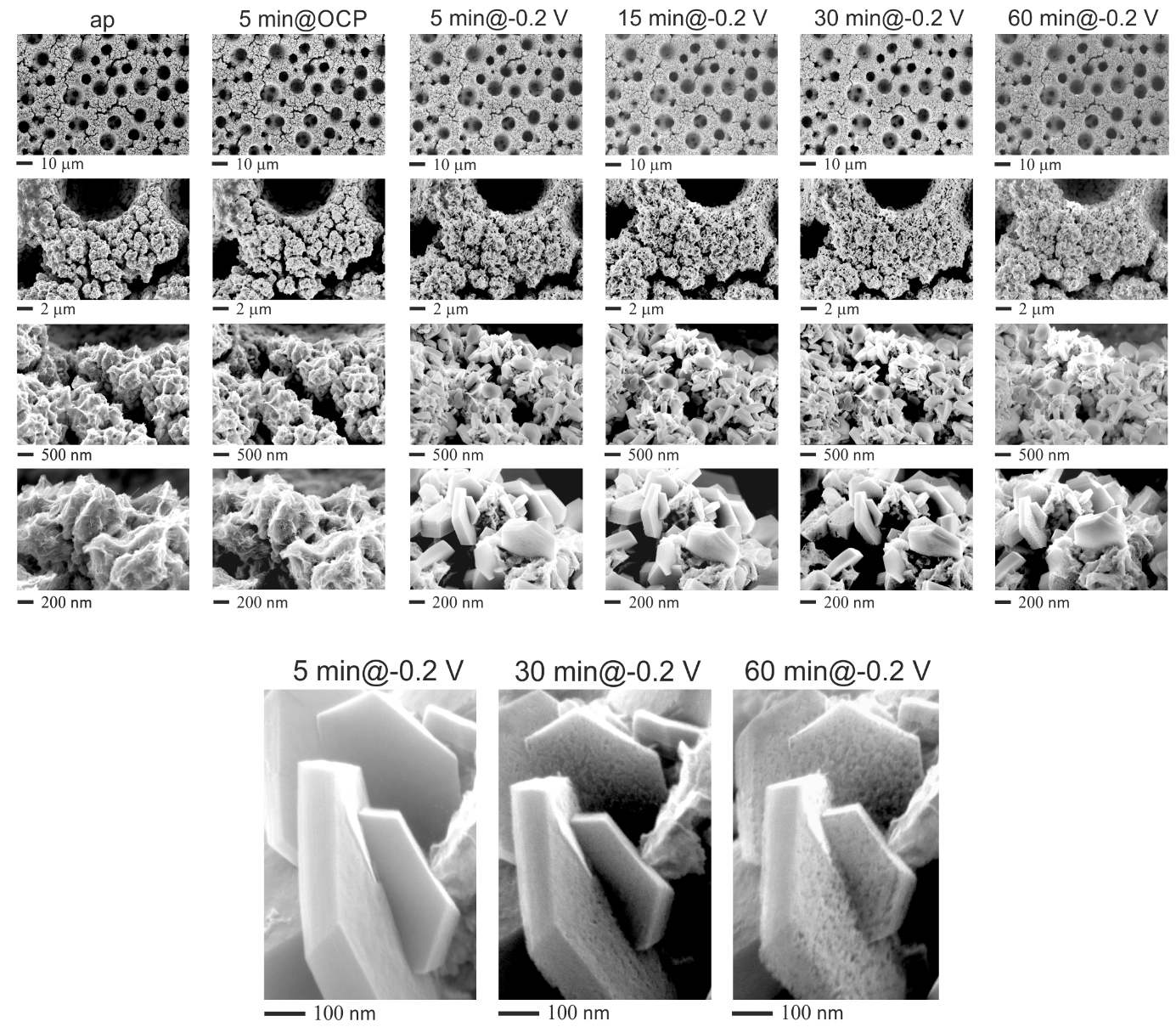


**Figure S35.** Comprehensive identical-location (IL) SEM study on the evolution of the catalyst morphology during electrolysis. The potentiostatic electrolysis carried out at –0.2 V vs. RHE in 1 mol L^–1^ KOH + 0.1 mol L^–1^ KNO_3_ solution (pH 13.7) was interrupted after defined electrolysis times (indicated in the figure) and subjected to SEM analysis before the electrolysis was continued.


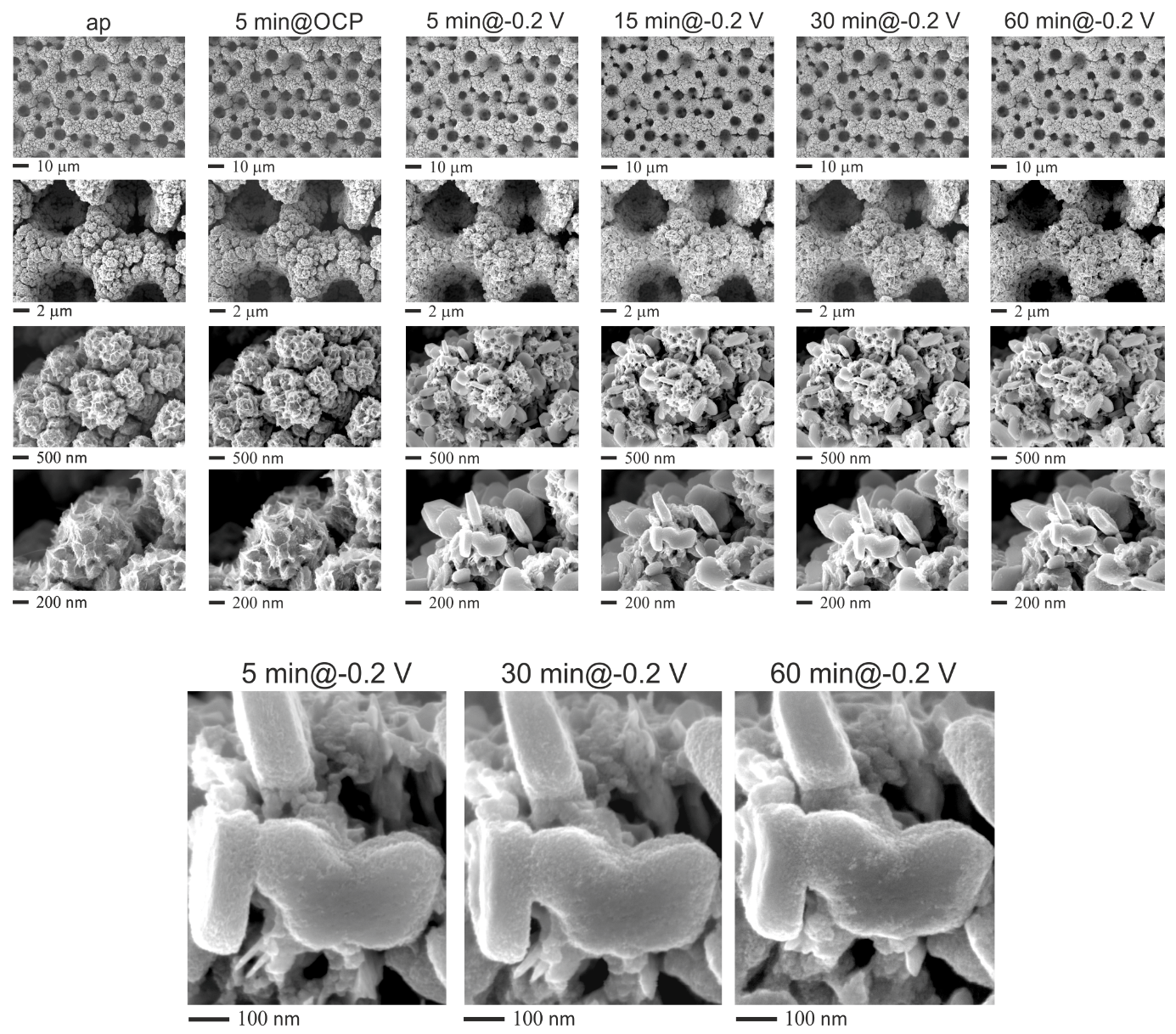


**Figure S36.** Comprehensive identical-location (IL) SEM study on the evolution of the catalyst morphology during electrolysis. The potentiostatic electrolysis carried out at –0.2 V vs. RHE in 1 mol L^–1^ KOH solution (absence of nitrate) was interrupted after defined electrolysis times (indicated in the figure) and subjected to SEM analysis before the electrolysis was continued.

**Table S1.** Foam thickness, pore diameter, and mass loading (derived from gravimetry) of electrodeposited Co/Co(OH)2@Ni foam determined by cross-sectional and top-down SEM. The listed numerical values correspond to Figure S5.

| **Deposition time / s** | **Foam thickness / µm** | **Pore diameter / µm** | **Mass loading / (mg cm^–2^)** |
| --- | --- | --- | --- |
| 5 | 6.7 ± 1.9 | 3.09 ± 0.43 | 1.050 ± 0.050 |
| 20 | 20.2 ± 2.9 | 8.3 ± 1.0 | 4.07 ± 0.17 |
| 40 | 33.9 ± 3.4 | 9.7 ± 1.3 | 7.25 ± 0.25 |
| 60 | 46.4 ± 1.6 | 15.1 ± 2.8 | 10.30 ± 0.20 |

**Table S2a.** Calculated XRD pattern of the Co/Co*_x_*O*_y_*H*_z_* foam prior to the electrolysis (Figure 2f) analyzed using Rietveld refinement with particular emphasis on the diffraction peaks corresponding to metallic cobalt (hexagonal close-packed (hcp) phase, space group P63/mmc).

| **(hkl)** | ***d*-spacing** | **2*Ѳ*** | **FWHM** | **Relative peak abundance / %** |
| --- | --- | --- | --- | --- |
| 100 | 2.17353 | 4.356 | 0.1718 | 22.646 |
| 002 | 2.04084 | 4.639 | 0.17254 | 26.978 |
| 101 | 1.91848 | 4.936 | 0.17331 | 100 |
| 102 | 1.48779 | 6.366 | 0.17707 | 13.785 |
| 110 | 1.25489 | 7.549 | 0.18021 | 14.625 |
| 103 | 1.15325 | 8.215 | 0.18199 | 18.932 |
| 200 | 1.08677 | 8.719 | 0.18335 | 1.986 |
| 112 | 1.06897 | 8.864 | 0.18374 | 15.045 |
| 201 | 1.05018 | 9.023 | 0.18417 | 10.021 |
| 004 | 1.02042 | 9.287 | 0.18489 | 2.006 |
| 202 | 0.95924 | 9.88 | 0.1865 | 2.268 |
| 104 | 0.92369 | 10.262 | 0.18754 | 1.765 |
| 203 | 0.84913 | 11.165 | 0.19002 | 3.649 |
| 210 | 0.82152 | 11.542 | 0.19106 | 1.138 |
| 211 | 0.80537 | 11.774 | 0.1917 | 4.839 |
| 114 | 0.79171 | 11.978 | 0.19227 | 3.314 |
| 105 | 0.76421 | 12.411 | 0.19347 | 1.932 |
| 212 | 0.76209 | 12.445 | 0.19357 | 1.292 |
| 204 | 0.7439 | 12.751 | 0.19442 | 0.625 |
| 300 | 0.72451 | 13.094 | 0.19538 | 1.246 |
| 301 | 0.71336 | 13.299 | 0.19595 | 0.001 |
| 213 | 0.70326 | 13.491 | 0.19649 | 3.803 |

**Table S2b.** Calculated XRD pattern of the Co/Co*_x_*O*_y_*H*_z_* foam prior to the electrolysis (Figure 2f) analyzed using Rietveld refinement with particular emphasis on the diffraction peaks corresponding to metallic cobalt (face center-cubic (fcc) phase, space group Fm-3m).

| **(hkl)** | ***d*-spacing** | **2*Ѳ*** | **FWHM** | **Relative peak abundance / %** |
| --- | --- | --- | --- | --- |
| 111 | 2.0433 | 4.634 | 0.07081 | 100 |
| 200 | 1.76955 | 5.351 | 0.07269 | 39.034 |
| 220 | 1.25126 | 7.571 | 0.07855 | 25.591 |
| 311 | 1.06708 | 8.88 | 0.08206 | 26.289 |
| 222 | 1.02165 | 9.275 | 0.08313 | 7.145 |
| 400 | 0.88478 | 10.714 | 0.08704 | 1.944 |
| 331 | 0.81193 | 11.679 | 0.08968 | 6.782 |
| 420 | 0.79137 | 11.983 | 0.09052 | 6.119 |
| 422 | 0.72242 | 13.132 | 0.0937 | 4.122 |

**Table S2c.** Calculated XRD pattern of the Co/Co*_x_*O*_y_*H*_z_* foam prior to the electrolysis (Figure 2f) analyzed using Rietveld refinement with particular emphasis on the diffraction peaks corresponding to CoO (face center-cubic (fcc) phase, space group Fm-3m).

| **(hkl)** | ***d*-spacing** | **2*Ѳ*** | **FWHM** | **Relative peak abundance / %** |
| --- | --- | --- | --- | --- |
| 111 | 2.4601 | 3.848 | 0.1718 | 64.023 |
| 200 | 2.13051 | 4.444 | 0.17254 | 100 |
| 220 | 1.5065 | 6.286 | 0.17331 | 64.21 |
| 311 | 1.28475 | 7.373 | 0.17707 | 30.369 |
| 222 | 1.23005 | 7.701 | 0.18021 | 15.433 |
| 440 | 1.06526 | 8.895 | 0.18199 | 7.675 |
| 331 | 0.97755 | 9.695 | 0.18335 | 10.82 |
| 420 | 0.95279 | 9.947 | 0.18374 | 18.578 |
| 422 | 0.86978 | 10.899 | 0.18417 | 11.508 |
| 333 | 0.82003 | 11.563 | 0.18489 | 1.767 |
| 511 | 0.82003 | 11.563 | 0.1865 | 5.301 |
| 440 | 0.75325 | 12.592 | 0.18754 | 2.946 |
| 531 | 0.72024 | 13.172 | 0.19002 | 5.575 |
| 442 | 0.71017 | 13.359 | 0.11168 | 6.016 |

**Table S2d.** Calculated XRD pattern of the Co/Co*_x_*O*_y_*H*_z_* foam prior to the electrolysis (Figure 2f) analyzed using Rietveld refinement with particular emphasis on the diffraction peaks corresponding to Co_3_O_4_ (face center-cubic (fcc) phase, space group Fd-3m).

| **(hkl)** | ***d*-spacing** | **2*Ѳ*** | **FWHM** | **Relative peak abundance / %** |
| --- | --- | --- | --- | --- |
| 220 | 2.85873 | 3.312 | 0.08057 | 31.363 |
| 311 | 2.43793 | 3.883 | 0.08204 | 100 |
| 222 | 2.33414 | 4.056 | 0.08249 | 9.396 |
| 400 | 2.02142 | 4.684 | 0.08411 | 21.178 |
| 331 | 1.85499 | 5.105 | 0.08521 | 0.227 |
| 422 | 1.65049 | 5.738 | 0.08687 | 11.833 |
| 333 | 1.55609 | 6.086 | 0.08778 | 5.801 |
| 511 | 1.55609 | 6.086 | 0.08778 | 34.283 |
| 440 | 1.42936 | 6.626 | 0.08921 | 48.739 |
| 442 | 1.36673 | 6.93 | 0.09001 | 1.822 |
| 620 | 1.34762 | 7.029 | 0.09027 | 0.043 |
| 533 | 1.27846 | 7.409 | 0.09128 | 3.819 |
| 622 | 1.23306 | 7.682 | 0.09201 | 8.701 |
| 444 | 1.21896 | 7.771 | 0.09225 | 5.738 |
| 551 | 1.16707 | 8.118 | 0.09317 | 2.92 |
| 711 | 1.13222 | 8.368 | 0.09384 | 1.009 |
| 642 | 1.13222 | 8.368 | 0.09384 | 0.047 |
| 553 | 1.0805 | 8.769 | 0.09492 | 5 |
| 731 | 1.05267 | 9.001 | 0.09555 | 6.418 |
| 800 | 1.05267 | 9.001 | 0.09555 | 7.626 |
| 733 | 1.01071 | 9.376 | 0.09656 | 4.851 |
| 644 | 0.98783 | 9.594 | 0.09715 | 0.008 |
| 660 | 0.98054 | 9.665 | 0.09734 | 0.007 |
| 822 | 0.95291 | 9.946 | 0.0981 | 0.804 |
| 555 | 0.95291 | 9.946 | 0.0981 | 1.212 |
| 751 | 0.93366 | 10.152 | 0.09866 | 1.624 |
| 662 | 0.93366 | 10.152 | 0.09866 | 6.036 |
| 840 | 0.92749 | 10.219 | 0.09884 | 2.123 |
| 753 | 0.90401 | 10.486 | 0.09957 | 1.941 |
| 911 | 0.88752 | 10.681 | 0.1001 | 0.043 |
| 842 | 0.88752 | 10.681 | 0.1001 | 0.224 |
| 664 | 0.88222 | 10.745 | 0.10027 | 0.027 |
| 931 | 0.86194 | 10.999 | 0.10097 | 0.527 |
| 844 | 0.84761 | 11.185 | 0.10148 | 4.665 |
| 771 | 0.82524 | 11.49 | 0.10231 | 8.209 |
| 933 | 0.81264 | 11.668 | 0.1028 | 0.003 |
| 755 | 0.81264 | 11.668 | 0.1028 | 0.094 |
| 862 | 0.81264 | 11.668 | 0.1028 | 0.055 |
| (10)20 | 0.79287 | 11.96 | 0.1036 | 0.847 |
| 773 | 0.79287 | 11.96 | 0.1036 | 0.554 |
| 951 | 0.78167 | 12.132 | 0.10408 | 0.73 |
| 666 | 0.78167 | 12.132 | 0.10408 | 3.858 |
| (10)22 | 0.77805 | 12.189 | 0.10423 | 0.321 |
| 953 | 0.77805 | 12.189 | 0.10423 | 0.982 |
| 864 | 0.754 | 12.579 | 0.10531 | 0.202 |
| (10)42 | 0.75074 | 12.634 | 0.10546 | 0.012 |
| 775 | 0.73812 | 12.851 | 0.10606 | 0.552 |
| (11)11 | 0.72906 | 13.012 | 0.10651 | 0.872 |
| 880 | 0.72906 | 13.012 | 0.10651 | 0.769 |
| 971 | 0.71468 | 13.275 | 0.10723 | 1.938 |
| 955 | 0.70645 | 13.43 | 0.10767 | 0.091 |
| (11)31 | 0.70645 | 13.43 | 0.10767 | 0.199 |
| 882 | 0.70645 | 13.43 | 0.10767 | 0.001 |
| (10)44 | 0.70377 | 13.481 | 0.10781 | 0.031 |

**Table S2e.** Lattice parameters, relative phase abundance, and crystal size derived by Rietveld refinement for the crystalline phases probed in Figure 2f.

| **Phase** | **Lattice parameter (Å)** | **Phase abundance** | **Crystal size / µm** |
| --- | --- | --- | --- |
| Co (hcp) | 2.50978 | 0.4413 | 0.0059 |
| Co (fcc) | 3.53911 | 0.2399 | 0.0163 |
| Co3O4 (fcc) | 8.0857 | 0.08083 | 0.0133 |
| CoO (fcc) | 4.26102 | 0.238 | 0.0125 |

**Table S3.** Numerical data for Figure S21. Total current density, partial current density for ammonia formation, and ammonia Faradaic efficiency for NO_3_RR at Co/Co*_x_*O*_y_*H*_z_*@Ni foams prepared using different deposition times. Data were obtained for 30 minutes of potentiostatic (–0.3 V vs. RHE) electrolysis in 1 mol L^–1^ KOH +
0.1 mol L^–1^ KNO_3_ solution (pH 13.7). For each electrolysis experiment, a newly prepared catalyst was used. The table displays the individual values obtained for each experiment and the average values derived from two independent measurements.

| **Deposition time / s** | **Geometric area-normalized current density / (mA cm^–2^)** | | | | ***FE*_NH₃_ / %** | |
| --- | --- | --- | --- | --- | --- | --- |
|  | **Total** | | **Partial (NH₃)** | |  |  |
| 5 | –59.55  –73.29 | –66.4 ± 6.9 | –46.05  –52.70 | –49.4 ± 3.3 | 77.33  71.91 | 74.6 ± 2.7 |
| 10 | –70.00  –63.30 | –66.7 ± 3.4 | –54.56  –56.95 | –55.8 ± 1.2 | 77.94  89.97 | 84 ± 6 |
| 20 | –75.35  –72.44 | –73.9 ± 1.5 | –69.04  –65.70 | –67.4 ± 1.7 | 91.63  90.69 | 91.16 ± 0.47 |
| 30 | –88.10  –89.89 | –89 ± 0.9 | –75.73  –79.93 | –77.8 ± 2.1 | 85.96  88.92 | 87.4 ± 1.5 |
| 40 | –89.72  –88.33 | –89 ± 0.7 | –75.98  –70.57 | –73.3 ± 2.7 | 84.68  79.89 | 82.3 ± 2.4 |
| 50 | –85.98  –102.92 | –94.5 ± 8.5 | –72.57  –83.81 | –78.2 ± 5.6 | 84.40  81.43 | 82.9 ± 1.5 |
| 60 | –116.32  –94.91 | –106 ± 11 | –89.88  –76.30 | –83.1 ± 6.8 | 77.27  80.39 | 78.8 ± 1.6 |

**Table S4a.** Numerical data for Figure 4a. Potential-dependent total current density, ammonia partial current density, and total electrolysis charge data for NO_3_RR. Data were obtained for 30 minutes of potentiostatic electrolysis in 1 mol L^–1^ KOH + 0.1 mol L^–1^ KNO_3_ solution (pH 13.7). For each electrolysis experiment, a newly prepared catalyst was used. The table displays the individual values obtained for each electrolysis and the average values derived from three independent measurements.

| **Potential / V** | **Geometric area-normalized current density / (mA cm^–2^)** | | | | **Total charge / C** | |
| --- | --- | --- | --- | --- | --- | --- |
|  | **Total** | | **Partial (NH₃)** | |  |  |
| 0 | –33.39 | –23.9 ± 4.8 | –24.74 | –18.4 ± 3.2 | –60.11 | –43 ± 8.7 |
|  | –17.97 |  | –15.00 |  | –32.35 |  |
|  | –20.25 |  | –15.43 |  | –36.45 |  |
| –0.1 | –42.82 | –42.9 ± 4.1 | –38.15 | –39.4 ± 3.3 | –77.07 | –77.2 ± 7.3 |
|  | –35.92 |  | –34.34 |  | –64.67 |  |
|  | –49.99 |  | –45.58 |  | –89.99 |  |
| –0.2 | –59.08 | –59.5 ± 1.6 | –56.01 | –58 ± 2 | –106.34 | –107.1 ± 2.9 |
|  | –57.01 |  | –56.12 |  | –102.62 |  |
|  | –62.49 |  | –62.02 |  | –112.49 |  |
| –0.3 | –64.74 | –70.8 ± 3.2 | –59.97 | –64.9 ± 2.6 | –116.53 | –127.5 ± 5.7 |
|  | –75.35 |  | –69.04 |  | –135.62 |  |
|  | –72.44 |  | –65.70 |  | –130.40 |  |
| –0.4 | –140.19 | –145 ± 14 | –123.26 | –124 ± 10 | –252.35 | –262 ± 26 |
|  | –123.82 |  | –107.29 |  | –222.88 |  |
|  | –172.24 |  | –142.9 |  | –310.04 |  |
| –0.5 | –167.51 | –191 ± 14 | –123.42 | –139 ± 13 | –301.53 | –345 ± 26 |
|  | –189.75 |  | –130.34 |  | –341.56 |  |
|  | –217.12 |  | –164.34 |  | –390.82 |  |
| –0.6 | –383.99 | –399.4 ± 7.9 | –227.61 | –230.4 ± 3.7 | –691.18 | –719 ± 14 |
|  | –404.41 |  | –225.85 |  | –727.95 |  |
|  | –409.77 |  | –237.86 |  | –737.59 |  |

**Table S4b.** Numerical data for Figure 4c.

| **Potential / V** | **Concentration / mM** | | | | | |
| --- | --- | --- | --- | --- | --- | --- |
|  | $\mathbf{N}\mathbf{H}_{\mathbf{3}}$ | | $\mathbf{N}\mathbf{O}_{\mathbf{3}}^{\mathbf{-}}$ | | $\mathbf{N}\mathbf{O}_{\mathbf{2}}^{\mathbf{-}}$ | |
| 0 | 3.85 | 2.9 ± 0.5 | 96.26 | 94.58 ± 0.86 | 0.053 | 0.0523 ± 0.0012 |
|  | 2.33 |  | 93.45 |  | 0.054 |  |
|  | 2.40 |  | 94.02 |  | 0.050 |  |
| –0.1 | 5.93 | 6.12 ± 0.51 | 94.61 | 94.09 ± 0.45 |  | 0 |
|  | 5.34 |  | 94.46 |  |  |  |
|  | 7.09 |  | 93.20 |  |  |  |
| –0.2 | 8.71 | 9.02 ± 0.31 | 88.87 | 91.6 ± 1.4 |  | 0 |
|  | 8.72 |  | 92.26 |  |  |  |
|  | 9.64 |  | 93.71 |  |  |  |
| –0.3 | 9.32 | 10.09 ± 0.41 | 87.71 | 90.7 ± 1.7 |  | 0 |
|  | 10.73 |  | 90.97 |  |  |  |
|  | 10.21 |  | 93.51 |  |  |  |
| –0.4 | 19.16 | 19.4 ± 1.6 | 74.74 | 73.6 ± 1.2 |  | 0 |
|  | 16.68 |  | 74.97 |  |  |  |
|  | 22.22 |  | 71.20 |  |  |  |
| –0.5 | 19.19 | 22.0 ± 2.0 | 70.83 | 68.9 ± 2.3 |  | 0 |
|  | 20.26 |  | 71.57 |  |  |  |
|  | 25.55 |  | 64.36 |  |  |  |
| –0.6 | 35.39 | 35.83 ± 0.58 | 48.38 | 47.32 ± 0.58 |  | 0 |
|  | 35.11 |  | 46.40 |  |  |  |
|  | 36.98 |  | 47.19 |  |  |  |

**Table S4c.** Numerical data for Figure 4d. Potential-dependent total current density and ammonia partial current density data for NO_3_RR. Data were obtained for potentiostatic electrolysis consuming a predefined charge of –150 C in 1 mol L+ KOH + 0.1 mol L+ KNO_3_ solution (pH 13.7). For each electrolysis experiment a newly prepared catalyst was used. The table displays the individual values obtained for each electrolysis and the average values derived from three independent measurements.

| **Potential / V** | **Geometric area-normalized current density / (mA cm^–2^)** | | | |
| --- | --- | --- | --- | --- |
|  | **Total** | | **Partial (NH₃)** | |
| 0 | –26.65 | –28.6 ± 5.7 | –23.05 | –25 ± 4.8 |
|  | –39.37 |  | –34.19 |  |
|  | –19.91 |  | –17.89 |  |
| –0.1 | –44.24 | –44.3 ± 1.5 | –40.46 | –40 ± 1.4 |
|  | –46.83 |  | –42.18 |  |
|  | –41.72 |  | –37.33 |  |
| –0.2 | –59.81 | –56.1 ± 1.9 | –56.42 | –52.4 ± 2.1 |
|  | –54.92 |  | –51.02 |  |
|  | –53.44 |  | –49.64 |  |
| –0.3 | –72.04 | –73.6 ± 4.5 | –65.53 | –68 ± 4.7 |
|  | –82.09 |  | –77.10 |  |
|  | –66.63 |  | –61.49 |  |
| –0.4 | –148.93 | –136 ± 13 | –130.32 | –121 ± 11 |
|  | –109.63 |  | –98.37 |  |
|  | –148.19 |  | –133.39 |  |
| –0.5 | –195.77 | –200 ± 18 | –173.94 | –176 ± 12 |
|  | –233.88 |  | –198.43 |  |
|  | –171.78 |  | –156.21 |  |
| –0.6 | –284.48 | –275 ± 10 | –220.01 | –220 ± 5.5 |
|  | –285.10 |  | –229.57 |  |
|  | –254.19 |  | –210.46 |  |

**Table S4d.** Numerical data for Figure 4f.

| **Potential / V** | **Concentration / (mmol L^–1^)** | | | | | |
| --- | --- | --- | --- | --- | --- | --- |
|  | $\mathbf{N}\mathbf{H}_{\mathbf{3}}$ | | $\mathbf{N}\mathbf{O}_{\mathbf{3}}^{\mathbf{-}}$ | | $\mathbf{N}\mathbf{O}_{\mathbf{2}}^{\mathbf{-}}$ | |
| 0 | 11.21 | 11.37 ± 0.14 | 83.50 | 84.72 ± 0.62 | 0.21 | 0.237 ± 0.022 |
|  | 11.25 |  | 85.10 |  | 0.22 |  |
|  | 11.64 |  | 85.56 |  | 0.28 |  |
| –0.1 | 11.85 | 11.703 ± 0.077 | 85.12 | 83.77 ± 0.82 | 0.04 | 0.027 ± 0.013 |
|  | 11.67 |  | 83.88 |  | 0.00 |  |
|  | 11.59 |  | 82.30 |  | 0.04 |  |
| –0.2 | 12.22 | 12.093 ± 0.063 | 83.81 | 82.71 ± 0.63 |  | 0 |
|  | 12.03 |  | 82.69 |  |  |  |
|  | 12.03 |  | 81.63 |  |  |  |
| –0.3 | 11.79 | 11.97 ± 0.11 | 84.84 | 84.897 ± 0.032 |  | 0 |
|  | 12.17 |  | 84.90 |  |  |  |
|  | 11.96 |  | 84.95 |  |  |  |
| –0.4 | 11.34 | 11.5 ± 0.1 | 86.21 | 85.36 ± 0.42 |  | 0 |
|  | 11.63 |  | 84.94 |  |  |  |
|  | 11.66 |  | 84.94 |  |  |  |
| –0.5 | 11.51 | 11.43 ± 0.23 | 85.87 | 85.56 ± 0.19 |  | 0 |
|  | 10.99 |  | 85.58 |  |  |  |
|  | 11.78 |  | 85.22 |  |  |  |
| –0.6 | 10.02 | 10.39 ± 0.21 | 88.63 | 87.17 ± 0.77 |  | 0 |
|  | 10.43 |  | 86.01 |  |  |  |
|  | 10.73 |  | 86.87 |  |  |  |

**Table S4e.** Numerical data for Figure 4b and 4e.

| **Potential / V** | ***FE*_NH₃_ / %** | | | |
| --- | --- | --- | --- | --- |
|  | **constant time** | | **constant charge** | |
| 0 | 74.09 | 77.9 ± 2.8 | 86.51 | 87.7 ± 1.1 |
|  | 83.47 |  | 86.84 |  |
|  | 76.24 |  | 89.85 |  |
| –0.1 | 89.09 | 92 ± 1.9 | 91.45 | 90.33 ± 0.58 |
|  | 95.59 |  | 90.07 |  |
|  | 91.17 |  | 89.48 |  |
| –0.2 | 94.81 | 97.5 ± 1.4 | 94.34 | 93.37 ± 0.48 |
|  | 98.43 |  | 92.89 |  |
|  | 99.24 |  | 92.89 |  |
| –0.3 | 92.64 | 91.65 ± 0.56 | 90.97 | 92.39 ± 0.85 |
|  | 91.63 |  | 93.92 |  |
|  | 90.69 |  | 92.29 |  |
| –0.4 | 87.92 | 85.8 ± 1.5 | 87.50 | 89.08 ± 0.79 |
|  | 86.65 |  | 89.73 |  |
|  | 82.96 |  | 90.01 |  |
| –0.5 | 73.68 | 72.7 ± 2.1 | 88.85 | 88.2 ± 1.8 |
|  | 68.69 |  | 84.85 |  |
|  | 75.69 |  | 90.93 |  |
| –0.6 | 59.28 | 57.7 ± 1.0 | 77.34 | 80.2 ± 1.6 |
|  | 55.85 |  | 80.52 |  |
|  | 58.05 |  | 82.80 |  |

**Table S4f.** Numerical data for time- and charge-normalized ammonia production rate (yield) corresponding to Tables S4b and S4d.

| **Potential / V** | **NH₃ yield normalized to constant electrolysis…** | | | |
| --- | --- | --- | --- | --- |
|  | **time / (mmol cm^–2^ h^–1^)** | | **charge / (mmol cm^–2^ kC^–1^)** | |
| 0 | 0.12 | 0.087 ± 0.017 | 1.12 | 1.133 ± 0.013 |
|  | 0.07 |  | 1.12 |  |
|  | 0.07 |  | 1.16 |  |
| –0.1 | 0.18 | 0.183 ± 0.015 | 1.18 | 1.17 ± 0.0058 |
|  | 0.16 |  | 1.17 |  |
|  | 0.21 |  | 1.16 |  |
| –0.2 | 0.26 | 0.27 ± 0.01 | 1.22 | 1.2067 ± 0.0067 |
|  | 0.26 |  | 1.20 |  |
|  | 0.29 |  | 1.20 |  |
| –0.3 | 0.28 | 0.303 ± 0.012 | 1.18 | 1.2 ± 0.012 |
|  | 0.32 |  | 1.22 |  |
|  | 0.31 |  | 1.20 |  |
| –0.4 | 0.57 | 0.58 ± 0.049 | 1.13 | 1.153 ± 0.012 |
|  | 0.50 |  | 1.16 |  |
|  | 0.67 |  | 1.17 |  |
| –0.5 | 0.58 | 0.653 ± 0.059 | 1.15 | 1.143 ± 0.023 |
|  | 0.61 |  | 1.10 |  |
|  | 0.77 |  | 1.18 |  |
| –0.6 | 1.06 | 1.073 ± 0.019 | 1.00 | 1.04 ± 0.02 |
|  | 1.05 |  | 1.04 |  |
|  | 1.11 |  | 1.07 |  |

**Table S5a.** Numerical data for Figure S24a. Potential-dependent total current density, ammonia partial current density, and total electrolysis charge data for NO_2_RR. Data were obtained for 30 minutes of potentiostatic electrolysis in 1 mol L^–1^ KOH + 0.1 mol L^–1^ KNO_2_ solution (pH 13.7). For each electrolysis experiment, a newly prepared catalyst was used. The table displays the individual values obtained for each electrolysis and the average values derived from two independent measurements.

| **Potential / V** | **Geometric area-normalized current density / (mA cm^–2^)** | | | | **Total charge / C** | |
| --- | --- | --- | --- | --- | --- | --- |
|  | **Total** | | **Partial (NH₃)** | |  |  |
| 0 | –21.78 | –31.3 ± 9.5 | –19.21 | –26.8 ± 7.5 | –39.20 | –56 ± 17 |
|  | –40.84 |  | –34.28 |  | –73.52 |  |
| –0.1 | –33.58 | –40.0 ± 6.4 | –32.75 | –36.6 ± 3.9 | –60.45 | –72 ± 12 |
|  | –46.42 |  | –40.51 |  | –83.56 |  |
| –0.2 | –51.14 | –51.35 ± 0.21 | –51.93 | –53.2 ± 1.3 | –92.05 | –92.43 ± 0.38 |
|  | –51.56 |  | –54.55 |  | –92.81 |  |
| –0.3 | –60.73 | –60.08 ± 0.66 | –64.14 | –62.9 ± 1.3 | –109.32 | –108.1 ± 1.2 |
|  | –59.42 |  | –61.55 |  | –106.95 |  |
| –0.4 | –107.57 | –102.2 ± 5.4 | –99.45 | –94.2 ± 5.3 | –193.63 | –183.9 ± 9.7 |
|  | –96.76 |  | –88.93 |  | –174.17 |  |
| –0.5 | –191.31 | –165 ± 26 | –148.81 | –135 ± 14 | –344.36 | –298 ± 47 |
|  | –139.59 |  | –121.81 |  | –251.26 |  |
| –0.6 | –227.82 | –270 ± 42 | –154.90 | –154.95 ± 0.055 | –410.08 | –485 ± 75 |
|  | –311.52 |  | –155.01 |  | –560.73 |  |

**Table S5b.** Numerical data for Figure S24c.

| **Potential / V** | **Concentration / (mmol L^–1^)** | | | | | |
| --- | --- | --- | --- | --- | --- | --- |
|  | $\mathbf{N}\mathbf{H}_{\mathbf{3}}$ | | $\mathbf{N}\mathbf{O}_{\mathbf{3}}^{\mathbf{-}}$ | | $\mathbf{N}\mathbf{O}_{\mathbf{2}}^{\mathbf{-}}$ | |
| 0 | 3.98 | 5.5 ± 1.6 | 0.83 | 0.825 ± 0.005 | 93.64 | 92.93 ± 0.71 |
|  | 7.11 |  | 0.82 |  | 92.22 |  |
| –0.1 | 6.79 | 7.6 ± 0.8 | 1.03 | 1.05 ± 0.02 | 85.29 | 85.03 ± 0.26 |
|  | 8.40 |  | 1.07 |  | 84.77 |  |
| –0.2 | 10.76 | 11.04 ± 0.28 | 0.96 | 1.12 ± 0.16 | 87.74 | 89.8 ± 2.1 |
|  | 11.31 |  | 1.27 |  | 91.87 |  |
| –0.3 | 13.30 | 13.03 ± 0.27 | 0.77 | 0.825 ± 0.055 | 84.00 | 84.66 ± 0.66 |
|  | 12.76 |  | 0.88 |  | 85.32 |  |
| –0.4 | 18.43 | 24.6 ± 6.2 | 1.07 | 0.92 ± 0.16 | 73.22 | 73.18 ± 0.04 |
|  | 30.85 |  | 0.76 |  | 73.14 |  |
| –0.5 | 30.85 | 28.1 ± 2.8 | 0.76 | 0.79 ± 0.03 | 58.04 | 62 ± 4 |
|  | 25.25 |  | 0.82 |  | 65.97 |  |
| –0.6 | 32.11 | 32.12 ± 0.01 | 0.70 | 0.35 ± 0.35 | 53.09 | 49.6 ± 3.5 |
|  | 32.13 |  | 0.00 |  | 46.05 |  |

**Table S5c.** Numerical data for Figure S24d. Potential-dependent total current density and ammonia partial current density data for NO_2_RR. Data were obtained for potentiostatic electrolysis consuming a predefined charge of –150 C in 1 mol L+ KOH + 0.1 mol L+ KNO_2_ solution (pH 13.7). For each electrolysis experiment a newly prepared catalyst was used. The table displays the individual values obtained for each electrolysis and the average values derived from two independent measurements.

| **Potential / V** | **Geometric area-normalized current density / (mA cm^–2^)** | | | |
| --- | --- | --- | --- | --- |
|  | **Total** | | **Partial (NH₃)** | |
| 0 | –33.57 | –33.31 ± 0.26 | –29.04 | –27.4 ± 1.6 |
|  | –33.06 |  | –25.86 |  |
| –0.1 | –44.19 | –48.7 ± 5.0 | –40.41 | –44.8 ± 4.4 |
|  | –54.15 |  | –49.23 |  |
| –0.2 | –53.43 | –52.92 ± 0.51 | –50.41 | –50.34 ± 0.07 |
|  | –52.41 |  | –50.27 |  |
| –0.3 | –57.05 | –55.64 ± 1.4 | –51.90 | 50.6 ± 1.3 |
|  | –54.30 |  | –49.27 |  |
| –0.4 | –82.86 | –89.7 ± 7.5 | –72.51 | –80.4 ± 7.9 |
|  | –97.77 |  | –88.26 |  |
| –0.5 | –116.62 | –150 ± 47 | –103.62 | –144 ± 40 |
|  | –210.88 |  | –184.25 |  |
| –0.6 | –252.04 | –264 ± 12 | –194.91 | –204.7 ± 9.8 |
|  | –276.13 |  | –214.49 |  |

**Table S5d.** Numerical data for Figure S24f.

| **Potential / V** | **Concentration / (mmol L^–1^)** | | | | | |
| --- | --- | --- | --- | --- | --- | --- |
|  | $\mathbf{N}\mathbf{H}_{\mathbf{3}}$ | | $\mathbf{N}\mathbf{O}_{\mathbf{3}}^{\mathbf{-}}$ | | $\mathbf{N}\mathbf{O}_{\mathbf{2}}^{\mathbf{-}}$ | |
| 0 | 15.97 | 14.7 ± 1.2 | 0.78 | 1.53 ± 0.75 | 88.65 | 84.9 ± 3.8 |
|  | 13.51 |  | 2.28 |  | 81.06 |  |
| –0.1 | 16.20 | 15.96 ± 0.24 | 0.76 | 1.50 ± 0.74 | 90.18 | 84.7 ± 5.4 |
|  | 15.71 |  | 2.24 |  | 79.29 |  |
| –0.2 | 16.15 | 16.36 ± 0.21 | 7.18 | 4.6 ± 2.6 | 90.21 | 86 ± 4 |
|  | 16.57 |  | 2.03 |  | 82.30 |  |
| –0.3 | 15.67 | 15.67 ± 0.0 | 0.85 | 1.53 ± 0.69 | 90.63 | 90.87 ± 0.24 |
|  | 15.67 |  | 2.22 |  | 91.11 |  |
| –0.4 | 15.87 | 15.74 ± 0.14 | 0.75 | 1.16 ± 0.41 | 89.34 | 88 ± 1 |
|  | 15.60 |  | 1.57 |  | 87.26 |  |
| –0.5 | 14.78 | 14.93 ± 0.16 | 1.10 | 1.03 ± 0.07 | 87.88 | 87 ± 1 |
|  | 15.09 |  | 0.96 |  | 85.88 |  |
| –0.6 | 13.44 | 13.43 ± 0.01 | 2.00 | 2.24 ± 0.24 | 90.20 | 90.135 ± 0.065 |
|  | 13.42 |  | 2.48 |  | 90.07 |  |

**Table S5e.** Numerical data for Figure S24b and S24e.

| **Potential / V** | ***FE*_NH₃_ / %** | | | |
| --- | --- | --- | --- | --- |
|  | **constant time** | | **constant charge** | |
| 0 | 88.22 | 86.1 ± 2.1 | 86.51 | 82.4 ± 4.1 |
|  | 83.93 |  | 78.24 |  |
| –0.1 | 97.52 | 92.4 ± 5.1 | 91.45 | 91.19 ± 0.27 |
|  | 87.26 |  | 90.92 |  |
| –0.2 | 101.55 | 103.7 ± 2.1 | 94.34 | 95.13 ± 0.79 |
|  | 105.79 |  | 95.92 |  |
| –0.3 | 105.62 | 105 ± 1 | 90.97 | 90.85 ± 0.12 |
|  | 103.58 |  | 90.74 |  |
| –0.4 | 92.45 | 92.18 ± 0.27 | 87.50 | 88.9 ± 1.4 |
|  | 91.90 |  | 90.28 |  |
| –0.5 | 77.78 | 82.5 ± 4.7 | 88.85 | 88.11 ± 0.74 |
|  | 87.26 |  | 87.37 |  |
| –0.6 | 67.99 | 58.9 ± 9.1 | 77.34 | 77.51 ± 0.17 |
|  | 49.76 |  | 77.68 |  |

**Table S5f.** Numerical data for time- and charge-normalized ammonia production rate (yield) corresponding to Tables S5b and S5d.

| **Potential / V** | **NH₃ yield normalized to constant electrolysis…** | | | |
| --- | --- | --- | --- | --- |
|  | **time / (mmol cm^–2^ h^–1^)** | | **charge / (mmol cm^–2^ kC^–1^)** | |
| 0 | 0.12 | 0.165 ± 0.045 | 1.60 | 1.48 ± 0.13 |
|  | 0.21 |  | 1.35 |  |
| –0.1 | 0.20 | 0.225 ± 0.025 | 1.62 | 1.595 ± 0.025 |
|  | 0.25 |  | 1.57 |  |
| –0.2 | 0.32 | 0.33 ± 0.01 | 1.62 | 1.64 ± 0.02 |
|  | 0.34 |  | 1.66 |  |
| –0.3 | 0.40 | 0.39 ± 0.01 | 1.57 | 1.57 |
|  | 0.38 |  | 1.57 |  |
| –0.4 | 0.62 | 0.585 ± 0.035 | 1.59 | 1.575 ± 0.015 |
|  | 0.55 |  | 1.56 |  |
| –0.5 | 0.93 | 0.845 ± 0.085 | 1.48 | 1.495 ± 0.015 |
|  | 0.76 |  | 1.51 |  |
| –0.6 | 0.96 | 0.96 | 1.34 | 1.34 |
|  | 0.96 |  | 1.34 |  |

**Table S6.** Numerical data for Figure S25. (Long term electrolysis at –0.3 V vs. RHE in 1 mol L^–1^ KOH + 0.1 mol L^–1^ KNO_3_ electrolyte solution, pH 13.7). The current densities were normalized to the geometric surface area. “Initial” values (determined 60 min after start of the respective continuous electrolysis) are highlighted in red.

| **Time / h** | ***j*_tot_  / (mA cm^–2^)** | ***j*_NH₃_  / (mA cm^–2^)** | $\left[ \mathbf{NO}_{\mathbf{3}}^{\mathbf{-}} \right]$ **/ (mM L^–1^)** | $\left[ \mathbf{NH}_{\mathbf{3}}^{\mathbf{-}} \right]$ **/ (mM L^–1^)** | ***FE*_NH₃_  / %** | **Integral NH₃ yield / (mmol cm^–2^)** |
| --- | --- | --- | --- | --- | --- | --- |
| 1 | –83.67 | –82.71 | 84.79 | 8.98 | 98.85 | 0.36 |
| 2 | –72.66 | –57.21 | 81.47 | 12.68 | 78.74 | 0.51 |
| 3 | –67.34 | –51.16 | 74.24 | 17.23 | 75.98 | 0.69 |
| 4 | –63.42 | –46.09 | 70.83 | 20.83 | 72.67 | 0.83 |
| 5 | –60.08 | –42.44 | 65.80 | 24.08 | 70.65 | 0.96 |
| 6 | –57.32 | –37.96 | 64.48 | 25.89 | 66.23 | 1.04 |
| 7 | –83.89 | –82.35 | 90.31 | 8.930 | 98.16 | 1.39 |
| 8 | –78.69 | –61.72 | 82.14 | 13.73 | 78.44 | 1.58 |
| 9 | –74.64 | –56.06 | 72.64 | 18.94 | 75.11 | 1.79 |
| 10 | –70.93 | –50.80 | 66.89 | 23.03 | 71.61 | 1.96 |
| 11 | –67.14 | –48.02 | 64.68 | 27.33 | 71.52 | 2.13 |
| 12 | –63.70 | –40.77 | 60.21 | 27.86 | 64.00 | 2.15 |
| 13 | –83.69 | –82.13 | 86.92 | 8.910 | 98.13 | 2.51 |
| 14 | –78.95 | –64.5 | 82.84 | 14.37 | 81.70 | 2.73 |
| 15 | –74.86 | –52.31 | 77.34 | 17.63 | 69.88 | 2.86 |
| 16 | –70.89 | –48.75 | 72.21 | 20.28 | 68.77 | 2.96 |
| 17 | –66.98 | –44.92 | 68.38 | 25.53 | 67.07 | 3.17 |
| 18 | –63.73 | –38.25 | 65.94 | 26.09 | 60.01 | 3.19 |
| 19 | –73.42 | –72.54 | 87.51 | 7.790 | 98.79 | 3.51 |
| 20 | –71.02 | –62.45 | 81.50 | 13.90 | 87.93 | 3.75 |
| 21 | –68.31 | –46.87 | 77.57 | 15.73 | 68.62 | 3.82 |
| 22 | –67.13 | –45.12 | 68.66 | 20.38 | 67.21 | 4.01 |
| 23 | –64.21 | –42.39 | 67.72 | 24.05 | 66.01 | 4.16 |
| 24 | –61.84 | –35.67 | 66.54 | 24.29 | 57.69 | 4.17 |
